# Comorbid Schizophrenia and Psychotic Symptoms in Patients With Bipolar Disorder: A Meta‐Analysis of the Global Literature

**DOI:** 10.1111/bdi.70093

**Published:** 2026-03-18

**Authors:** Wen Shao, Kangning Shao, Richard P. Bentall

**Affiliations:** ^1^ Department of Psychology University of Sheffield Sheffield UK; ^2^ The Second Affiliated Hospital of Zhengzhou University Zhengzhou China

**Keywords:** bipolar, prevalence, psychosis, schizophrenia

## Abstract

**Background:**

The boundary between bipolar disorder and schizophrenia has long been blurred by the shared psychopathology, genetic risk, and social factors. This study aims to examine the prevalence of psychosis and psychotic symptoms in bipolar patients.

**Method:**

Key words ‘bipolar’, ‘psychosis’, ‘schizophrenia’, and the variants were searched in titles and abstracts using Medline, Psych INFO and Web of Science and forward and backward citation searches were conducted; effects were computed using single proportion analysis with double arcsine transformation.

**Results:**

The final analysis comprised 285 studies. The comorbidity of schizophrenia diagnoses in bipolar patients was low (8%). However, more broadly defined mood incongruent psychosis was quite prevalent (47%). Similarly, psychotic symptoms were common in bipolar patients, specifically those with a type I diagnosis or manic episode; delusions were more common than hallucinations and thought disorders.

**Conclusions:**

Significant overlap in phenomenology and psychopathology was observed between bipolar disorder and schizophrenia in this review. Future research should focus on comparing patients with similar symptoms and exploring the shared processes that contribute to these symptoms.

## 1 Introduction

Following Kraepelin's (1908) proposal of a distinction between manic‐depressive illness and dementia praecox, the boundaries between the two conditions have been extensively debated. In contemporary diagnostic manuals, which have been developed from Kraepelin's model, bipolar spectrum disorder (BSD) is characterised by episodes of manic (e.g., euphoria, impulsivity, increased energy) and depressive symptoms (e.g., depressed mood, hopeless, fatigue), whereas schizophrenia is described in terms of altered thought processes and perceptions, especially delusions (i.e., fixed false beliefs), hallucinations (i.e., illusory perceptions) and negative symptoms such as avolition [1, 2]. Although this distinction has been described as one of the cornerstones of modern psychiatry [3], it has been persistently contested by those who have proposed alternative models, or who have doubted the value of diagnostic classifications altogether [4]. For example, some authors have proposed a unitary psychosis model, in which BSD and schizophrenia are considered variants of the same disease [5], some have proposed a third type of schizoaffective psychosis characterised by symptoms of both conditions [6], and some have suggested that there is a boundaryless continuum between BSD and schizophrenia [7].

Similarities between BSD and schizophrenia are evident at many levels of analysis. For example, although empirical research has found a high level of global cognitive impairments in patients across the psychosis spectrum, some studies have attempted to identify specific profiles of neurocognitive dysfunction in the two groups. However, there is evidence that these are more predicted by developmental difficulties than diagnosis [8]. A recent meta‐analysis examined executive functions indexed by reasoning capacities and semantic inhibition, and found that bipolar and schizophrenia patients were similarly impaired [9]. Similar impairments have also been reported for capacities relevant to executive function such as attention, abstract thinking, and working memory [10]. Other neurocognitive studies have suggested that patients with schizophrenia are more severely impaired than bipolar patients on specific working memory tasks and that poorer motor speed and memory retrieval are reported in schizophrenia patients in the majority of the reviewed studies [11]. Despite these general similarities and differences, cognitive impairments are heterogeneous across both populations, perhaps reflecting heterogeneity in both psychopathologies and research methods.

Recent research has also highlighted similarities between the two conditions at the genetic level. Although high heritability estimates have been calculated for both BSD [12] and schizophrenia [13], family studies have shown that they are inter‐heritable, so that there are elevated rates of schizophrenia in the close relatives of BSD patients and vice versa [14, 15, 16]. Overlap between the two diagnostic groups has also been observed at the molecular level [17, 18]. For example, a recent large‐scale study identified 114 shared genome‐wide association (GWA) loci between schizophrenia and bipolar patients [19], and similar findings have been reported elsewhere [20]. Studies using polygenic risk scores support these conclusions. For example, individuals with the highest schizophrenia PRS also have heightened bipolar PRS [21].

Finally, the two conditions may also be similar in respect to social determinants. For example, in recent years there has been considerable research demonstrating that adversity and, in particular, interpersonal trauma in childhood is a risk factor for psychosis [22]. Although fewer studies have been conducted on childhood trauma in BSD patients, those that are available have reported a similar association [23].

It is perhaps not surprising, therefore, that there have been reports of overlapping symptomology in the two conditions. Patients with a primary diagnosis of schizophrenia have been reported to experience manic and depressive symptoms, with varied prevalence across studies [24, 25]. Similarly, the symptoms typically attributed to schizophrenia have been reported in BSD patients. For example, Black and Nasrallah [26] reported a prevalence of current delusions of 36.15% in a large sample of bipolar patients, and even higher estimates have been reported when grandiose delusions are specifically considered. In a recent study, Shao, Simmonds‐Buckley, Zavlis and Bentall [27] also identified highly similar symptom structures of mood and psychosis among patients diagnosed with schizophrenia, schizoaffective disorder, and psychotic BSD. Although existing diagnostic criteria emphasise the chronic nature of psychosis in schizophrenia, some studies have also found persistent psychosis and corresponding deficits in patients with BSD [28, 29].

Given these considerable overlaps and complexities in their illness trajectories, comorbidity between BSD and schizophrenia over the course of illness has been increasingly documented. It is important to note that, when we use the term ‘comorbidity’ in this context, we are doing so in a theoretically neutral way, simply to indicate the extent to which individuals who meet the criteria for one disorder also meet the criteria for another, without prejudice as to whether the two conditions are one, or whether it is possible to suffer from two separate psychiatric disorders at the same time. Hence, this phenomenon is observed in various forms, including: (1) dual diagnoses of comorbid BSD and schizophrenia, where patients meet the diagnostic criteria for both conditions [30, 31]; (2) lifetime comorbidity, in which a patient diagnosed with BSD had a history of schizophrenia, or vice versa [32]; and (3) diagnostic conversion, where a diagnosis of BSD may precede a diagnosis of schizophrenia [33]; in a broader sense, it can also be observed (4) when features once considered distinctive to schizophrenia—such as first‐rank symptoms and mood‐incongruent psychotic features—are also prevalent in BSD [34].

In order to further clarify the relationship between the two conditions, a starting point will be to quantify the extent of this phenomenon. In this study, we synthesise existing studies on the prevalence of comorbid schizophrenia and related symptoms in patients with Bipolar I and Bipolar II diagnoses. A previous synthesis by Chakrabarti and Singh [34] summarised studies of this kind in a narrative systematic review but did not carry out a quantitative synthesis or address heterogeneity between their included studies; they reported various outcomes including general psychosis, first rank symptoms and different kinds of delusions and hallucinations. Although the range was wide, the median and mean values were quite high for conditions such as general psychosis and delusions. Another recent review article did report an empirical synthesis of data on psychotic symptoms in bipolar patients, but only considered lifetime and point‐prevalence rates of hallucinations and delusions in various bipolar populations [35]; they found the psychotic symptoms were prevalent in bipolar populations especially for patients suffering from bipolar I disorder.

In this study we meta‐analyse lifetime and point prevalence rates for schizophrenia in bipolar patients, and also lifetime and point prevalence rates for delusions, hallucinations and thought disorder. We quantify the heterogeneity in the studies, considering whether different clinical variables (e.g., BDI or BDII, euthymic or acute onset) or demographic variables (e.g., age, gender) contributed to the variance observed in this population. We aim to answer the following questions: (1) How prevalent is comorbid schizophrenia in bipolar patients? (2) How prevalent are general psychosis/psychotic symptoms in this population? (3) How prevalent are specific psychotic symptoms such as delusions, hallucinations and thought disorder in this population?

## 2 Materials and Methods

### 2.1 Protocol and Registration

The current study was preregistered on the PROSPERO (https://www.crd.york.ac.uk/PROSPERO/), an international database of prospectively registered systematic reviews in health and other fields (registration number: CRD42022309219).

### 2.2 Eligibility Criteria

Studies were included if they fulfilled the following criteria: (1) the study population had a valid current or lifetime diagnosis of bipolar disorder; (2) the recruited participants were aged 16 years old or older; (3) the prevalence or number of patients with comorbid schizophrenia spectrum disorder, co‐occurring psychosis/psychotic features or symptoms, hallucination, delusion or thought disorder were reported; (4) the study was published after 1980 (the release date of DSM III); (5) the article was peer‐reviewed; and (6) was published in English. Studies were not considered for this data synthesis if the following criteria were met: (1) participants were genetically related (in which case, comorbidity rates might be inflated); (2) participants were diagnosed with psychiatric conditions as a consequence of medical illnesses (e.g., head trauma, pregnancy); (3) they were case reports; (4) the sample size was less than 30; (5) duplicated analyses from the same sample.

To avoid duplication of the included data, the following criteria were applied during the full‐text screening. If two or more studies shared (1) the same sponsor or affiliation, (2) identical name of research program or group and (3) overlapped sampling time, they were considered at high risk of duplication. In these circumstances, only the study reporting the most comprehensive features of the sample were retained in the database. An inquiry email was sent to the authors if information relevant to these criteria was not included in the articles.

### 2.3 Data Source

The literature search was conducted on three databases: Medline, Psych Info and Web of Science. Forward and backward citation searches of included studies were conducted using Google Scholar and Medline. Additional data were requested from authors if their articles had missing information. The search was started on Jan 10th, 2022 and ended on Nov 30th, 2023.

### 2.4 Searching Strategies

Keywords (e.g., schizophrenia, bipolar) as well as their variants (e.g., psychosis, manic depressive illness) were used to screen the title and abstract of the literature; see Tables S1–S4 for details.

### 2.5 Screening Procedure

**FIGURE 1:**
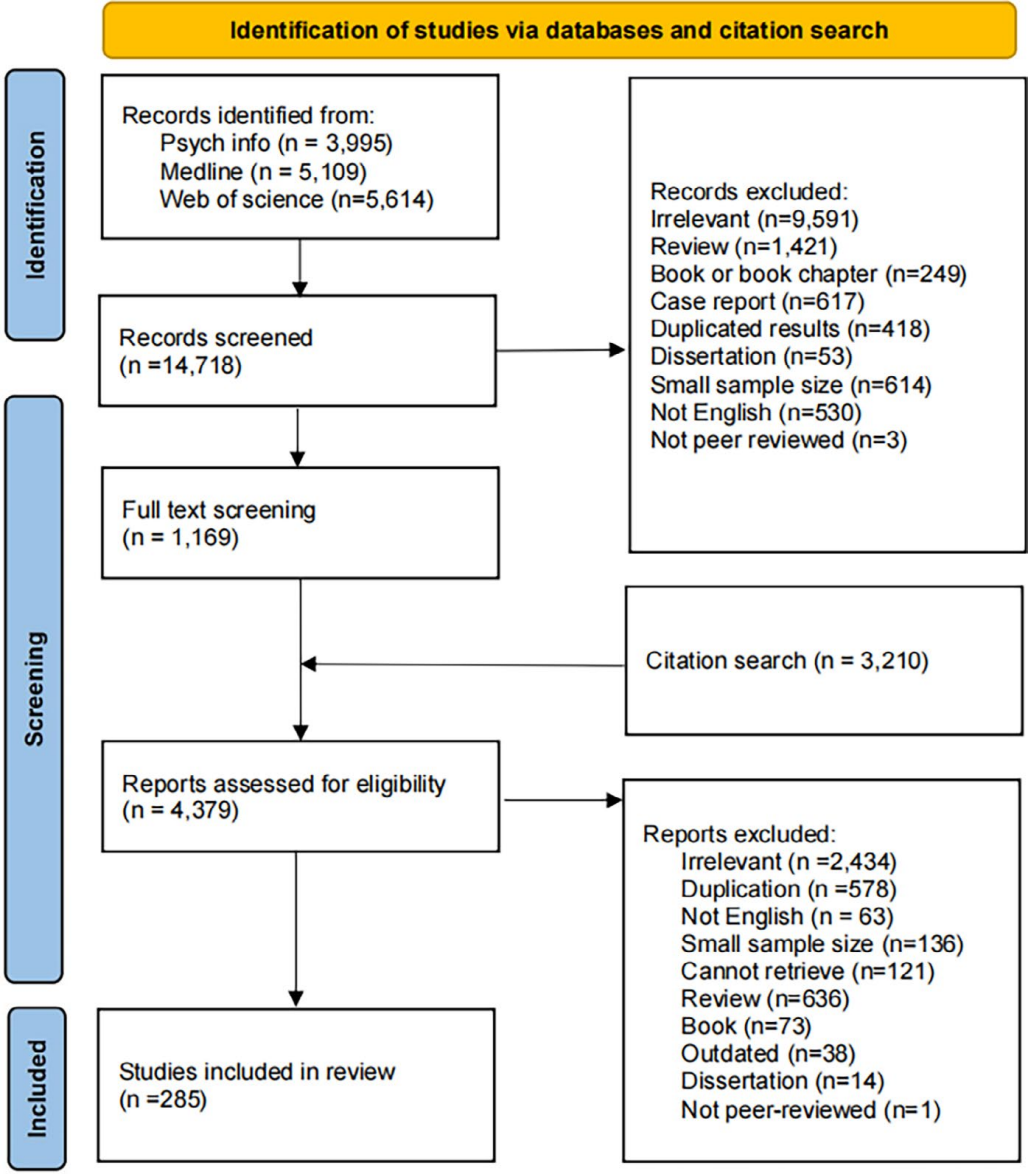
Flow diagram of the systematic review and meta‐analyses.

The three databases were searched by two independent reviewers and the flow diagram for the search is shown in Figure 1. First, title and abstract screening were conducted. There were 14,718 records identified. 13,549 records did not meet the inclusion criteria and thus were excluded, leaving 1169 records for full text screening. Based on the included studies, backward and forward citation search returned an additional 3210 results. Additionally, 95 enquiries were sent for studies with incomplete data specified in the eligibility criteria, and eight authors replied with data summaries. There were 285 records included in the current review; please see Tables 1 and 2 for further detail. A list of studies included in each analysis can be found in Tables S8–S12.

**TABLE 1:** Demographic and clinical information for studies of comorbid schizophrenia and psychosis.

	Schizophrenia	Psychosis
*N*	206,750	337,993
*K*	27	208
Sub synthesis (*k*)	Not applicable	Lifetime (110); Current (108); Bipolar I lifetime (40) and current (52); Bipolar II lifetime (13) and current (6); manic episode lifetime (9) and current (44); depressive episode lifetime (7) and current (22); mixed episode lifetime (4) and current (8); first episode (15); mood incongruent (21)
Age, M (SD)^a^	41.21 (10.30)	40.70 (6.85)
Female, *n* (%)^b^	127,201 (61.52)	120,062 (35.52)
Diagnostic standards (*k*)^c^	DSM (17), ICD (8), MDQ, RDC and mixed	DSM (167), ICD (25), mixed (6), RDC (5), unclear (3), DCR10 and DCUPR
Assessment tools (*k*)^c^	Unclear (14), SCID (7), BPRS, DIS, MDQ, OPCRIT, PSE, SAPS, SCAN	SCID (91), unclear (48), MINI (18), mixed (9), DIGS (8), SCAN (7), SADS (6), CIDI (3), AMDP (2), CGI (2), OPCRIT (2), PANSS (2), SID (2), BISS, CORE, DI‐PAD, DIS, ORSM, PDI, SAPS, YMRS
Study location (*k*)^d^	USA (9), AU (2), CN (2), EGY (2), IN (2), TR (2), UK (2), CA, CH, DK, ES, GL, QA, SE	USA (61), IT (20), TR (12), UK (11), FR (10), CN (8), IN (7), KR (7), BR (6), NO (6), CA (5), CH (5), DE (5), ES (5), EU (4), GL (4), AU (3), NL (3), PL (3), SE (3), DK (2), EGY (2), ET (2), AT, CL, FI, GR, HU, IE, IR, JP, LB, ME, MY, SG, TN, UG
Patient type (*k*)	Inpatient (6), Mixed (17), Outpatient (5)	Inpatient (34), Mixed (46), Outpatient (57), Unknown (51)
Bipolar type (*k*)	Psychotic Bipolar (4), Bipolar I (9) and BSD (15)	Psychotic Bipolar, Bipolar I (68), BSD (136) and Bipolar II (3)
Type of psychosis (*k*)	First rank symptoms (10), Schizophrenia (8), Psychotic disorders (7),^e^ Schizoaffective disorder (3)	Not applicable

*Note:* The study number is one for those without brackets.

^a^ Data was missing in 22 studies for psychosis, and seven studies for schizophrenia.

^b^ Data was missing in 11 studies.

^c^ The full name of the diagnostic standards and assessment tools can be retrieved from Supporting Information.

^d^ The study location used 2 or 3 letters country code. The GL indicated that the study was conducted in multiple countries.

^e^ The psychotic disorders include one study focused on schizophrenia spectrum disorder.

**TABLE 2:** Demographic and clinical information for studies of specific psychotic symptoms.

	Delusions	Hallucinations	Thought disorders
*N*	43,507	44,865	5952
Age, M (SD)^a^	43.02 (7.42)	43.08 (7.50)	44.66 (7.84)
Female, *n* (%)^b^	26,051 (59.88)	26,608 (59.31)	2851 (47.90)
*K*	76	71	15
Sub synthesis (*k*)	Lifetime (34), Current (47)	Lifetime (35), Current (42)	Lifetime (8), Current (10)
Diagnostic standards (*k*)^c^	DSM (54), ICD (8), unclear (6), RDC (4), mixed (4), DCR10, DCUPR	DSM (52), ICD (7), unclear (5), RDC (4) mixed (2), DCR10, DCUPR	DSM (9), RDC (3), DCUPR, ICD, mixed, unclear
Assessment tools (*k*)^c^	SCID (16), unclear (16), DIGS (6), mixed (6), SADS (4), OPCRIT (3), SAPS (3), AMDP (2), BPRS (2), DIP (2), MINI (2), PDI (2), PSE (2), SCAN (2), BISS, CAPE, CGI, CIDI, DI‐PAD, DIS, WGSQ, MDS	SCID (19), unclear (11), DIGS (6), mixed (5), OPCRIT (4), MINI (3), SADS (3), AMDP (2), DIP (2), SAPS (2), SCAN (2), BISS, BPRS, CGI, CIDI, CORE, DI‐PAD, DIS, EMA, HoNOS, MDS, PDI, PSE	SCID (3), mixed (2), OPCRIT (2), SADS (2) unclear (2), CMBT, DIGS, DIP, DI‐PAD, SAPS
Study location (*k*)^d^	USA (23), IT (10), DE (7), IN (6), UK (6), AU (4), CH (3), FR (3), KR (3), TR (2), CN, EGY, ET, EU, FI, GL, NL, PL, SE, UG	USA (27), UK (8), IT (7), DE (6), AU (4), IN (4), CH (3), KR (3), FR (2), NO, CN, EGY, ET, NL, SE, TR, UG	USA (11), UK (2), DE (2), AU
Patient type (*k*)	Inpatient (25), mixed (16), Outpatient (9), unclear (27)	Inpatient (21), mixed (21), Outpatient (11), unclear (19)	Inpatient (5), mixed (6), Outpatient (2), unclear (3)
Bipolar type (*k*)	Psychotic (13), Type I (27), BSD (37)	Psychotic (14), Type I (24), BSD (34)	Psychotic (2), Type I (9), BSD (5)
Type of conditions (*k*)	Any (47), Grandiosity (11), Persecutory (7), Paranoia (4), Reference (4) Suspiciousness (3), Perception	Any (46), Auditory (25), Visual	Any (6), formal thought disorder (3), positive formal thought disorder (3), disorganised speech (2), disorganised thoughts and behaviours

*Note:* The study number is one for those without brackets; BSD includes subtypes of bipolar I, II and not otherwise specified.

^a^ Missing values: delusions (*k* = 13), hallucinations (*k* = 15) and thought disorders (*k* = 1).

^b^ Missing values: delusions (*k* = 8), hallucinations (*k* = 7).

^c^ The full name of the diagnostic standards and assessment tools can be retrieved from Supporting Information.

^d^ The study location used 2 or 3 letters country code. The GL indicated that the study was conducted in multiple countries.

### 2.6 Coding

The coding for data extraction was conducted by two independent reviewers. An interrater reliability analysis was conducted with 25% of the papers randomly selected from all included studies. The overall kappa value for proposed variables in those studies was 0.89, which indicated excellent reliability. Any disagreements were resolved after discussion.

### 2.7 Variables

Independent variables such as demographic (e.g., gender), clinical (e.g., bipolar type) and methodological factors (e.g., publication date) were used to explore the potential sources of heterogeneities. The dependent variables were the prevalence of comorbid schizophrenia, psychosis (i.e., presence of hallucinations or delusions) and concurrent delusions, hallucinations or thought disorders, please see Table S5 for details. Information on diagnostic standards, assessment tools and locations was also collected and summarised in Tables 1 and 2.

### 2.8 Risk Assessment

The Joanna Briggs Institute (JBI) Critical Appraisal Checklist for prevalence studies was used to evaluate the quality of studies (see: https://jbi.global/critical‐appraisal‐tools). This checklist includes nine items that cover study sampling, reporting, analysis, measurement, procedure and outcome. For each item, reviewers rated the study using four categories: Yes (rated as 1), No (0), Unclear (0) and Not applicable. The procedure for the evaluation of the interrater reliability was identical to the coding schema; initial kappa was 0.62 and, in light of this, all discrepant ratings were examined, discussed and final ratings were agreed by consensus. Scores for included studies can be found in Tables S8–S12.

### 2.9 Statistical Analysis

All data was extracted manually except information contained in images, which was extracted by GetData Graph Digitizer software (see http://www.getdata‐graph‐digitizer.com/). Results of descriptive statistics will be presented to give a general account of the included studies, including demographic factors (e.g., age, gender, country) as well as clinical factors (e.g., subtype of BSD, duration of illness). We used single proportion meta‐analysis. To stabilise the variances between studies that were too high (i.e., 70%–100%) or low (i.e., 0%–30%), the double arcsine or Freeman‐Turkey transformation was performed [36]. Funnel plot and Egger's regression test for the asymmetry were conducted to detect publication bias [37]. A random effects model was used in the light of expected heterogeneity, estimated by the I statistic. Studentised residuals tests were applied to find outliers in the dataset. Subgroup analyses via meta regression were performed to see if any of the factors (e.g., research design, year of publication or demographic factors) could explain the variance between studies, and to identify meaningful associations between these factors and the primary outcomes. Meta analysis and regression were applied via the packages *meta* (version 6.5‐0) and *metafor* (version 4.4‐0); permutation test with 3000 iterations was carried out for regression models using the *dmetar* package (version 0.0.9000). R studio (version 1.4.1717) was used for all analyses.

## 3 Results

### 3.1 Comorbid Schizophrenia

Study details are summarised in Table 1, and the abbreviations as well as the full name of the diagnostic tools are listed in Table S6. The pooled prevalence of comorbid schizophrenia was 9.54% (95% CI: 5.68%–14.23%, *k* = 27). An outlier (*t* = 6.00) was detected, significantly influencing the pooled result [38]. The rate after the exclusion of the outlier was 8.04% (95% CI: 5.53%–10.96%, *k* = 26). Publication bias was not evident (*z* = 1.81, *p* = 0.07), and no significant effect of sample size was observed. Heterogeneity in this synthesis was very high (*I* ^2^ = 99.73%), suggesting the variance between studies is not negligible. Neither the time frame of the diagnosis (lifetime or current) nor the type of psychotic comorbidity affected the findings. Age and gender explained 23.01% of the between‐study variance, and the residual variance was reduced to 99.46%, implying that most of the heterogeneity is introduced by unknown variables. Being male (estimate = −0.004, SE = 0.001, *p* = 0.02) in the bipolar population was associated with risk of receiving a comorbid schizophrenia diagnosis, see Tables 3 and 4, Figures S1 and S2 for details.

**TABLE 3:** Pooled results of comorbid schizophrenia and psychosis in bipolar patients.

Analyses of comorbid conditions	Sub‐analyses	Pooled rate (95% CI)	𝛕^2^ (95% CI)	*I* ^2^ (95% CI)
Schizophrenia		8.04 (5.53–10.96)	0.01 (0.01–0.03)	99.7 (99.5–99.9)
Mood‐incongruent psychosis		47.43 (38.55–56.40)	0.04 (0.02–0.09)	95.9 (94.7–96.8)
Psychosis (lifetime)		53.08 (49.72–56.43)	0.03 (0.02–0.04)	97.6 (97.3–97.8)
	Bipolar I	62.81 (59.24–66.31)	0.01 (0.01–0.02)	87.9 (84.4–90.6)
	Bipolar II	16.97 (11.75–22.88)	0.02 (0.01–0.05)	88.4 (82–92.5)
	Manic episode	69.58 (57.15–80.75)	0.03 (0.01–0.14)	91.1 (84.8–94.8)
	Depressive episode	43.47 (29.72–57.74)	0.03 (0.01–0.19)	95.3 (92.1–97.2)
	Mixed episode	52.70 (22–67.71)^a^		
Psychosis (current)^b^		41.46 (36.88–46.11)	0.05 (0.05–0.08)	99.77 (99.70–99.82)
	Bipolar I	48.41 (41.82–55.03)	0.06 (0.04–0.09)	98.5 (98.4–98.7)
	Bipolar II	7.43 (5.73–9.33)	< 0.0001 (0–0.08)	47.6 (0–80.8)
	Manic episode^b^	51.5 (44.26–58.72)	0.06 (0.04–0.09)	98.6 (98.4–98.8)
	Depressive episode^b^	18.15 (12.46–24.63)	0.03 (0.02–0.07)	97.2 (96.6–97.8)
	Mixed episode	50.29 (23.09–77.40)	0.16 (0.07–0.68)	98.8 (98.4–99.1)
	First episode^b^	56.95 (44.31–69.15)	0.06 (0.03–0.15)	97.4 (96.6–98)

*Note:* The pooled rate and the I statistics presented in proportion; all pooled rates were based on a random effects model due to high heterogeneity.

^a^ The sample size for lifetime psychosis in patients with mixed episode was too low (*k* = 4) and thus the values reported were median and the range.

^b^ Marks the presence of possible publication bias.

**TABLE 4:** The information of predictors contributed to the heterogeneity.

Syntheses	Predictors	Estimate (SE)	*Q*	*R* ^2^	*Tau* ^2^	*I* ^2^
Schizophrenia	Gender	−0.004 (0.001)*	6.82*	0.23	0.01 (0.01–0.03)	99.46 (98.94–99.79)
	age	−0.002 (0.003)				
Mood‐incongruent psychosis	Publication date	−0.01 (0.004)**	12.94**	0.46	0.02 (0.01–0.04)	95.14 (90.65–98.02)
	Age	0.01 (0.005)**				
Lifetime psychosis	Gender	−0.003 (0.001)*	21.45***	0.15	0.02 (0.02–0.03)	96.97 (96.05–97.80)
	Bipolar type	0.14 (0.04)***				
LP‐bipolar I	Psychosis type	0.13 (0.06)*	15.32*	0.32	0.01 (0.003–0.01)	80.54 (66.88–89.90)
	First level evidence	0.09 (0.04)*				
LP‐Bipolar II	Age	0.01 (0.004)**	16.32**	0.64	0.01 (0.002–0.02)	79.09 (52.37–93.14)
	Gender	−0.01 (0.004)*				
LP‐depressive	Bipolar type	0.32 (0.07)·	21.58 ·	0.85	0.005 (0.001–0.04)	72.96 (28.78–96.16)
Current psychosis	Bipolar type	0.13 (0.05)**	31.14***	0.24	0.05 (0.04–0.07)	99.24 (98.99–99.45)
	Age	−0.01 (0.003)**				
	Patient type	0.07 (0.03)*				
CP‐bipolar I	Rate of mania	0.004 (0.001)***	24.68***	0.36	0.04 (0.02–0.06)	97.55 (96.34–98.56)
	Multicentre study	−0.17 (0.07)*				
	Clinical status	0.09 (0.07)				
CP‐manic	Rate of mania	0.003 (0.002)*	5.48	0.07	0.05 (0.03–0.10)	98.95 (98.37–99.41)
	Gender	−0.001 (0.007)				
	Age	0.005 (0.005)				
CP‐depressive	Gender	−0.01 (0.007)	11.73**	0.40	0.02 (0.01–0.06)	97.63 (95.45–99.09)
	Age	−0.01 (0.006)*				
CP‐mixed	Patient type	0.49 (0.30)	2.68	0.19	0.13 (0.05–0.65)	98.59 (96.55–99.71)
CP‐first episode	Rate of mania	0.006 (0.002)**	16.38**	0.56	0.03 (0.01–0.08)	96.48 (92.91–98.76)

*Note:* ·*p* > 0.05 and *p* < 0.1; *p < 0.05; **p < 0.01, ***p < 0.001; No meaningful predictors were found for lifetime psychosis in patients with manic episode or current psychosis in bipolar II patients; for regression on psychosis in mania, patients with mixed episode have been included since not all patients were suffering from unique manic episode.

Abbreviations: CP, current psychosis; LP, lifetime psychosis.

### 3.2 Comorbid Psychosis

#### 3.2.1 Mood Incongruent Psychosis (MIP)

The pooled prevalence of MIP in psychotic bipolar patients was 47.43% (95% CI: 38.55%–56.40%, *k* = 21), with a very high heterogeneity (*I* ^2^ = 95.9%). Publication bias was not evident (*z* = −0.50, *p* = 0.61). No significant outliers were identified, and subgroup analysis on the timeframe of the prevalence as well as the type of bipolar disorder did not yield significant findings. Meta‐regression found that age and publication date explained 45.67% of the between‐ study variance, and the heterogeneity still remained high (*I* ^2^ = 95.15%). Being older (estimate = 0.01, SE = 0.005, *p* = 0.01) predicted a higher prevalence of MIP; a similar trend was found for earlier date of publication (estimate = −0.01, SE = 0.004, *p* < 0.01).

#### 3.2.2 Lifetime Psychosis

The combined prevalence of lifetime psychosis in bipolar patients was 53.08% (95% CI = 49.72%–56.43%). Publication bias was not evident (*z* = 0.33, *p* = 0.74). Significant outliers were not detected. The between‐study variance estimated by *I* ^2^ is 97.74%, reflecting a high level of heterogeneity between studies. Subgroup analysis was conducted for this reason. Despite the evident difference between BSD types, the overall heterogeneity was not significantly reduced. Findings from meta‐ regression revealed that BSD type and gender (*k*_missing_ = 6) explained 16.45% of the total variance, and a minor decrease was observed in heterogeneity (*I* ^2^ = 96.97%). Patients with bipolar I (estimate = 0.14, SE = 0.04, *p* < 0.001) and who were male (estimate = −0.003, SE = 0.001, *p* < 0.05) were at increased risk of lifetime psychosis, see Tables 3 and 4, Figures S3–S6 for details.

Subsequent syntheses were conducted for lifetime psychosis in patients with bipolar I (*k* = 40) or bipolar II disorder (*k* = 13). The pooled lifetime prevalence of psychosis in patients with bipolar I disorder was 64.19% (95% CI: 59.64%–68.62%), and *I* ^2^ was 94.69%. Studentised residuals as well as sensitivity analysis suggested that a potential outlier (*t* = 5.16) caused a significant increase in the heterogeneity [39]; the heterogeneity was decreased to 90.86% after dropping this study. The pooled prevalence after the removal was 62.81% (95% CI: 59.24%–66.31%). Publication bias was not evident (*z* = −1.05, *p* = 0.30). Subgroup analysis on clinical status and patient source did not yield significant decreases in heterogeneity. Findings from meta‐ regression suggested that age of onset (*k*_missing_ = 7), first level evidence (studies with the highest quality scores), duration of illness, gender, age (*k*_missing_ = 3), patient type and publication date explained 32.16% of the variance, the residual *I* ^2^ was 80.55%. Prevalence of lifetime psychosis was higher in studies that used consecutive sampling (estimate = 0.09, SE = 0.04, *p* < 0.05) and lower for studies that reported first episode psychosis (estimate = 0.13, SE = 0.06, *p* < 0.02).

The synthesised rate of lifetime psychosis in bipolar II disorder was 16.97% (95% CI: 11.75%–22.88%), with high heterogeneity (*I* ^2^ = 88.4%) between studies. No significant outliers were identified. Subgroup analysis on clinical status as well as patient type did not produce significant findings. Meta‐ regression revealed that age (*k*_missing_ = 1) and gender explained 63.72% of the variance, and a change in heterogeneity was observed (*I* ^2^ = 79.10%). Bipolar II patients who were older (estimate = 0.01, SE = 0.004, *p* < 0.005) or male (estimate = −0.01, SE = 0.005, *p* < 0.02) were at more risk of lifetime psychosis.

The pooled rate of lifetime psychosis in BSD patients with a manic episode was 75.28% (95% CI: 59.67%–88.18%, *k* = 9), with a very high heterogeneity (*I* ^2^ = 95.58%). An outlier (*t* = 2.84) contributed significantly to the heterogeneity. The heterogeneity decreased to 91.20% after the removal of this study, and the pooled rate decreased to 69.58% (95% CI: 57.15%–80.75%, *k* = 8). Publication bias was not evident (*z* = 0.29, *p* = 0.77). Subgroup analysis on the nature of the mood episode did not explain any variance. No predictors were found in meta regression.

The pooled rate of lifetime psychosis for depression was 37.03% (95% CI: 21.14%–54.50%, *k* = 7), and the heterogeneity was very high (*I* ^2^ = 97.66%). An outlier (*t* = −2.39) significantly contributed to the heterogeneity; the heterogeneity after the removal was 94.65% and the rate was 43.47% (95% CI: 29.72%–57.74%). Publication bias was not evident (*z* = 0.65, *p* = 0.51). Subgroup analysis by BSD type explained 84.69% of the variance, and the remaining heterogeneity was high (*I* ^2^ = 72.96%). The nature of the mood episode did not reveal any difference between studies. After permutation, bipolar I was correlated with a higher prevalence of psychosis at a marginally significant level (estimate = 0.32, SE = 0.07, *p* = 0.07), see Tables 3 and 4, Figures S7–S14 for details.

Only four studies reported lifetime psychosis in patients with mixed episodes and thus we report a narrative review. The median prevalence was 52.70%, with a range between 22.00% and 67.71%. Mood episode in all of the studies was defined according to the DSM system. Three studies recruited patients from outpatient units and one recruited from an inpatient unit, but no distinctive pattern was observed. The lowest prevalence was observed in BSD patients (22%), and bipolar I patients had generally higher prevalences; in addition, the prevalence was lower in more recently published studies. However, caution should be taken when interpreting these findings due to the small number of studies, see Table S7 for details.

#### 3.2.3 Current or Recent Psychosis

The pooled rate of current or recent psychosis was 41.46% (95% CI: 36.88%–46.11%). Publication bias was significant (*z* = 3.57, *p* < 0.001). Heterogeneity was very high (*I* ^2^ = 99.50%). No significant outlier was detected. Subgroup analysis revealed that both bipolar type and patient type explained the between‐group variance. However, the remaining heterogeneity was still high (*I* ^2^ = 99.71% and 99.70%, respectively). Meta‐ regression found that the patient type, bipolar type and age explained 22.85% of the variance; a minor change in heterogeneity was observed (*I* ^2^ = 98.96%). Higher prevalence of psychosis was identified in bipolar patients who were recruited from inpatient units (estimate = 0.07, SE = 0.03, *p* < 0.05) and diagnosed with bipolar I disorder (estimate = 0.14, SE = 0.05, *p* < 0.005) as well as in younger patients (estimate = −0.01, SE = 0.003, *p* < 0.01).

The rate in bipolar I patients was 48.41% (95% CI: 41.82%–55.03%, *k* = 52) with a very high level of heterogeneity (*I* ^2^ = 98.50%). Publication bias was not evident (*z* = 1.65, *p* = 0.10). No outlier affecting the pooled prevalence was detected. Subgroup analysis did not find any variance that was contributed by patient type or clinical status. Meta‐ regression found that rate of mania, multicentre study and clinical status explained 35.82% of the variance. This did not lead to a significant reduction in heterogeneity, however (*I* ^2^ = 97.56%). Bipolar I patients who were experiencing manic episodes were at higher risk of recent psychosis (estimate = 0.004, SE = 0.0009, *p* < 0.001); studies with samples recruited from multicentre sites (estimate = −0.17, SE = 0.07, *p* < 0.02) reported a lower prevalence of psychosis. The prevalence in patients with bipolar II disorder was 9.20% (95% CI: 3.33%–17.39%, *k* = 6), with high heterogeneity (*I* ^2^ = 92.94%). A significant outlier (*t* = 7.08) was detected [40]. The rate of current psychosis in bipolar II after removal was 7.43% (95% CI: 5.73%–9.33%, *k* = 5) and the heterogeneity decreased to a moderate level (*I* ^2^ = 47.6%). Publication bias was not evident in this synthesis (*z* = −1.59, *p* = 0.11). No significant predictors were found in meta‐ regression.

The combined rate of current psychosis for mania was 51.50% (95% CI: 44.26%–58.72%, *k* = 44), with a very high heterogeneity (*I* ^2^ = 99.10%). Publication bias was evident (*z* = 2.00, *p* = 0.05). No significant outlier was found. Subgroup analysis did not find any variance contributed by the nature of mood episode or whether mixed or manic patients were involved. The rate of mania (*k*_missing_ = 2), gender (*k*_missing_ = 3) and age (*k*_missing_ = 4) explained 7.29% of the between study variance; the remaining heterogeneity, however, was still significant (*I* ^2^ = 98.95%), and a higher rate of mania predicted a higher prevalence of psychosis (estimate = 0.003, SE = 0.002, *p* < 0.05).

The rate of recent psychosis in patients with a depressive episode was 20.26% (95% CI: 13.36%–28.16%, *k* = 22). The heterogeneity was very high (*I* ^2^ = 99.30%). An outlier (*t* = 3.18) was identified [41]. The prevalence after the removal decreased to 18.15% (95% CI: 12.16%–24.63%, *k* = 21), and a minor change in heterogeneity was observed (*I* ^2^ = 99.05%). Publication bias was evident (*z* = 2.46, *p* < 0.01). The nature of the mood episode explained 9.19% of the between‐ study variance, and the remaining heterogeneity was 98.98%; no significant predictors were found after permutation. Gender and age explained 39.55% of the variance, and the remaining heterogeneity was 97.63%. Younger depressive patients were at greater risk of current psychosis (estimate = −0.01, SE = 0.006, *p* < 0.05).

The rate of current psychosis in patients with a mixed episode was 50.29% (95% CI: 23.09%–77.40%, *k* = 8), and the heterogeneity was very high (*I* ^2^ = 98.8%). Publication bias was not significant (*z* = 1.48, *p* = 0.14). No significant outlier was detected. Subgroup analysis on BSD type did not reveal any significant variance; patient type contributed to 18.80% of the between‐ group variance, however, the prediction effect was not significant.

The combined rate of first episode psychosis was 56.95% (95% CI: 44.31%–69.15%, *k* = 15); the heterogeneity was very high (*I* ^2^ = 97.40%). Significant publication bias was detected (*z* = 2.26, *p* < 0.02). No outliers significantly contributed to the heterogeneity. Subgroup analysis on the type of bipolar disorder did not reveal significant findings. Rate of mania (*k*_missing_ = 1) explained 56.30% of the between‐ group variance; the remaining heterogeneity decreased to 96.48%. Bipolar patients with a manic episode were at a higher risk of experiencing psychosis in their first mood episode (estimate = 0.006, SE = 0.002, *p* < 0.002), see Tables 3 and 4, Figures S15–S28 for further reference.

### 3.3 Distributions of Specific Psychotic Symptoms in Bipolar Populations

#### 3.3.1 Cooccurring Delusions

The combined prevalence of lifetime delusions in bipolar patients was 55.11% (95% CI: 45.70%–64.34%, *k* = 34), with a very high heterogeneity (*I* ^2^ = 99.13%). Publication bias was not evident in the included studies (*z* = 1.06, *p* = 0.29) and no significant outliers were identified. Subgroup analysis revealed that the bipolar type explained 46.52% of the variance. Patients with more severe BSD (bipolar I or psychotic bipolar) were at increased risk of lifetime delusions (estimate = 0.27, SE = 0.05, *p* < 0.001). The prevalence of current delusions in this population was 47.19% (95% CI: 38.53%–56.94%, *k* = 47), with a very high heterogeneity (*I* ^2^ = 99.06%). Significant publication bias was detected by Egger's test (*z* = 2.72, *p* = 0.01). No significant outliers were detected. Subgroup analysis indicated that different bipolar types explained 23.71% of the variance, and the overall variance decreased to 98.58%. Meta‐ regression revealed that first level evidence and bipolar type explained 37.04% of the variance. The remaining heterogeneity was still high (*I* ^2^ = 98.23%). Studies recruited consecutive patients (estimate = 0.26, SE = 0.08, *p* < 0.01) or patients with more severe bipolar disorder (estimate = 0.15, SE = 0.05, *p* < 0.001) tended to report higher current delusions, see Tables 2, 5 and 6 as well as Figures S29–S32.

**TABLE 5:** Pooled results of cooccurring delusions, hallucinations and thought disorders in bipolar patients.

Analyses of comorbid conditions	Sub‐analyses	Pooled rate (95% CI)	𝛕^2^ (95% CI)	*I* ^2^ (95% CI)
Delusions	Lifetime	55.11 (45.70–64.34)	0.08 (0.05–0.13)	99.1 (98.7–99.5)
	Current^a^	47.19 (38.53–55.94)	0.09 (0.06–0.14)	99.1 (98.6–99.4)
Hallucinations	Lifetime	31.26 (26.01–36.76)	0.03 (0.02–0.05)	97.5 (96.0–98.6)
	Current	19.62 (14.37–25.43)	0.05 (0.03–0.08)	98.7 (98.0–99.2)
Thought disorders	Lifetime	29.94 (15.08–47.34)	0.07 (0.03–0.28)	99.2 (98.2–99.8)
	Current^a^	22.15 (6.70–42.99)	0.09 (0.04–0.42)	98.1 (97.4–98.7)

*Note:* The pooled rate and the I statistics were presented in proportions; all pooled rates were based on a random effects model due to high heterogeneity.

^a^ Denotes the presence of possible publication bias.

**TABLE 6:** The predictors contributing to the heterogeneity in analyses of specific psychotic symptoms.

Syntheses	Predictors	Estimate (SE)	*Q*	*R* ^2^	𝜏^2^	*I* ^2^
DelusionsL	Bipolar type	0.27 (0.05)***	28.32***	0.47	0.04 (0.03–0.07)	98.33 (97.40–99.07)
DelusionsC	Bipolar type	0.15 (0.05)***	27.65***	0.37	0.06 (0.04–0.09)	98.23 (97.37–98.91)
	First level evidence	0.26 (0.08)**				
HallucinationsL	Sampling method	0.30 (0.08)***	15.94**	0.34	0.02 (0.01–0.03)	96.24 (94.05–97.95)
HallucinationsC	Bipolar type	0.10 (0.04)**	6.72**	0.14	0.04 (0.03–0.07)	98.41 (97.58–9)
Thought disordersL	—	—	—	—	—	—
Thought disordersC	Age	−0.03 (0.01)***	13.68***	0.72	0.03 (0.009–0.26)	93.82 (81.98–99.25)
	Gender	−0.008 (0.004)*				

*Note:* ·*p* > 0.05 and *p* < 0.1; *p < 0.05; **p < 0.01, ***p < 0.001.

Abbreviations: C, current; L, lifetime.

#### 3.3.2 Cooccurring Hallucinations

The combined lifetime prevalence of hallucinations in bipolar patients was 32.59% (95% CI: 25.79%–39.77%, *k* = 35), with a very high heterogeneity (*I* ^2^ = 98.53%). One outlier had significant influence (*t* = 5.97) on the pooled result [42]. The new prevalence after excluding the outlier was 31.26% (95% CI: 26.01%–36.76%, *k* = 34), and the heterogeneity dropped to 97.48%. The publication bias was not significant (*z* = 0.92, *p* = 0.36). Neither types of hallucination nor types of bipolar disorder explained significant between‐group variance. Sampling method explained 33.86% of the variance. The remaining heterogeneity decreased to *I* ^2^ = 96.24%. Studies that applied probabilistic sampling (estimate = 0.30, SE = 0.08, *p* < 0.001) were associated with higher lifetime hallucinations.

The pooled rate of current hallucination was 19.62% (95% CI: 14.37%–25.43%, *k* = 42) with very high heterogeneity (98.71%). A significant outlier (*t* = 3.09) was detected; however, it has no significant impact on the pooled prevalence and heterogeneity, and thus the analysis continued without excluding it [43]. Publication bias was again not significant across studies (*z* = 1.47, *p* = 0.14). Subgroup analysis revealed that the bipolar type contributed 13.50% of the total variance, and the remaining heterogeneity was still substantial (*I* ^2^ = 98.41%). Patients with more severe bipolar disorder (estimate = 0.10, SE = 0.04, *p* < 0.01) were at risk of current hallucinations, see Tables 5 and 6, Figures S33–S36 for details.

#### 3.3.3 Cooccurring Thought Disorder (TD)

The synthesised prevalence of lifetime TD in bipolar patients was 29.94% (95% CI: 15.08%–47.34%, *k* = 8), with high heterogeneity (*I* ^2^ = 99.22%). No publication bias was detected (*z* = −0.73, *p* = 0.46). No significant outlier was found. Subgroup analysis revealed that different bipolar types did not contribute to the difference between studies. No significant predictors were observed in this analysis. The rate of current TD was 31.25% (95% CI: 11.50%–55.31%, *k* = 9), with a very high between‐ study variance (*I* ^2^ = 98.81%). An outlier (*t* = 2.62) was identified [44] and the rate fell to 22.15% after its removal (95% CI: 6.70%–42.99%); the heterogeneity reduced to *I* ^2^ = 98.18%. Publication bias was evident (*z* = 2.57, *p* = 0.01). Neither types of TD nor BSD explained the between‐ group variance. In meta regression, age and gender explained 71.65% of the variance; the remaining heterogeneity was still high (*I* ^2^ = 95.31%). Patients being male (estimate = −0.007, SE = 0.004, *p* < 0.05) or younger age (estimate = −0.03, SE = 0.009, *p* < 0.001) were at most risk of current thought disorder, see Tables 5 and 6, Figures S37–S40 for details.

## 4 Discussion

In the current study, we have synthesised findings on the prevalence in bipolar patients of comorbid schizophrenia, psychosis and the specific symptoms of delusions, hallucinations and thought disorder. Our findings are summarised in Tables 3 and 5, and here we draw readers' attention to the main observations. In summary, the low rate of comorbid schizophrenia in the bipolar population increased when a broader definition was applied (mood incongruent psychosis). Lifetime psychosis was higher than current or recent psychosis, and this was true for all the analyses. Patients with bipolar I or experiencing a manic episode had a much higher rate of comorbid psychosis than those with bipolar II or with depressive episodes. Delusions were more prevalent than thought disorder or hallucinations in bipolar patients. For context, the estimated prevalence of comorbid schizophrenia is considerably higher than its estimated prevalence (i.e., 0.4%–2%) in the general population [45], or even the prevalence of broadly defined psychosis (about 3%) [46]. In comparison to the previous attempts to synthesise these data by Aminoff, Onyeka, Ødegaard, Simonsen, Lagerberg, Andreassen, Romm and Melle [35], our approach includes more studies on psychosis and added the analyses of comorbid schizophrenia diagnoses as well as specific psychotic symptoms. The merits of our study compared to that of Chakrabarti and Singh [34] were the quantitative synthesis and heterogeneity analyses, as well as the synthesis on thought disorder in bipolar patients. In all analyses, a high level of heterogeneity was observed. In general, although some meaningful factors were identified in meta‐regression, this remained high in these further analyses, and we comment further about this in the research limitations.

In one sense, the findings are unsurprising. The high prevalence of psychosis in this population has been noted in numerous previous studies and, indeed, was the motivation for many of the studies included in this meta‐analysis. Our estimate that lifetime psychosis is present in approximately three out of five bipolar I patients and nearly a fifth of bipolar II patients is slightly lower than the values reported by the two previous systematic reviews [34, 35], and this seems to be mainly accounted for by the lower rate we observed in bipolar II patients. This difference could be explained by different search and inclusion strategies. We applied a relatively conservative approach to identifying papers and yet our estimate of the lifetime prevalence in bipolar patients was computed from a larger dataset (*k* = 110) than those in prior reviews. The prevalence of recent or current psychosis was also relatively lower than reported previously, with about two out of five patients affected.

The higher rate of psychosis in bipolar I versus bipolar II was expected and can be in part explained by the fact that the former is the more severe condition, but also by the fact that bipolar I criteria require a history of manic episodes whereas those for bipolar II do not. Hence, this finding is consistent with our observation that psychotic symptoms are more evident in mania than depression. This observation was further supported by subgroup analyses of bipolar I and bipolar II samples, where the estimated confidence intervals did not overlap. This finding is consistent with a prior review [35], and a network analysis, which showed stronger connections between positive and manic symptom clusters [27]. Findings from meta‐ regressions on the small groups were less consistent. The proportion of bipolar patients with a manic episode predicted current psychosis in the entire dataset and in some sub‐analyses, although the effect sizes were relatively small. For lifetime psychosis, the best model did not contain this variable due to missing values.

Male patients were more likely to be diagnosed with comorbid schizophrenia compared with female patients, which is consistent with a greater prevalence of schizophrenia in men, especially in early adulthood [47, 48]; similar findings were identified in the meta‐ regressions of lifetime psychosis in general bipolar and bipolar II patients, and the same effect was observed for current thought disorder. Moreover, previous research has suggested that male patients with bipolar disorder are more likely to have manic symptoms than female patients [49]. Hence, the gender differences observed are consistent with an overlap between bipolar disorder and schizophrenia conditions, or possibly common processes involved in bipolar I disorder with manic symptoms and schizophrenia.

As expected, and consistent with previous literature [50], we found that delusions are common in bipolar patients, with an estimated prevalence similar to that for lifetime psychosis, and with no evident difference between the prevalence of lifetime and current delusions. Our estimate of combined prevalence is lower than the mean or median prevalence of lifetime or current delusions reported earlier [34]. In meta‐ regression, bipolar type predicted the prevalence of lifetime and current delusions, with bipolar I or psychotic bipolar patients especially likely to report a history of delusions. This likely reflects the fact that delusions, especially of grandiosity, are a common feature of the manic syndrome [29, 51]. We found that hallucinations are less prevalent in bipolar patients; overall about three in 10 had a history of hallucinations and one fifth had current hallucinations, figures that are also comparable to those reported by Chakrabarti and Singh [34]. Finally, the approximate prevalence of thought disorder was similar across the different time frames, with around one quarter of bipolar patients suffering from lifetime thought disorder and one fifth suffering from current thought disorder. Although once thought pathognomonic for schizophrenia [52], it has been known for some time that this symptom is common in bipolar patients [53] and our observations are comparable to those in meta‐analyses focused on quantitative comparison between patients with schizophrenia and bipolar disorder [29, 54].

### 4.1 Limitations

The main limitation of this review is the high heterogeneity observed within the main and sub‐analyses, although some of the heterogeneities have been explained by factors such as bipolar type or the presence of mania. The remaining heterogeneities were generally high in all of the analyses. Some of this heterogeneity could have been introduced by the use of different diagnostic and assessment tools. However, our study size lacks power to detect such variance without risk of overfitting. It is also plausible that variance was introduced by different sampling techniques, research designs or other unknown factors. Another limitation is that study numbers for some subgroup analyses (e.g., psychosis in different mood episodes, thought disorder) were low. For example, only four studies have reported lifetime psychosis in bipolar patients with mixed episodes; therefore, a narrative review was conducted. Caution should be exercised when interpreting the findings from these analyses.

Additionally, the current findings did not quantitatively examine other clinical characteristics such as prognosis and treatment responses that may differ or be similar between BSD and schizophrenia. This study has focused on the psychopathological manifestations of schizophrenia in BSD, and other features such as course, outcome and treatment response were beyond its scope. For prognosis, there has been evidence that the major psychoses can be divided into several subgroups according to the illness trajectories [55]. In contrast to the pessimistic outlook of Kraepelin, however, schizophrenia is not always associated with poor prognosis, highlighting the necessity of a transdiagnostic approach to psychiatric classification. Meanwhile, pharmaceutical treatments are primarily neurotransmitter‐oriented, and responses to them can be influenced by various factors [56, 57] and even within a single diagnostic group, treatment response can vary markedly [58]. Consequently, determining the specificity of treatment effects based solely on categorical diagnoses is challenging, particularly when patients follow treatment plans that combine antipsychotics and mood stabilisers. In the only placebo‐controlled study we are aware of in which patients with severe mental illness were randomly assigned to either mood stabilisers, antipsychotics or both, treatment response was predicted by symptoms but not diagnoses [59]. These challenges underscore the need for treatments targeting common deficits across these disorders, such as biological markers or cognitive dysfunctions [60, 61]. For example, the Research Domain Criteria (RDoC) represent a significant shift in research approach, emphasising transdiagnostic psychological and neurobiological processes.

### 4.2 Implications

The present findings have implications for understanding the relationship between bipolar disorder and schizophrenia, the causal processes relating to both diagnoses, as well as the mechanisms involved in specific psychotic symptoms experienced by bipolar patients. The Kraepelinian distinction between bipolar disorder and schizophrenia was a cornerstone of psychiatry throughout the 20th century [62] but continues to be a source of controversy today. In an attempt to resolve this issue Kasanin [63] proposed the concept of schizoaffective psychosis, which was originally conceived as a separate condition combining symptoms of the other two conditions. The idea of a schizo‐bipolar spectrum was raised by later researchers [64] and has recently received support from analyses of large samples combining patients from across the psychosis spectrum [65]. However, some investigators have proposed more complex models, most notably hierarchical and bifactor models that assume a common psychosis disease process together with separate factors that explain the preponderance of mood versus psychotic symptoms [66, 67, 68]. These debates have led to an ongoing and concerted effort to create a new, more scientifically valid classification of psychiatric disorders [69]. Clearly the present findings point to the importance of this development and, yet, as a caveat, it should be pointed out that recent philosophical treatments of natural classification systems have argued that we may have to abandon the idea that one system can be devised that will be fit for all purposes (in the case of psychiatric conditions, these main purposes are epidemiology and public health surveillance, disease mechanism discovery and clinical prediction) [70]; the alternative is to adopt a ‘promiscuous realism’ which recognises that any system is likely to have limitations and may be better suited to some purposes rather than others.

Attempts to resolve issues of psychiatric taxonomy can, to some extent, be addressed by focusing on causal factors. In the introduction, we highlighted that a considerable literature exists pointing to common aetiological and disease mechanisms in psychosis and bipolar disorder, including shared genetic architecture [20], psychosocial risk factors such as childhood trauma [22, 23], and neurobiological processes [71]. Research in psychosis suggests that there may be some specificity for different kinds of social risk factors and specific symptoms [72] and it is possible that a similar kind of specificity will be found for some biological factors. This observation highlights the potential value of investigating mechanisms involved in the psychotic symptoms experienced by bipolar patients. Psychological research on delusions has placed considerable emphasis on emotional processes [73] but this work has mostly focused on paranoia, and grandiosity has been relatively neglected [74]. A few studies focusing on grandiosity in schizophrenia patients have emphasised the role of motivation to find purpose in life [75] and, in future studies, it might be fruitful to explore how this relates to reward sensitivity [76] and the high goal motivation [77] observed in bipolar patients. Very little is known about hallucinations in bipolar patients but, in patients diagnosed with schizophrenia, it is thought that they are the consequence of a failure of source monitoring mechanisms which allow individuals to distinguish between their own mental events and external stimuli [78]. Thought disorder is probably the only psychotic symptom in which detailed comparisons have been made between schizophrenia and bipolar patients. In both the former [79] and the latter [80] it has been shown that thought disorder is a speech production abnormality that is activated when high levels of emotion disrupt cognitive processing [81].

## 5 Conclusion

In conclusion, our findings point to considerable overlap between bipolar disorder and schizophrenia, both in terms of phenomenology and psychopathology. The idea that these are two separate conditions no longer seems tenable. Future research should explore comparisons between patients with the two diagnoses who experience the same symptoms and common symptom‐causing processes.

## Author Contributions

The first author and the corresponding author designed this study. The literature screening, study quality assessment, and variable coding were done by first and second authors. The first author was responsible for the statistical analyses and drafting the manuscript. Subsequent revisions were conducted by the first and corresponding authors.

## Funding

The authors have nothing to report.

## Conflicts of Interest

The authors declare no conflicts of interest.

## Supporting information


Figure S1–S40: Forest and funnel plots of pooled rates across syntheses.

**Table S1–S7** **: Search strategies, coding methods, abbreviations, and lifetime psychosis in bipolar patients (mixed episode).**

Table S8–S12: Summary tables of included studies.

## Data Availability

The data that support the findings of this study are available from the corresponding author upon reasonable request.
