# Supplementary material for: Comorbid Schizophrenia and Psychotic Symptoms in Patients With Bipolar Disorder: A Meta‐Analysis of the Global Literature
Source: Bipolar Disord. 2026 Mar 18;28(3):e70093. doi: 10.1111/bdi.70093 (PMC12997143; doi:10.1111/bdi.70093)
Supplement: Supplementary file 1 — Figure S1–S40: Forest and funnel plots of pooled rates across syntheses. [file BDI-28-0-s002.docx]

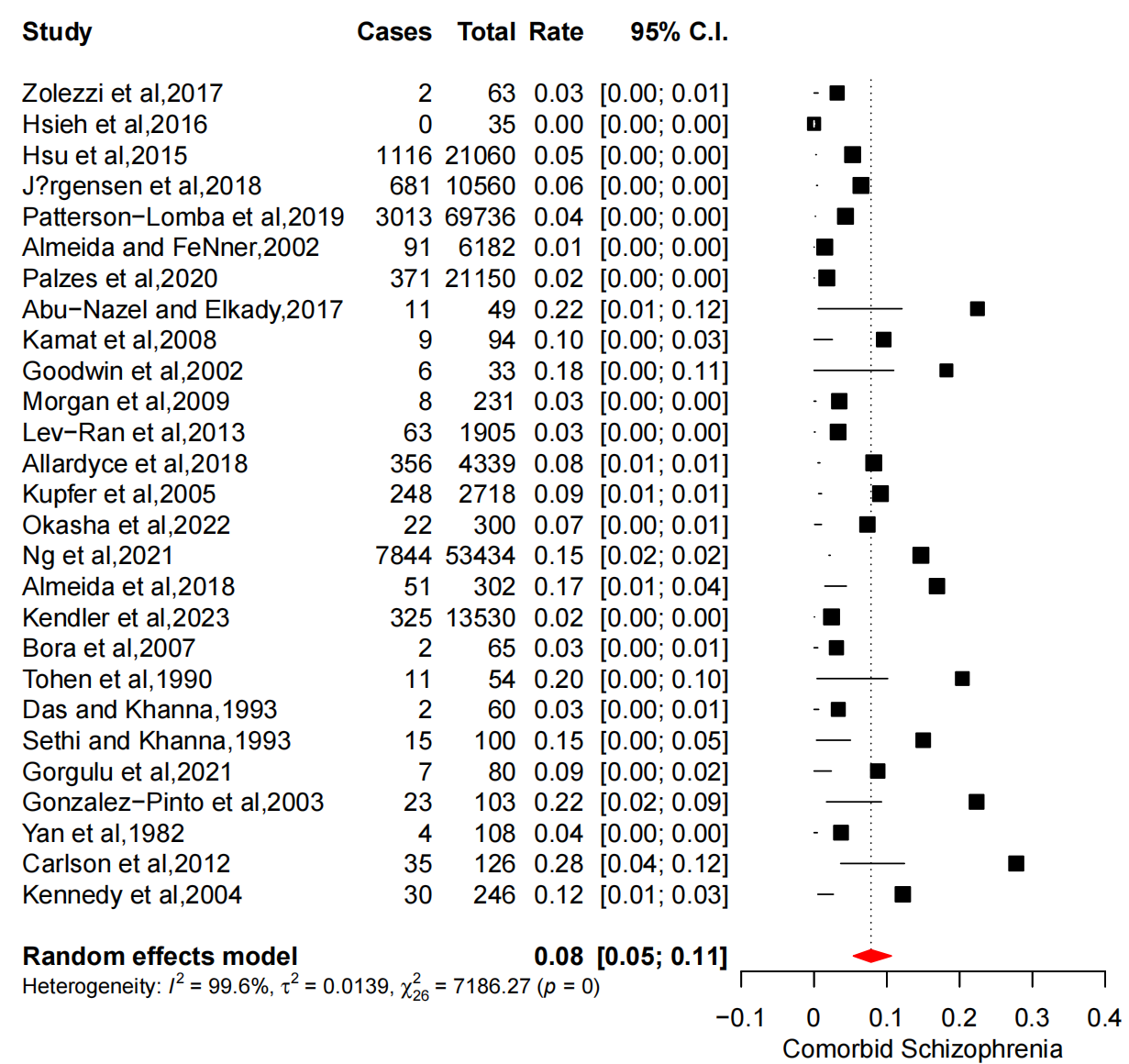


**Figure S1**

***Pooled Prevalence of Comorbid Schizophrenia in Bipolar Patients.***


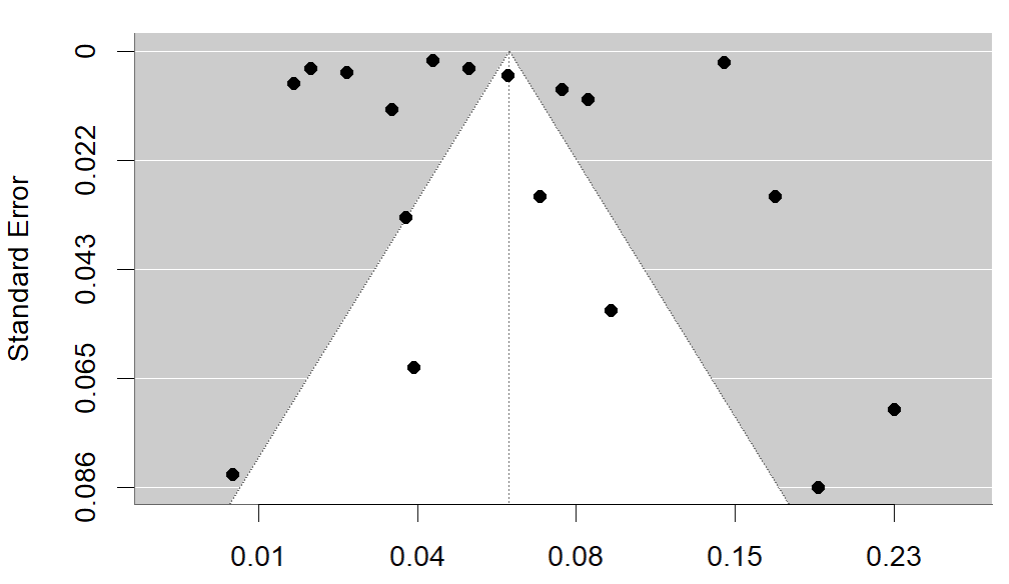

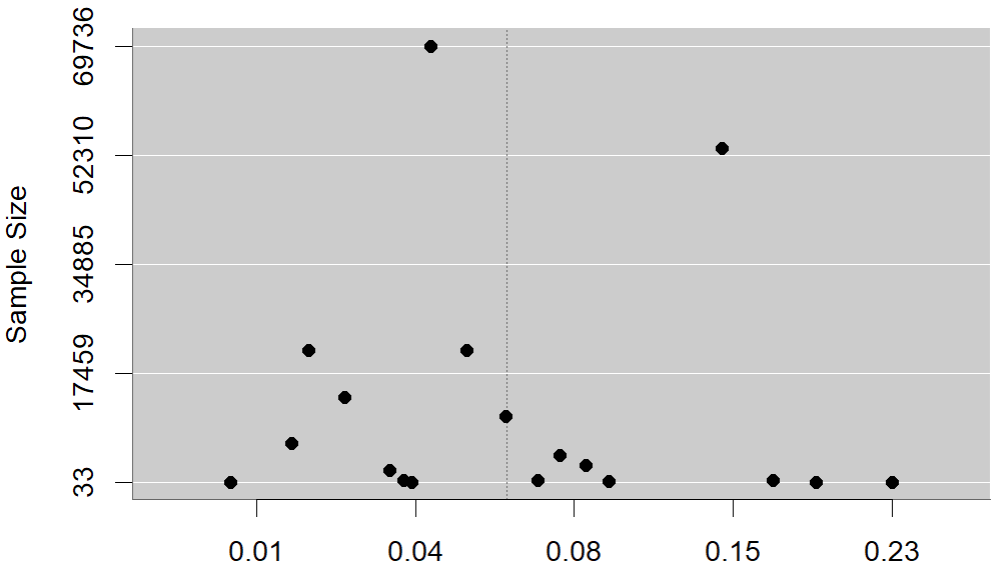


(a) original funnel (b) plot by sample size

**Figure S2**

***Funnel Plots for the Comorbid Schizophrenia***


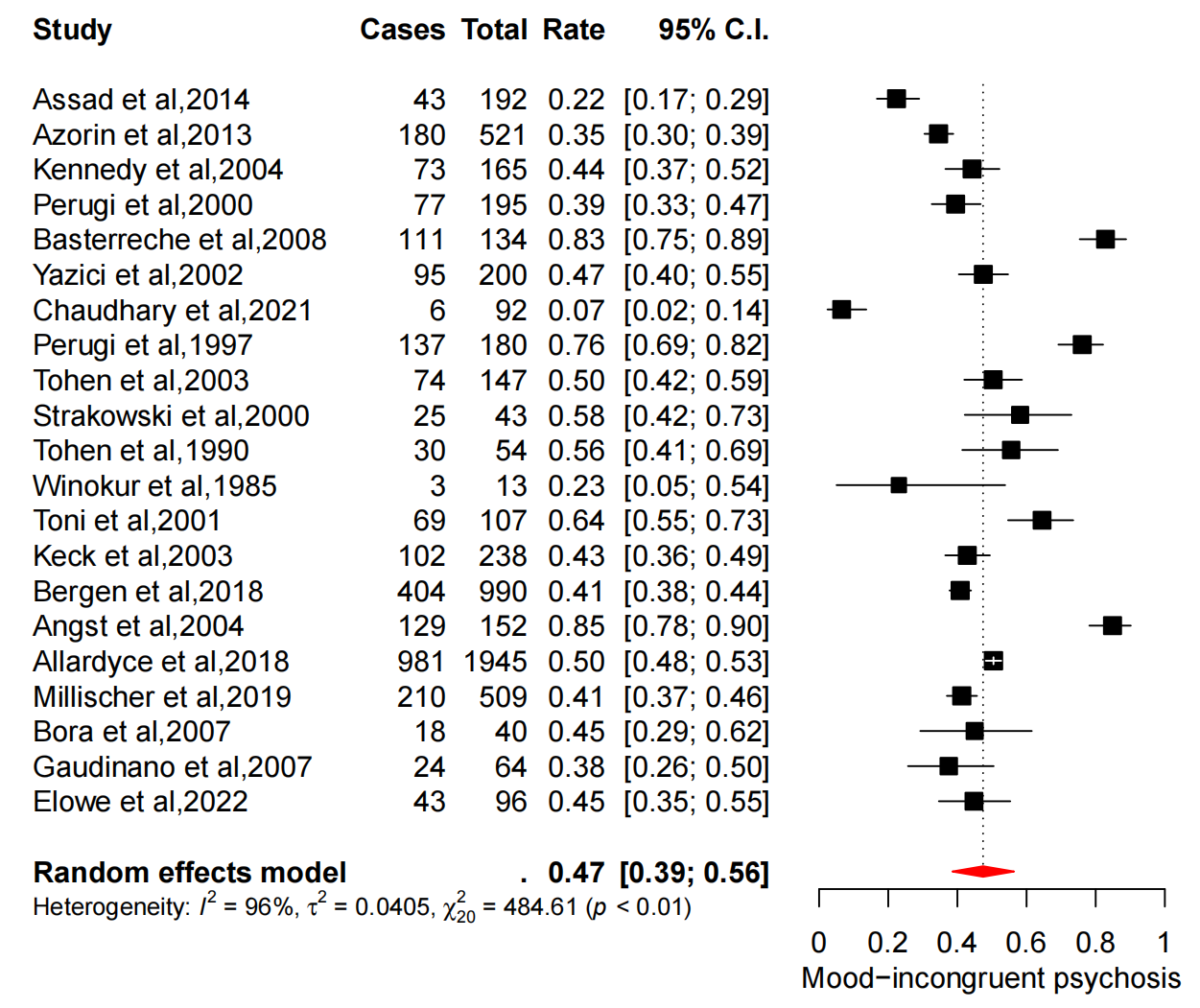


**Figure S3**

***Pooled Prevalence of Mood incongruent psychosis***


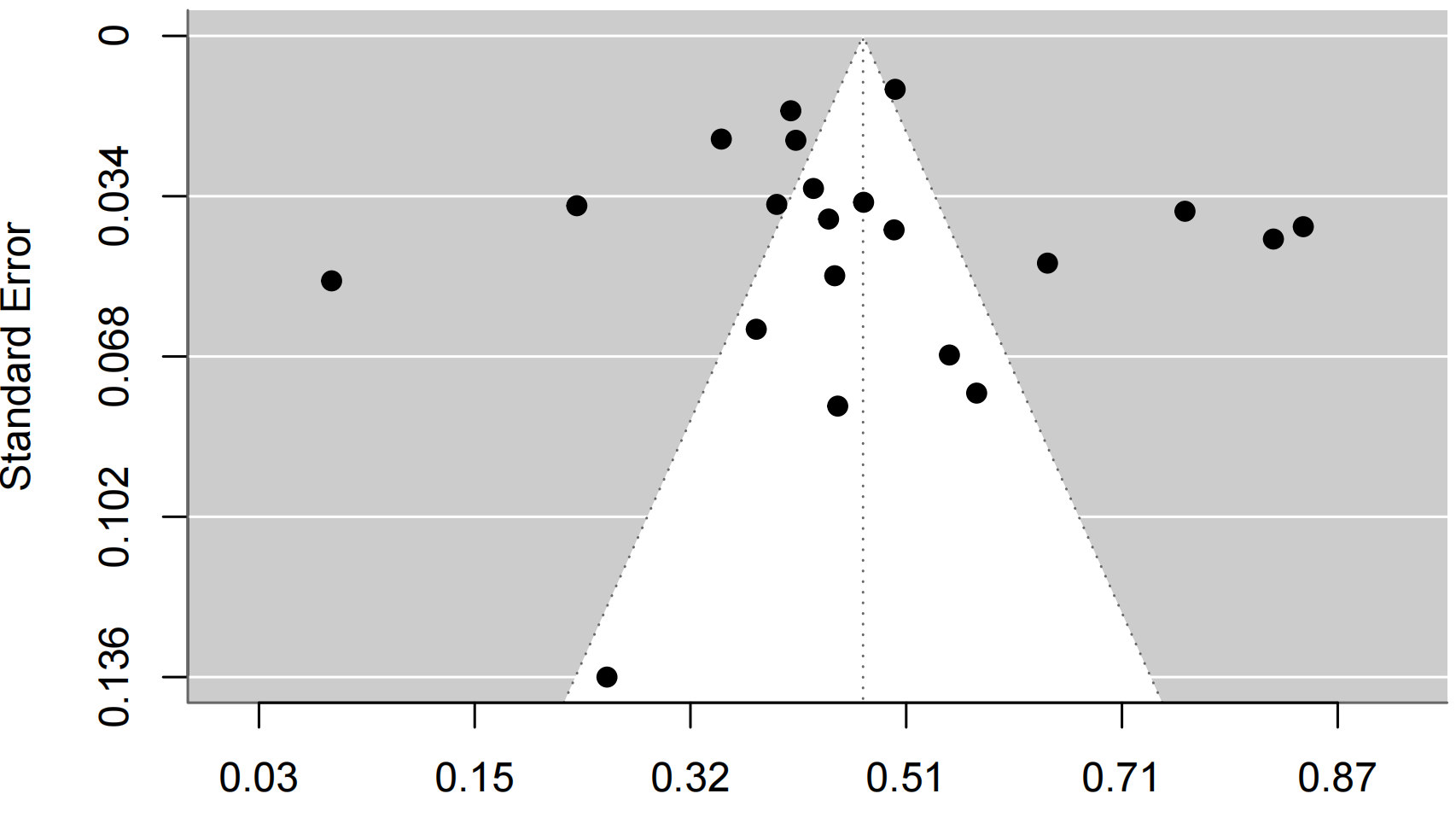

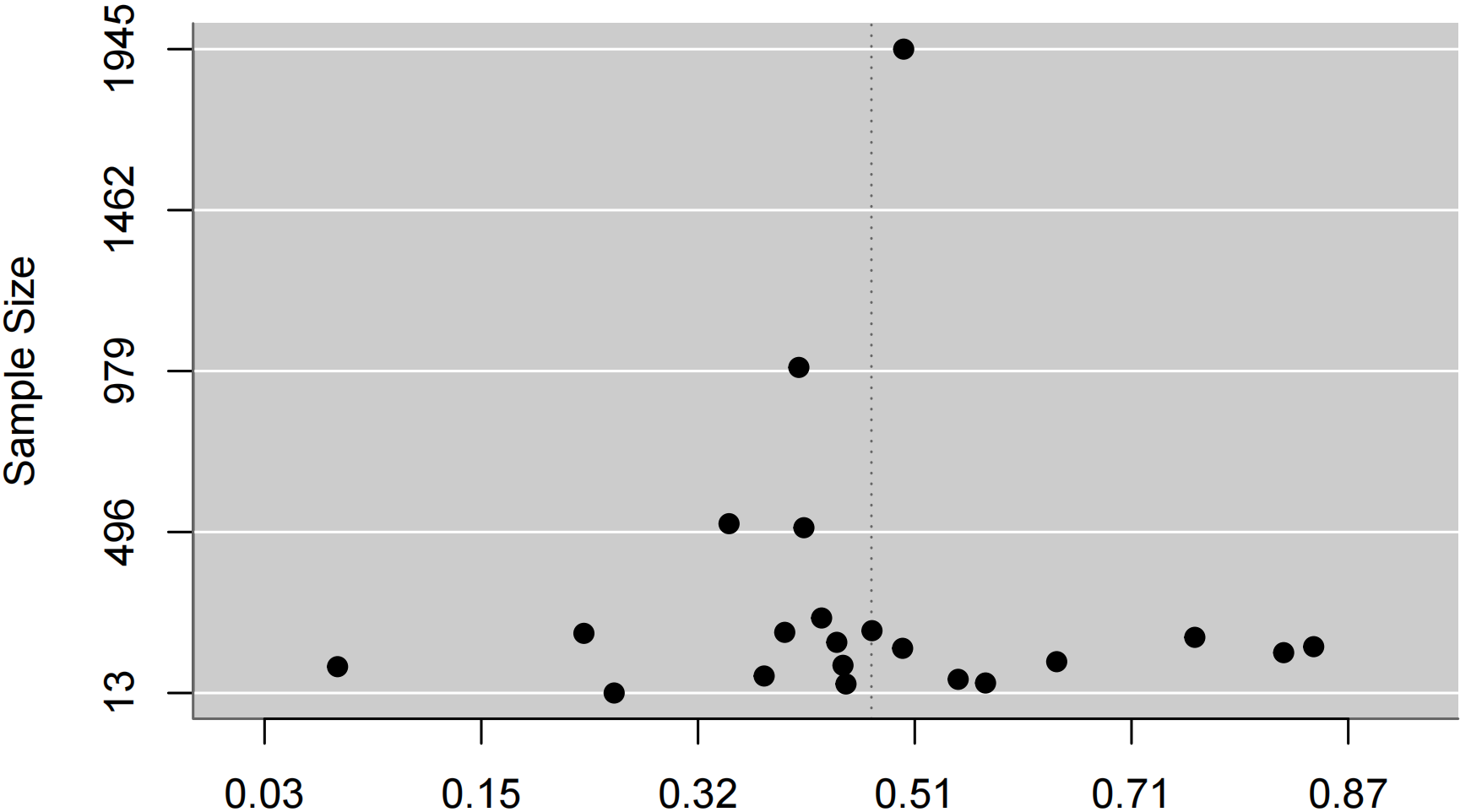


(a) original funnel (b) plot by sample size

**Figure S4**

***Funnel plots for mood incongruent psychosis***

***
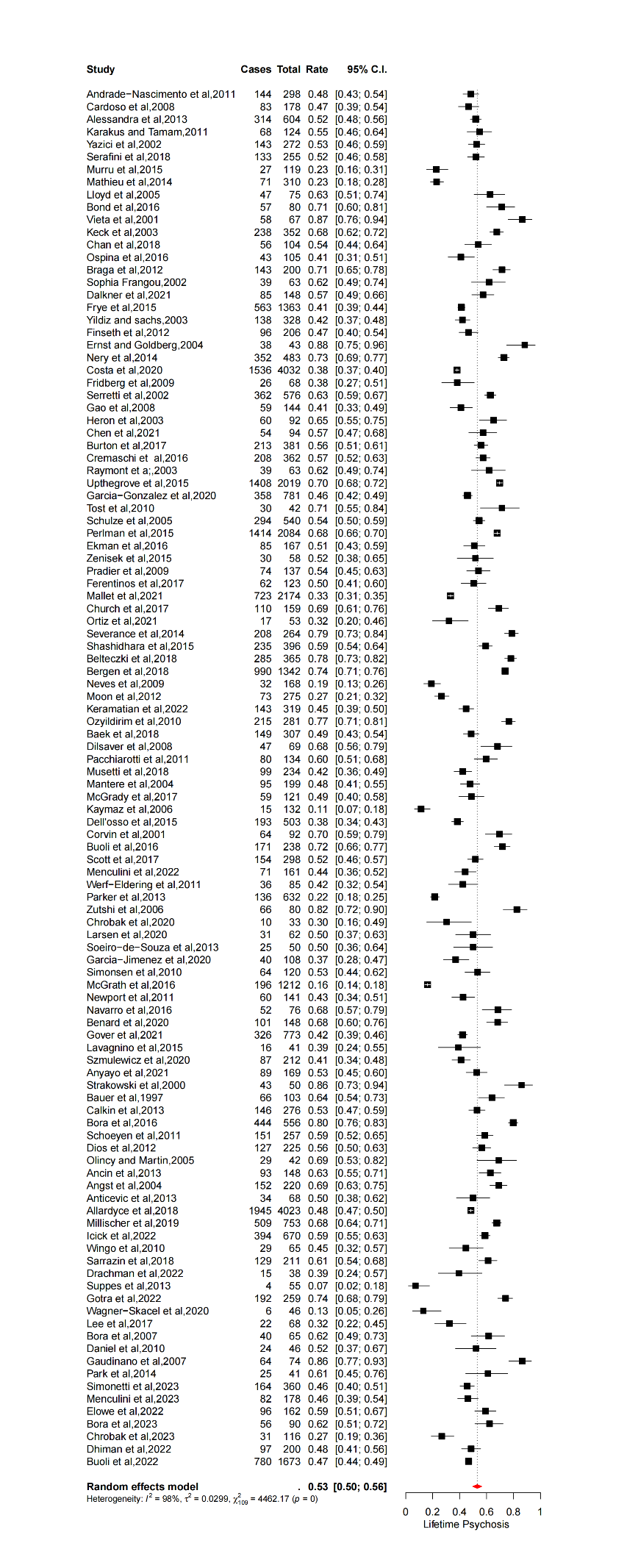
***

**Figure S5**

***Pooled rate of lifetime psychosis in bipolar patients***


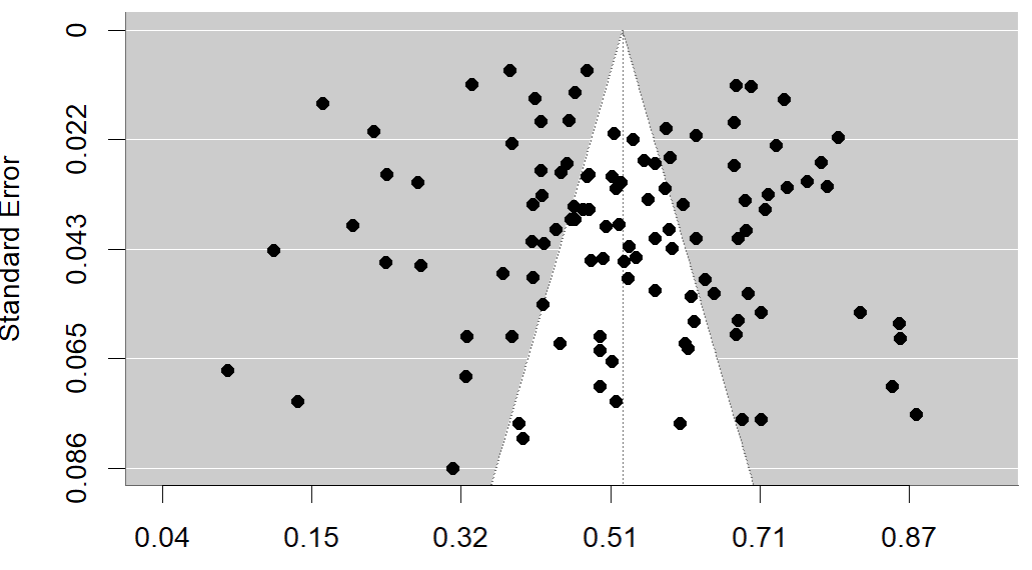

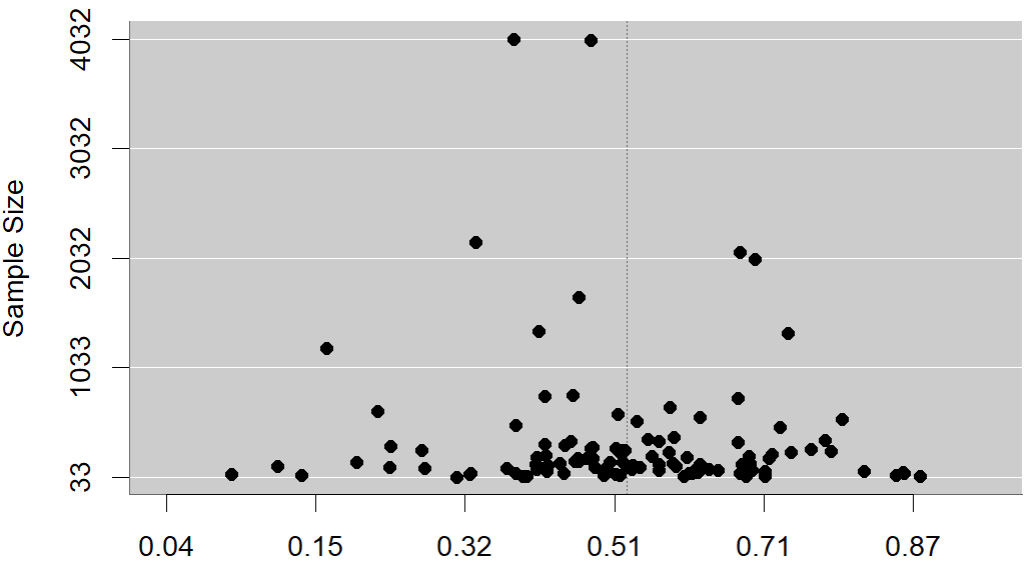


(a) original funnel (b) plot by sample size

***Figure S6***

***Funnel plots for the lifetime psychosis***


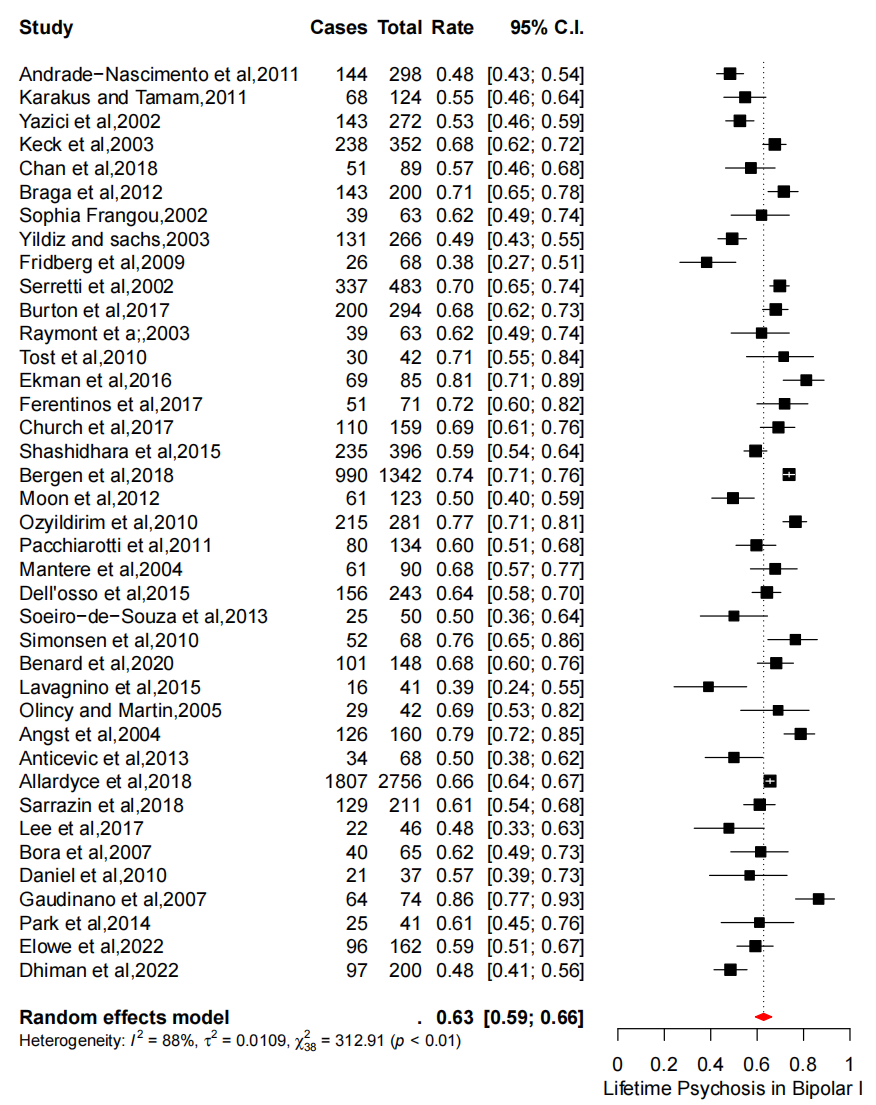


**Figure S7**

***The rate of lifetime psychosis in patients with bipolar I***


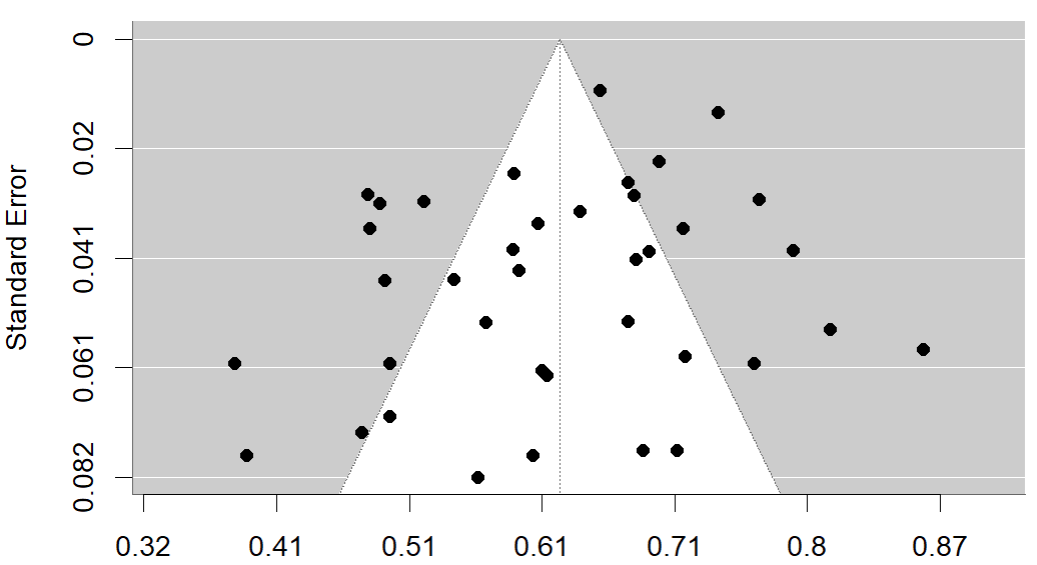

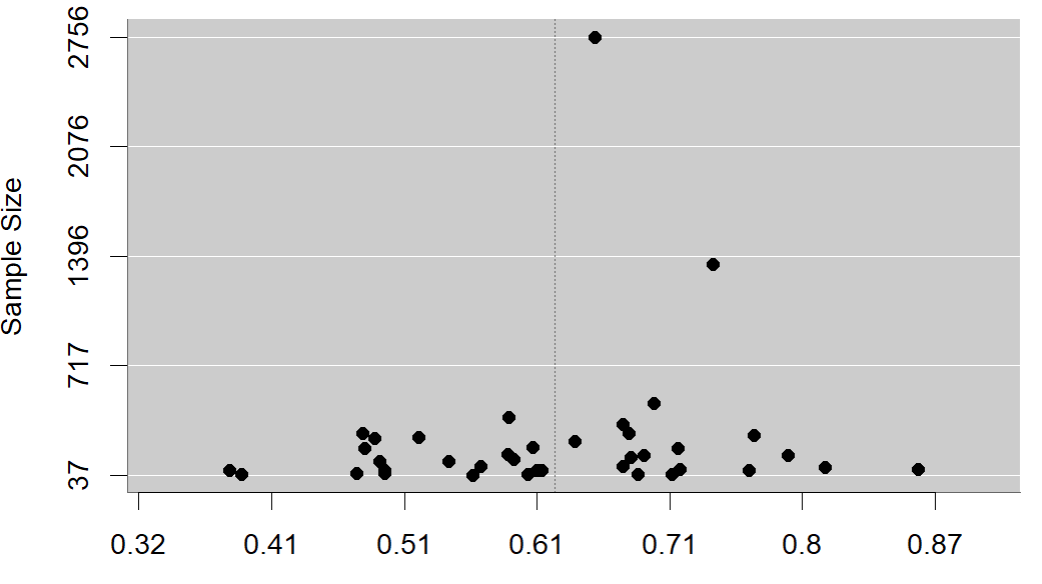


(a) original funnel (b) plot by sample size

***Figure S8***

***Funnel plots for the lifetime psychosis in patients with bipolar I***


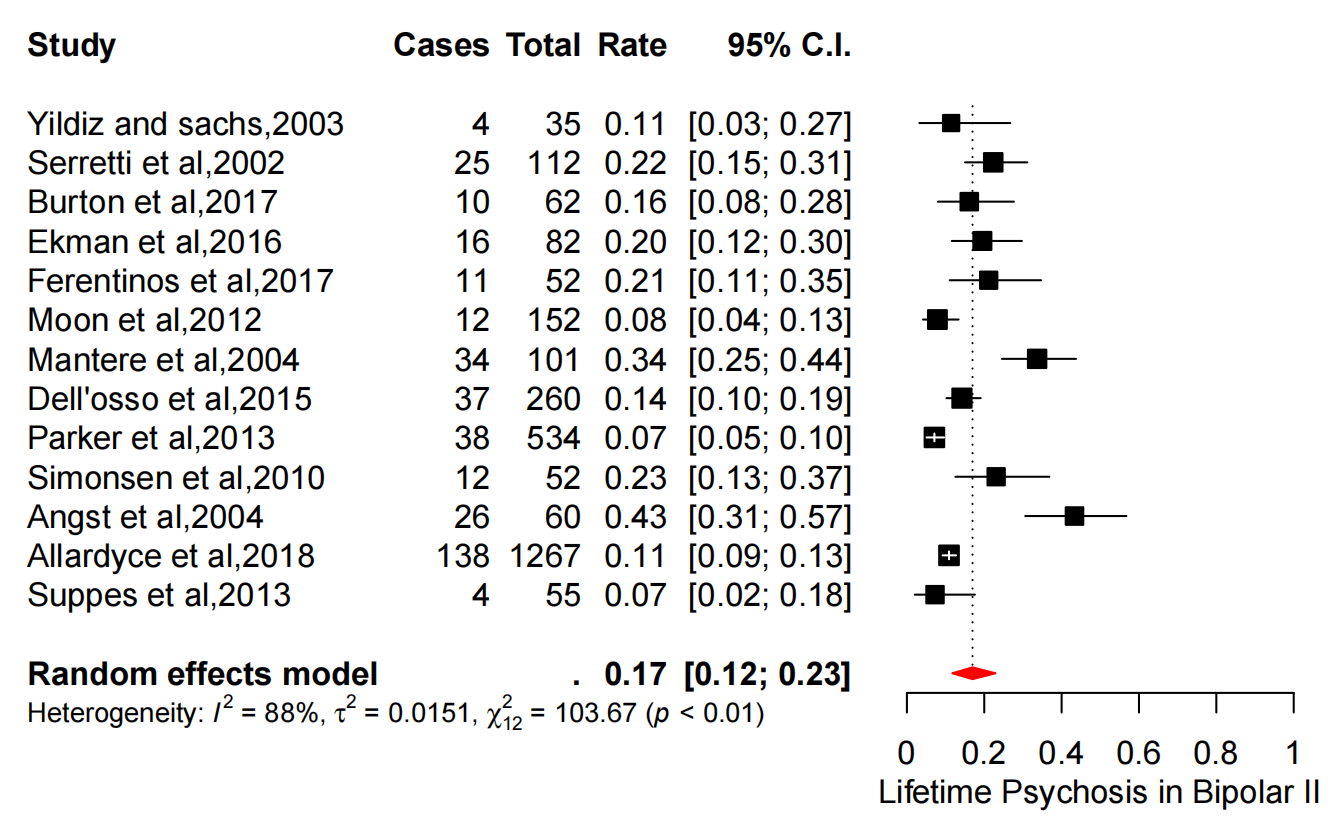


***Figure S9***

***The rate of lifetime psychosis in patients with bipolar II***


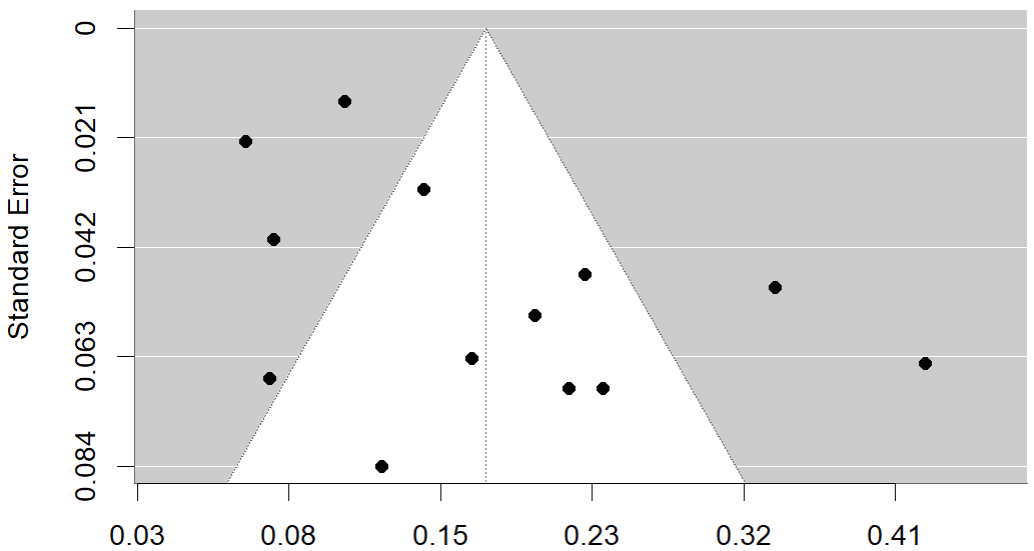

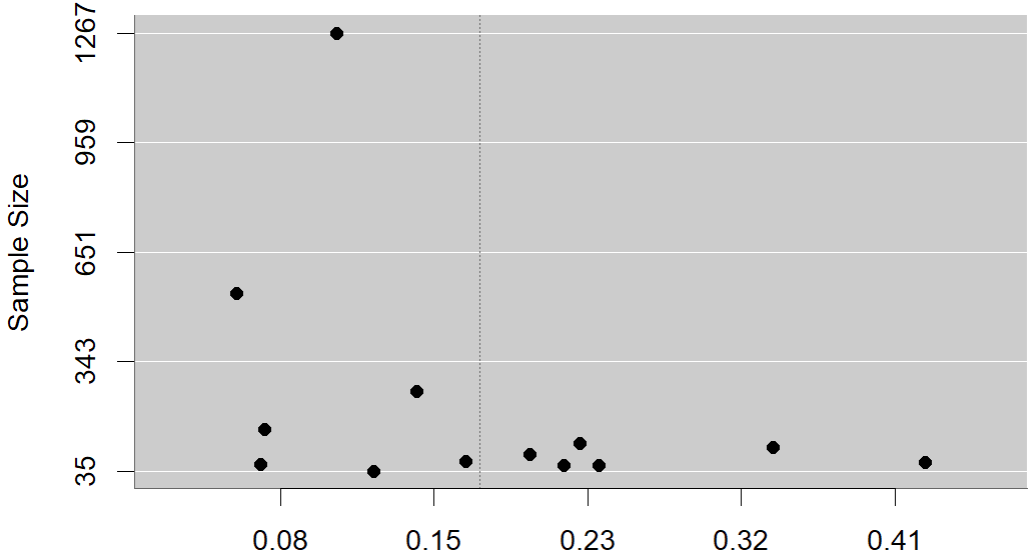


(a) original funnel (b) plot by sample size

***Figure S10***

***Funnel plots for lifetime psychosis in patients with bipolar II***


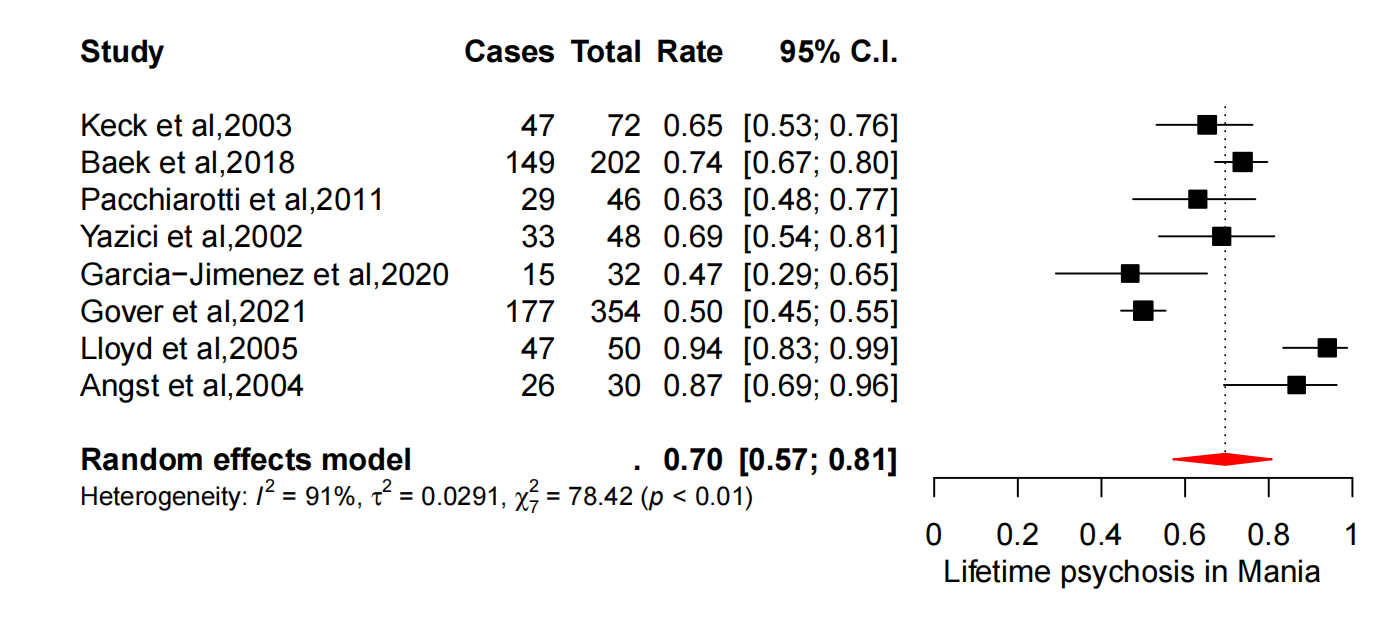


**Figure S11**

***Pooled rate of lifetime psychosis in bipolar patients with manic episode***


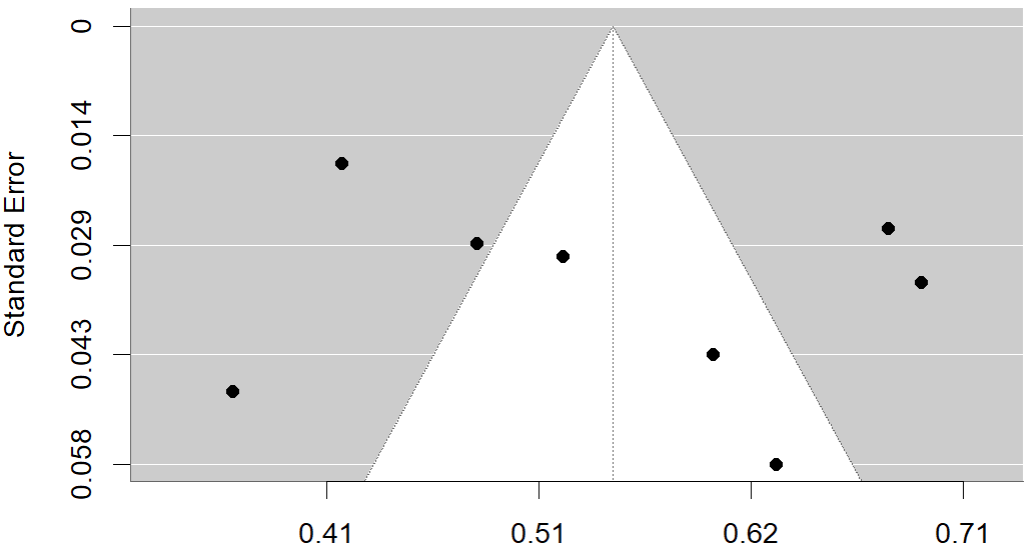

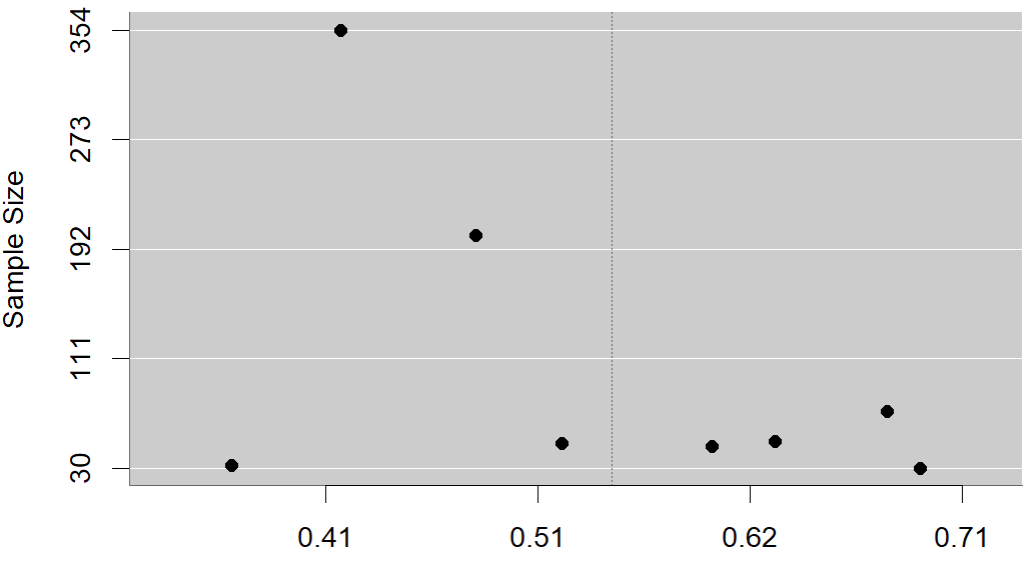


(a) original funnel (b) plot by sample size

***Figure S12***

***Funnel plots for lifetime psychosis in bipolar patients with manic episode***


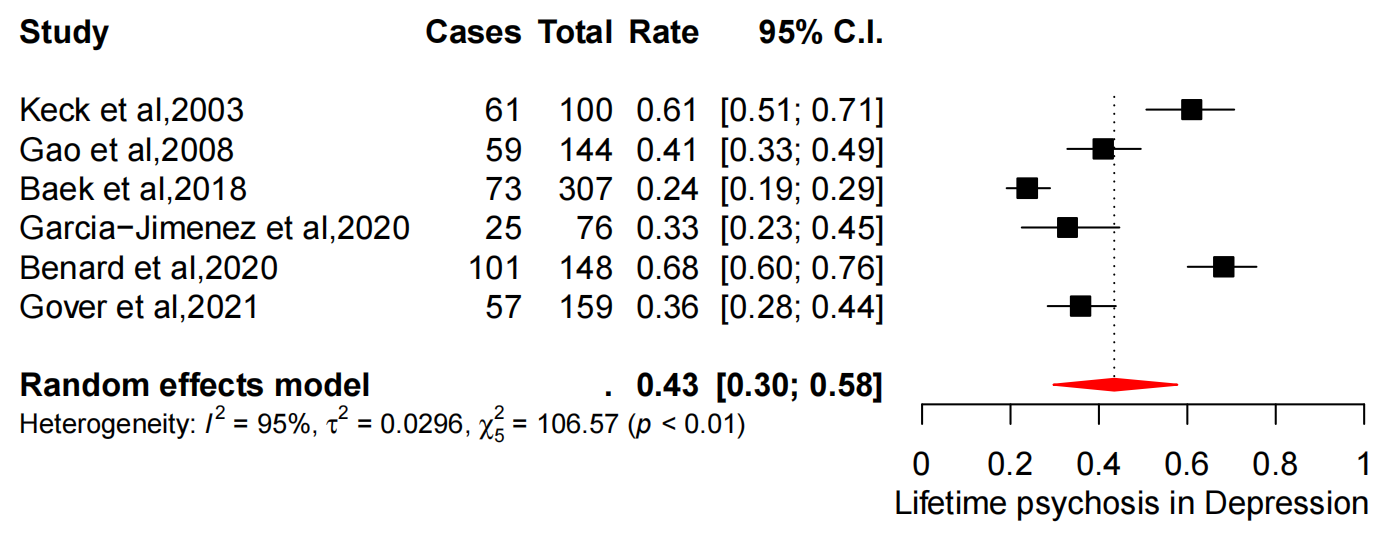


***Figure S13***

***Pooled rate of lifetime psychosis in bipolar patients with depressive episode***


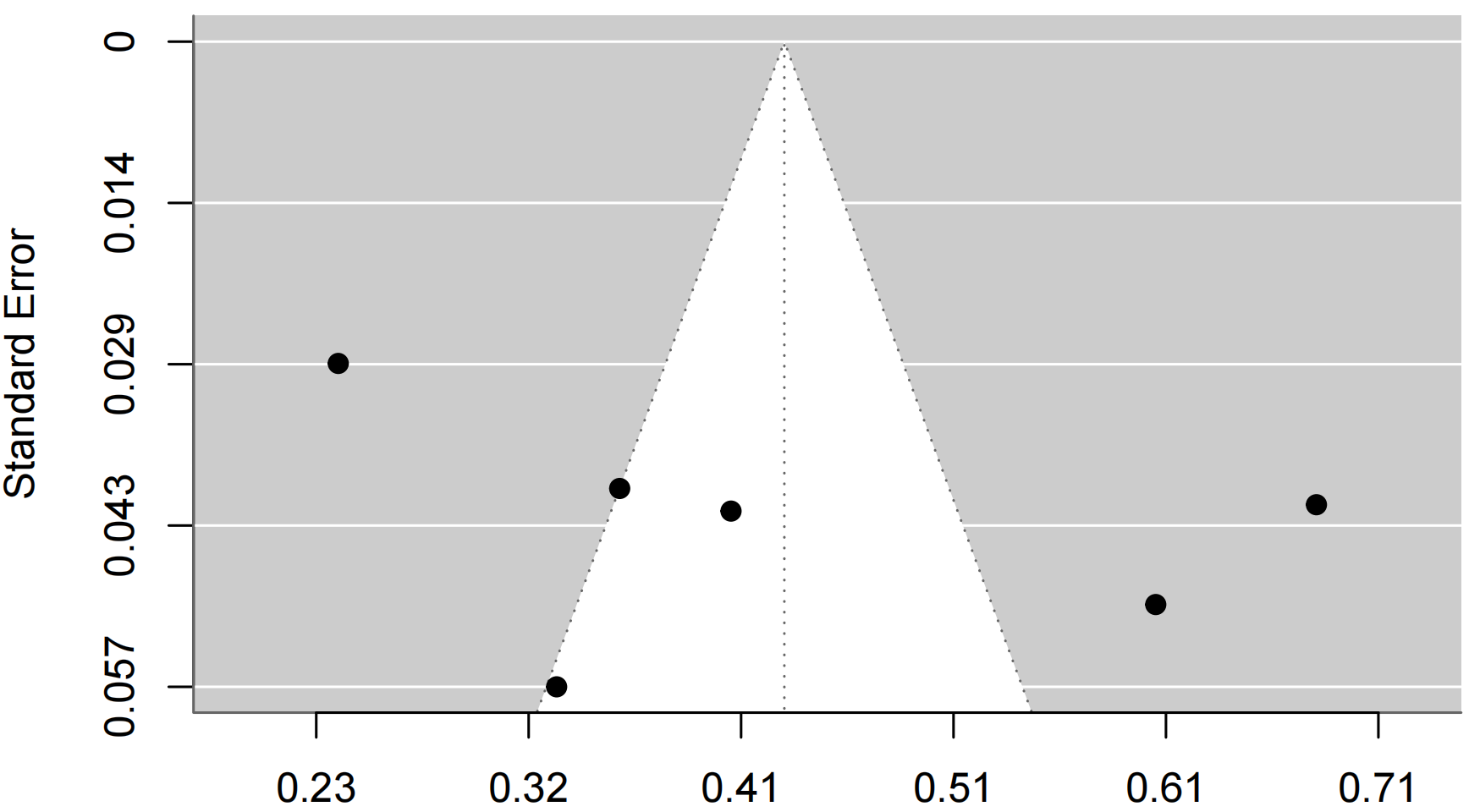

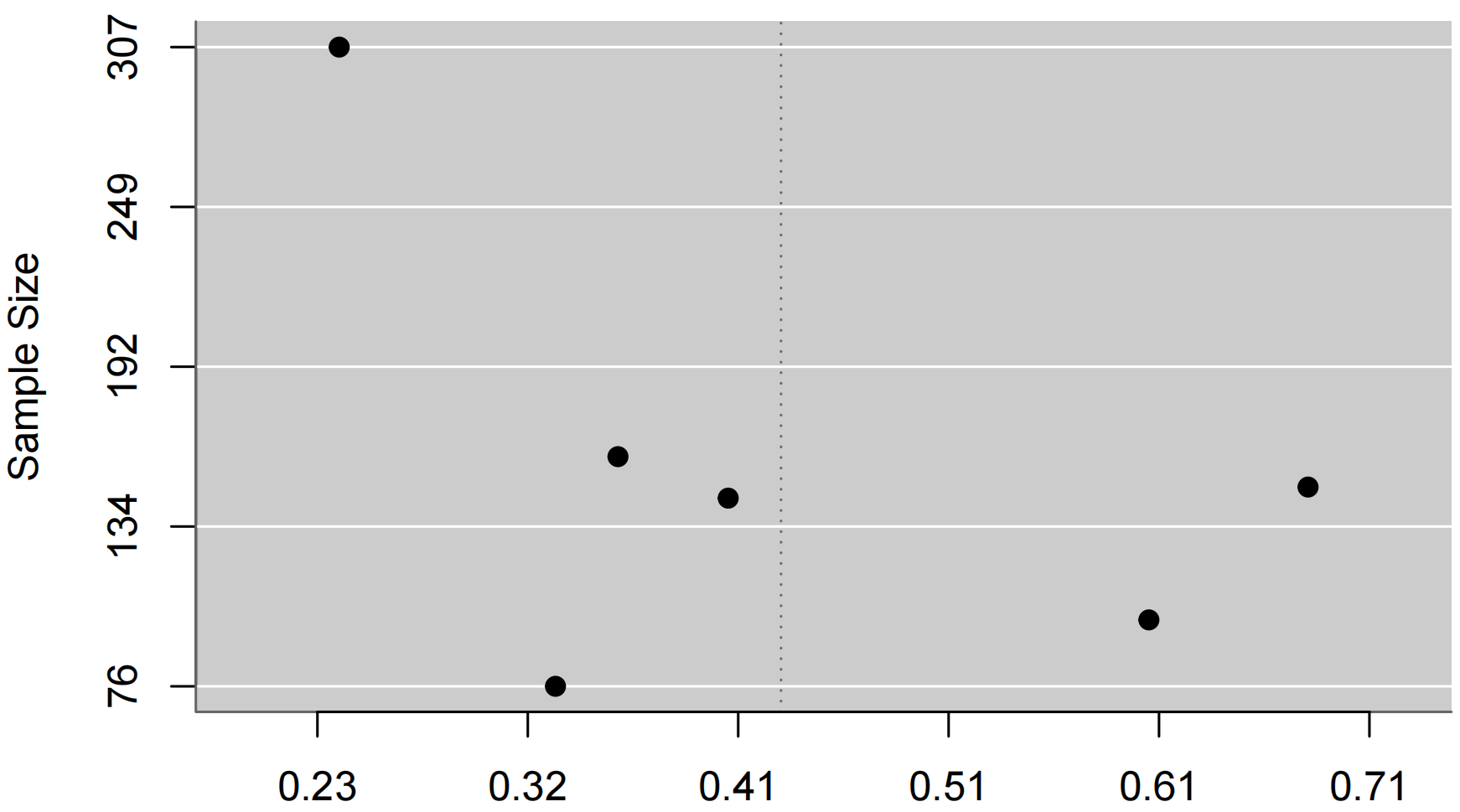


(a) original funnel (b) plot by sample size

***Figure S14***

***Funnel plots for lifetime psychosis in bipolar patients with depressive episode***

***
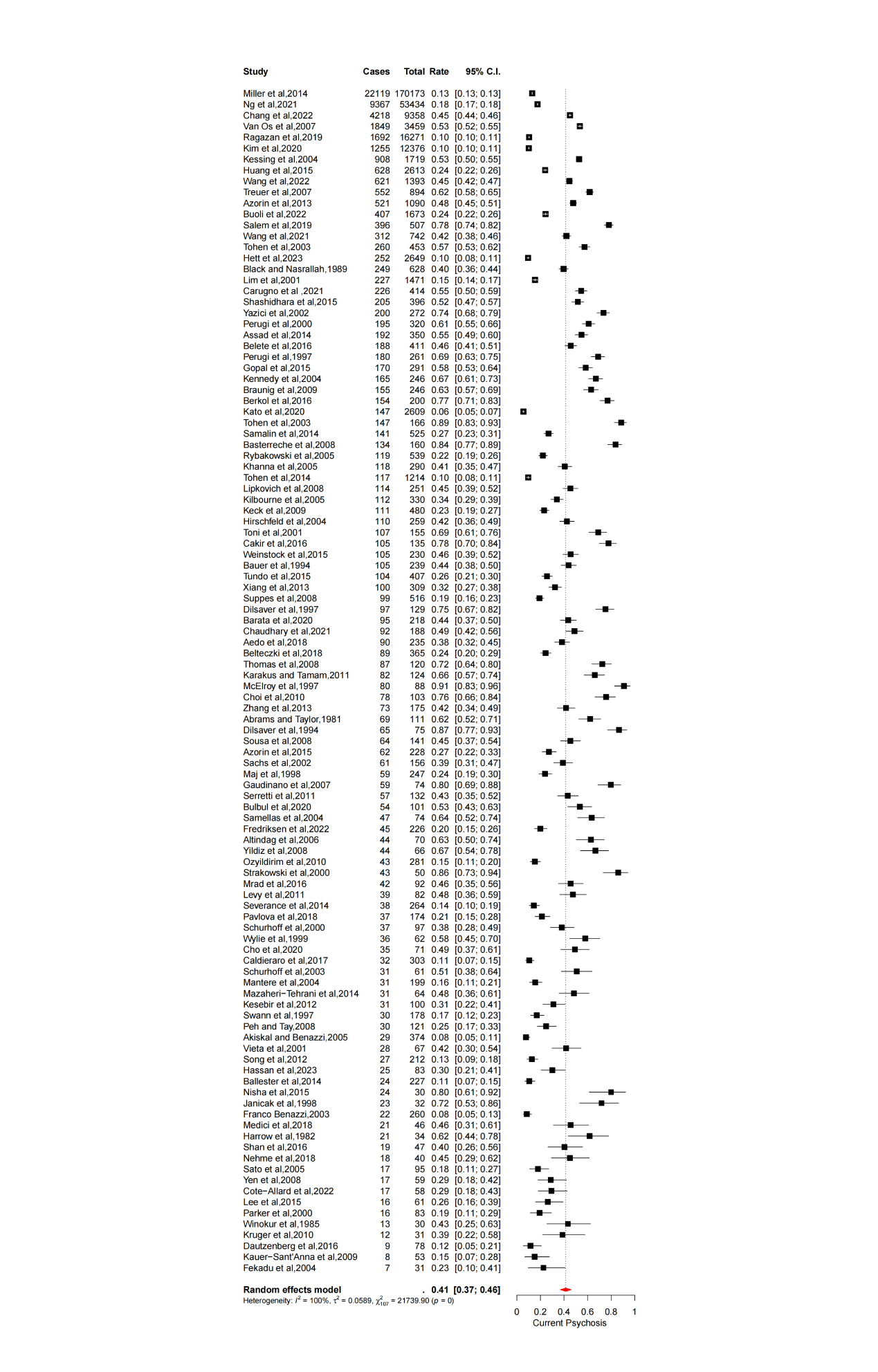
***

**Figure S15**

***Pooled rate of current psychosis in bipolar patients***


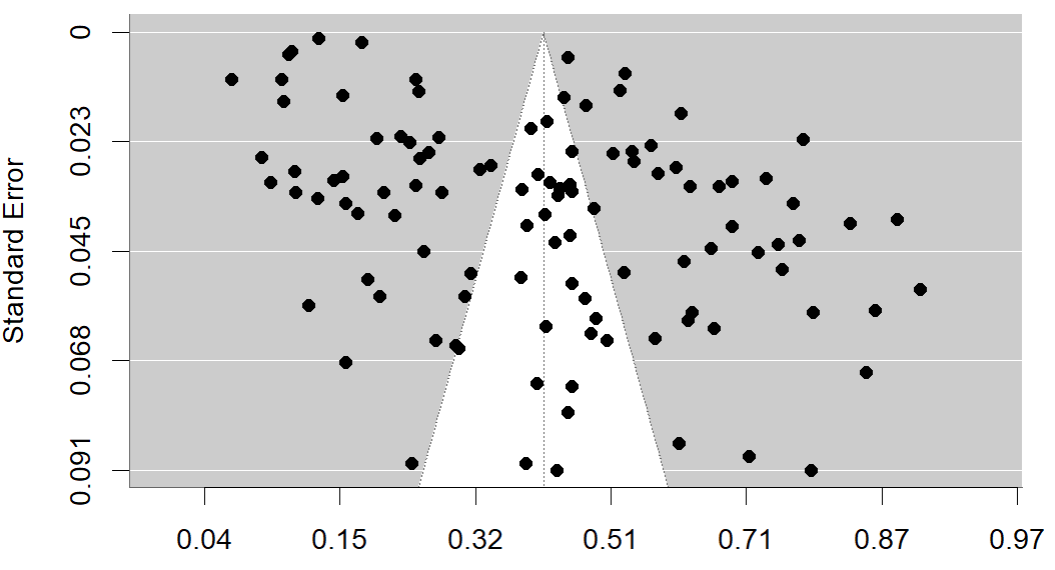

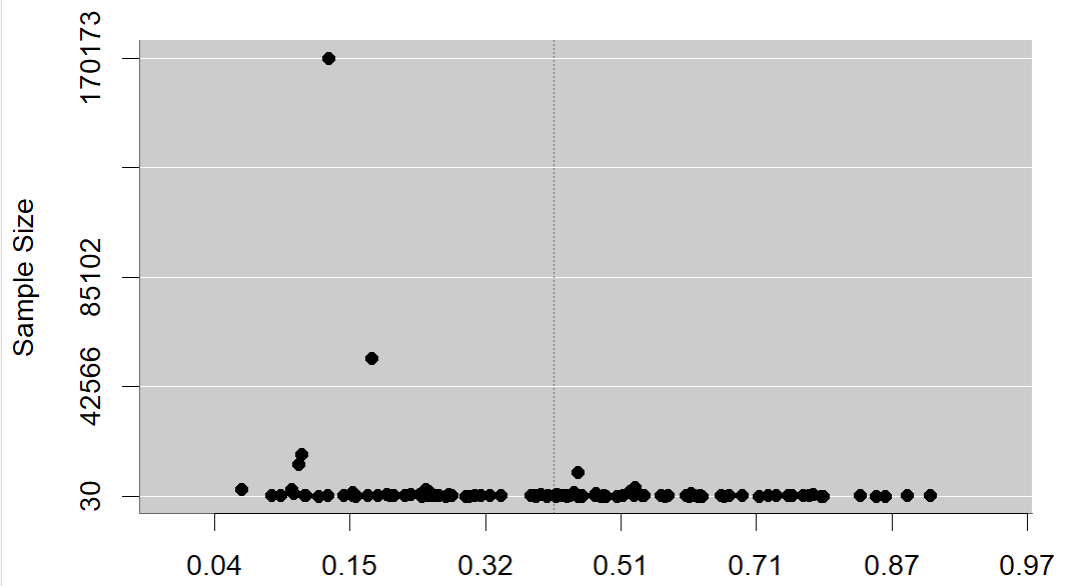


(a) original funnel (b) plot by sample size

**Figure S16**

***Funnel plots for current psychosis in bipolar patients***


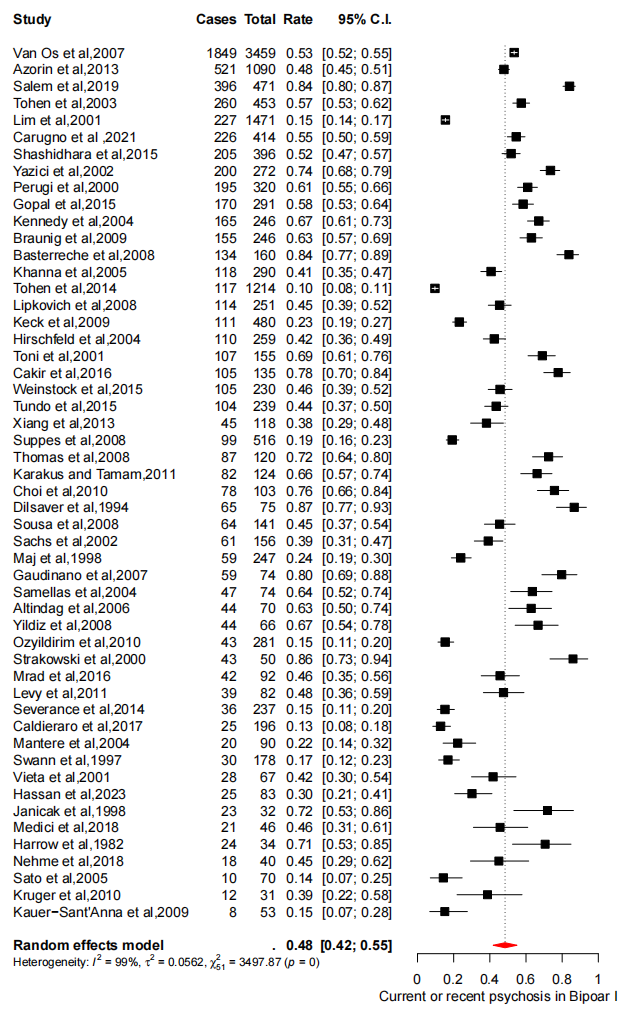


**Figure S17**

***Pooled rate of current psychosis in bipolar I patients***


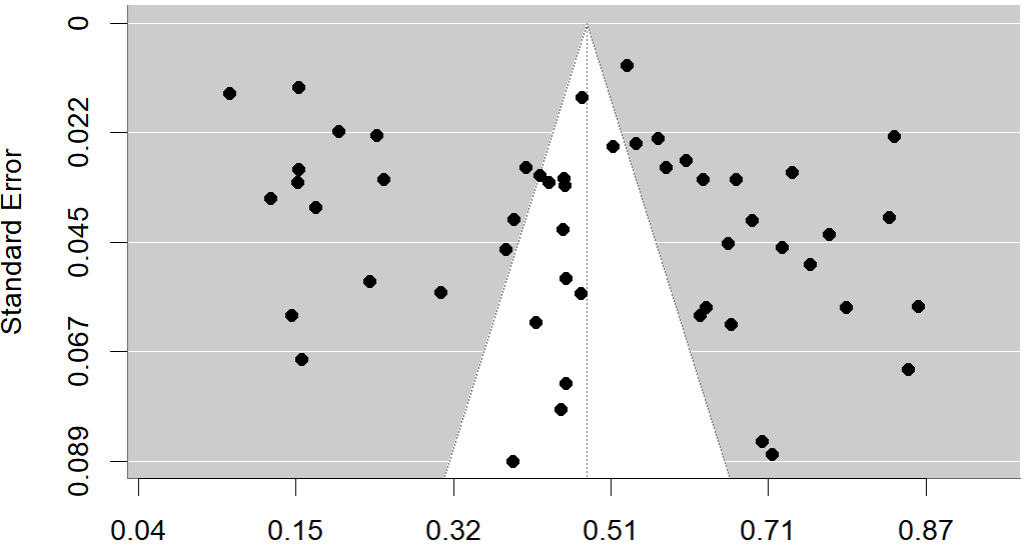

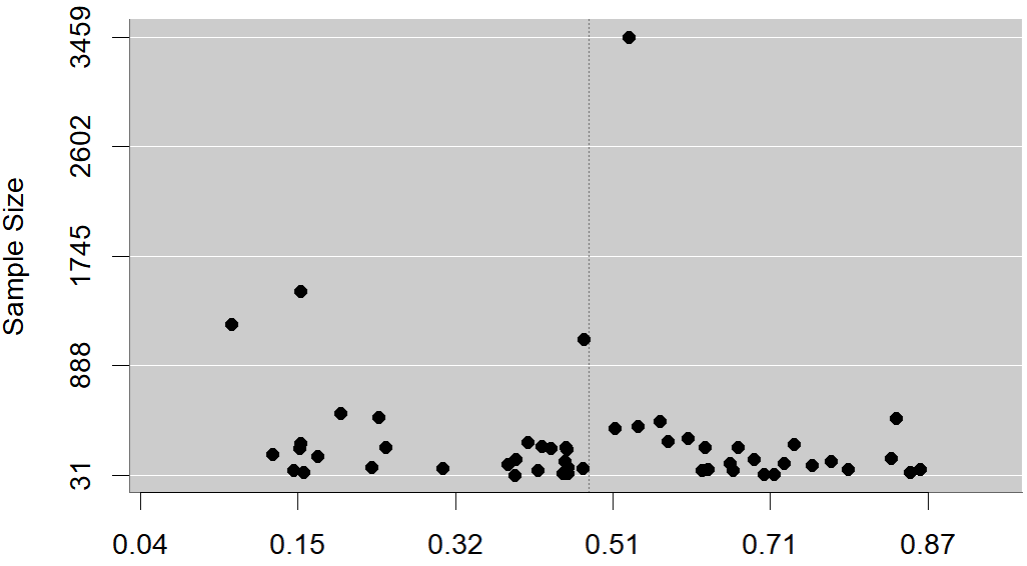


(a) original funnel (b) plot by sample size

**Figure S18**

***Funnel plots for current psychosis in bipolar I patients***


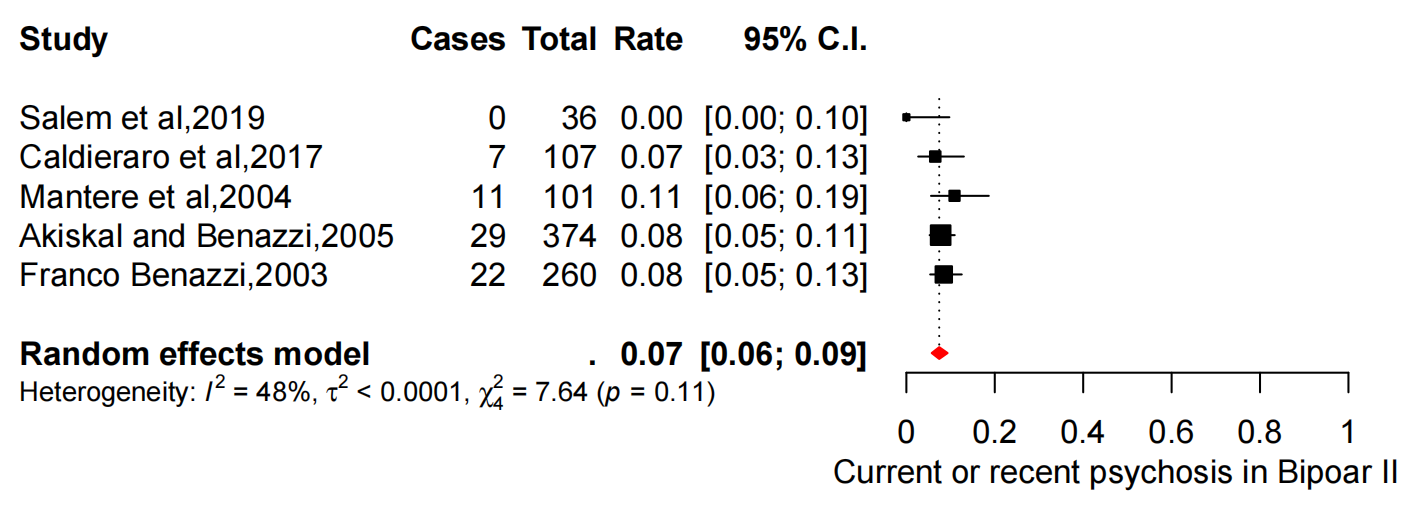


**Figure S19**

***Pooled rate of current psychosis in bipolar II patients***


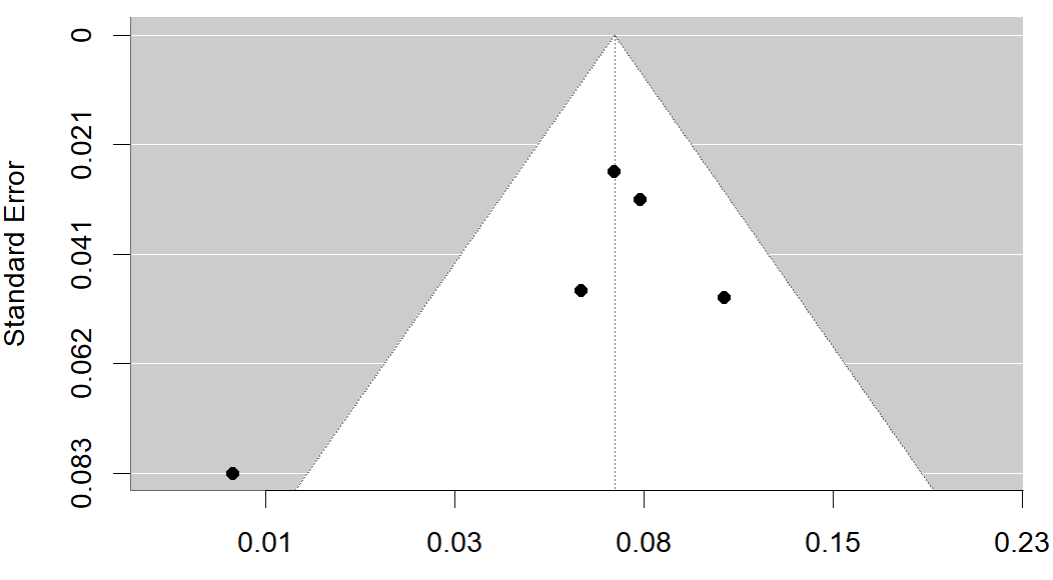

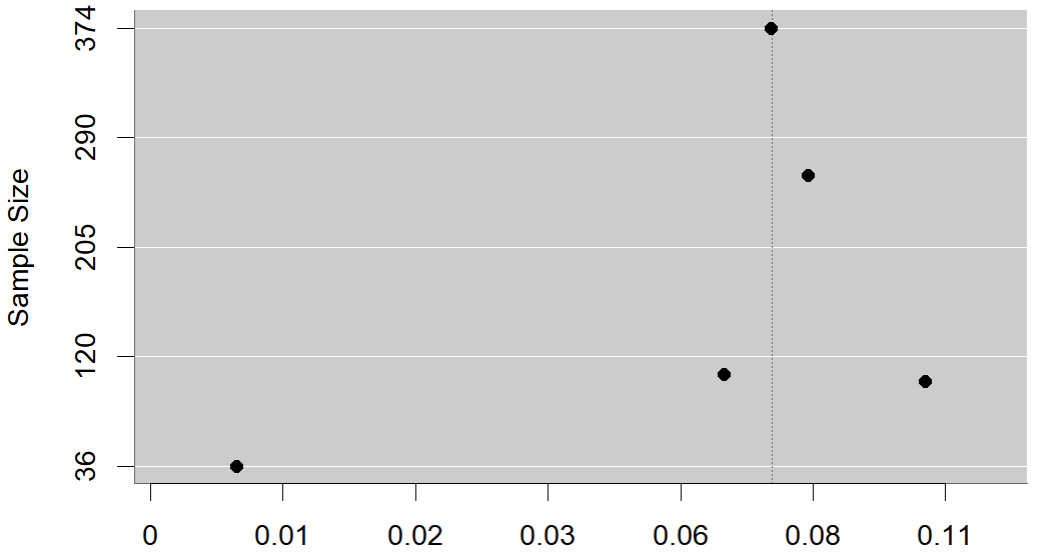


(a) original funnel (b) plot by sample size

**Figure S20**

***Funnel plots for current psychosis in bipolar II patients***


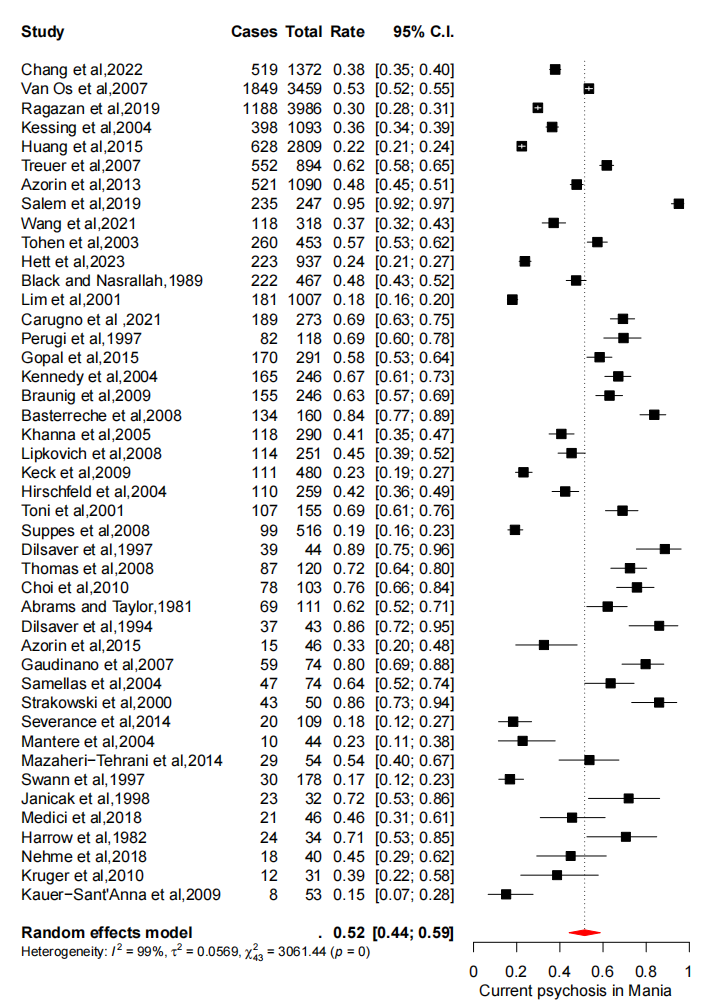


**Figure S21**

***Pooled rate of current psychosis in bipolar patients with manic episode***


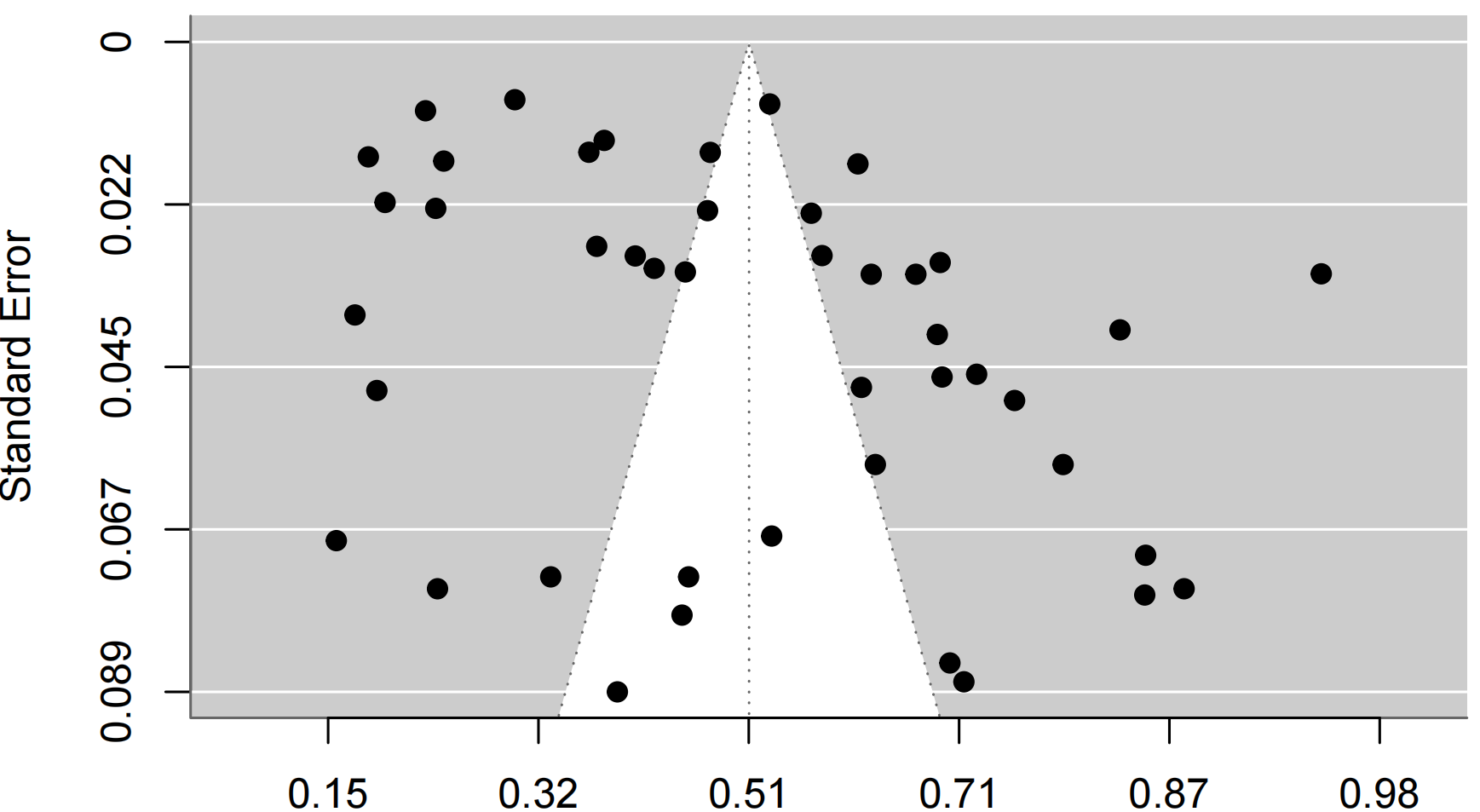

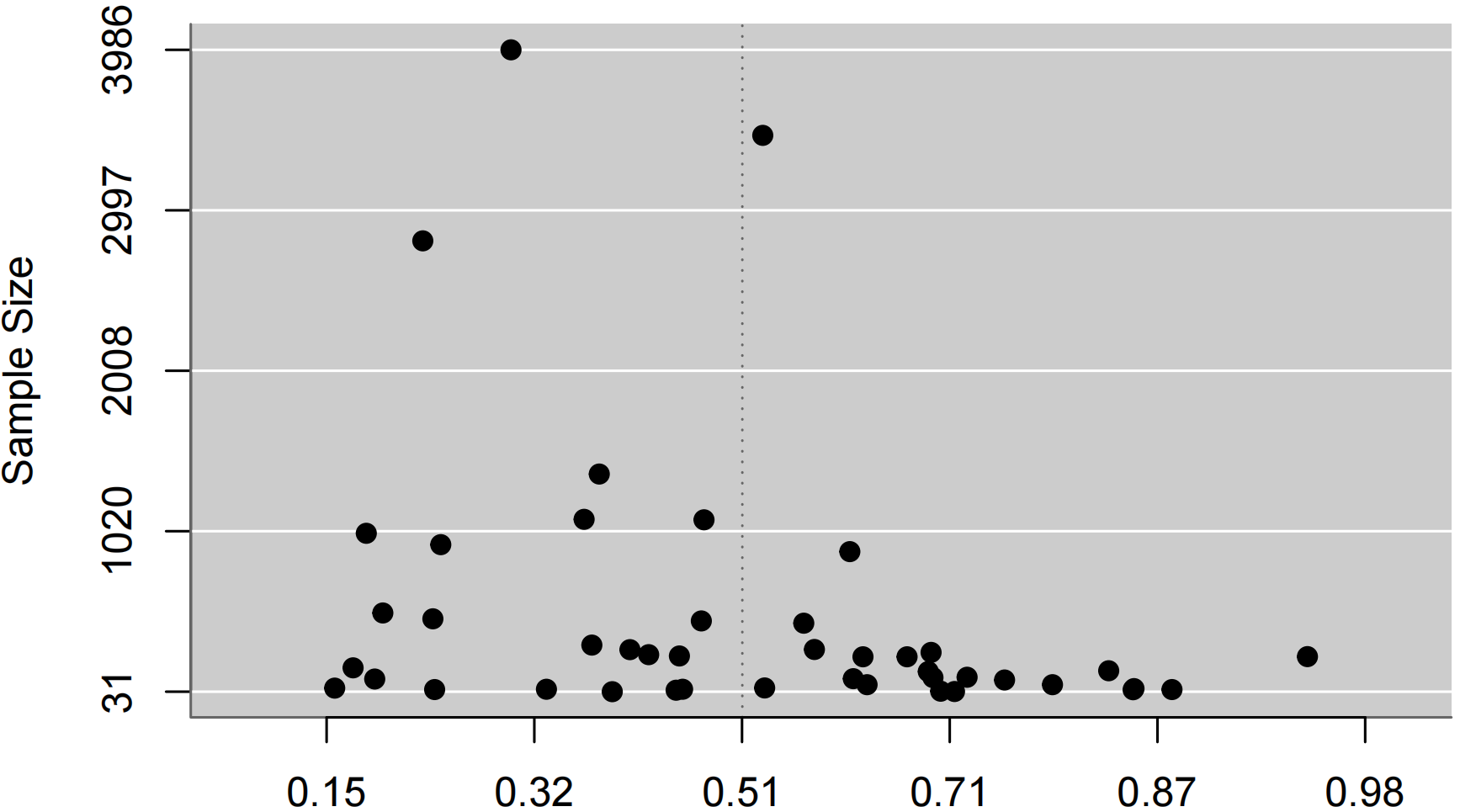


(a) original funnel (b) plot by sample size

**Figure S22**

***Funnel plots for current psychosis in bipolar patients with manic episode***


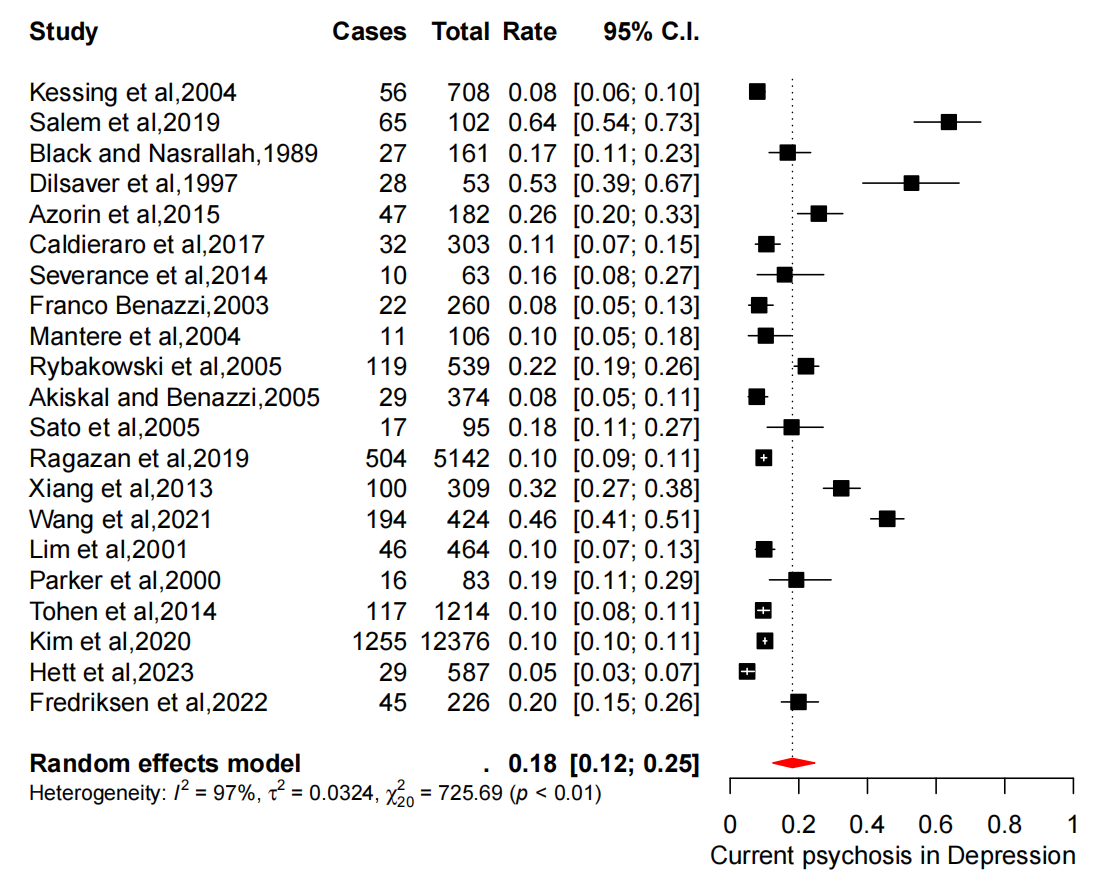


**Figure S23**

***Pooled rate of current psychosis in bipolar patients with depressive episode***


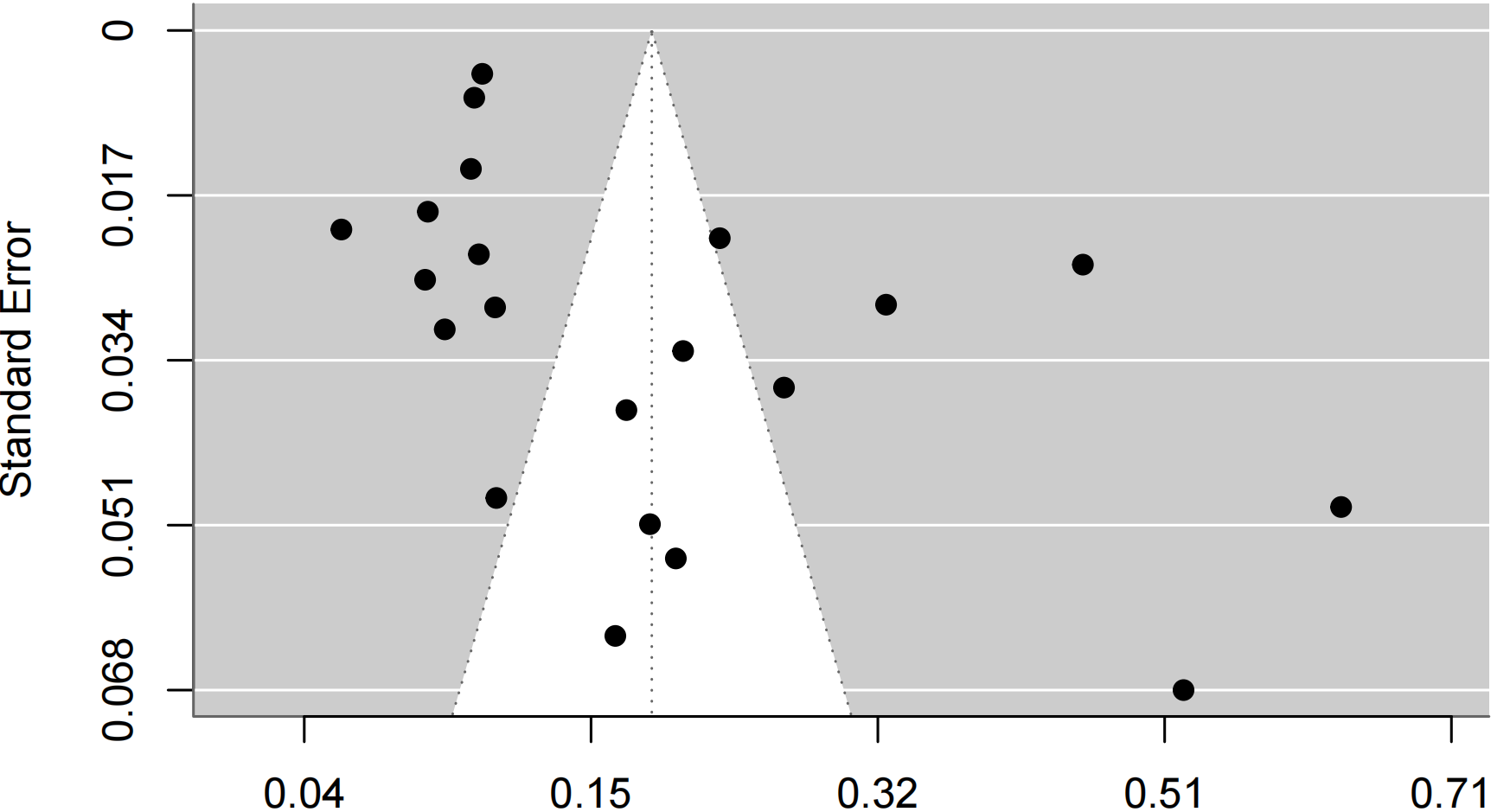

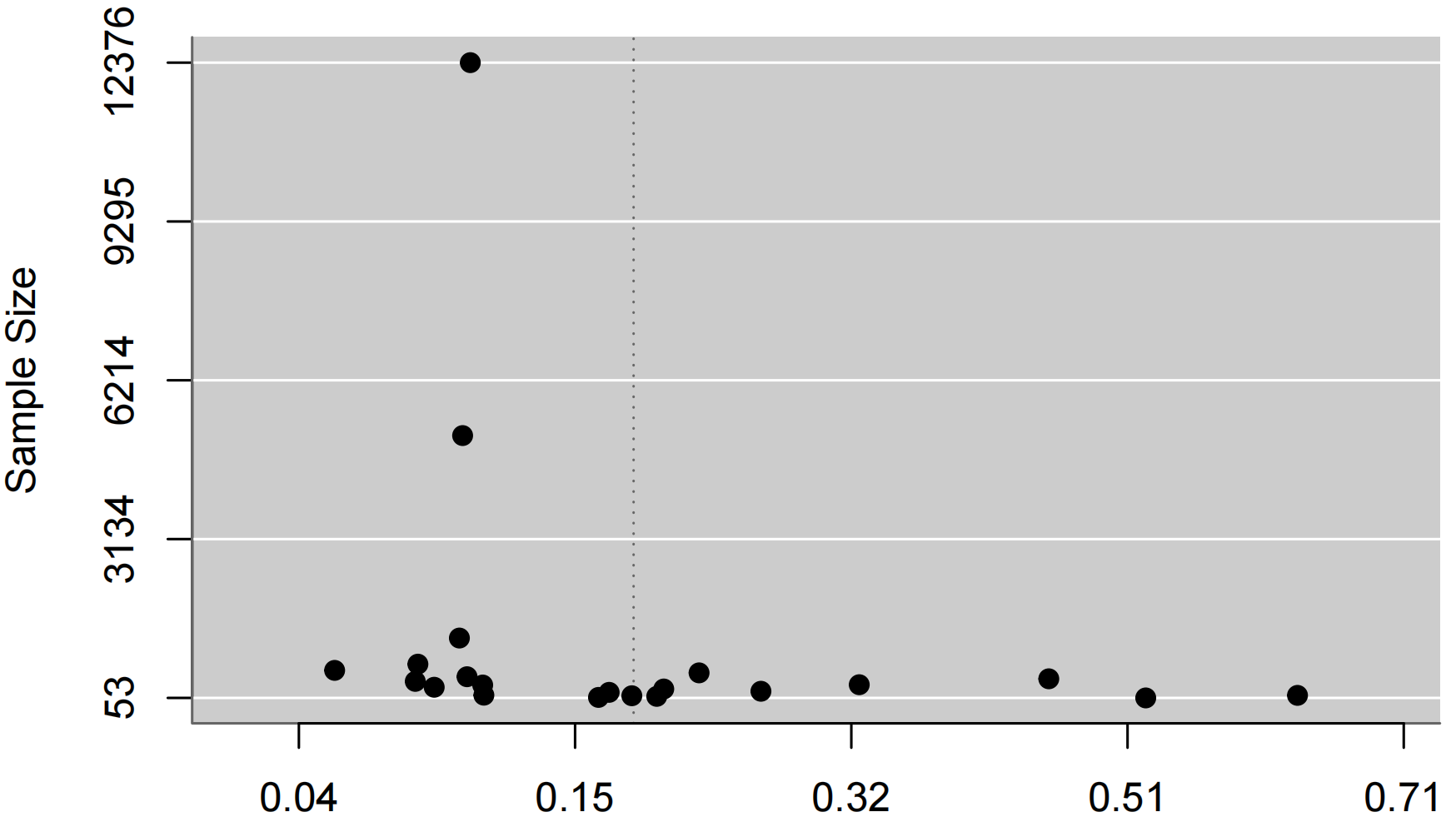


(a) original funnel (b) plot by sample size

**Figure S24**

***Funnel plots for current psychosis in bipolar patients with depressive episode***


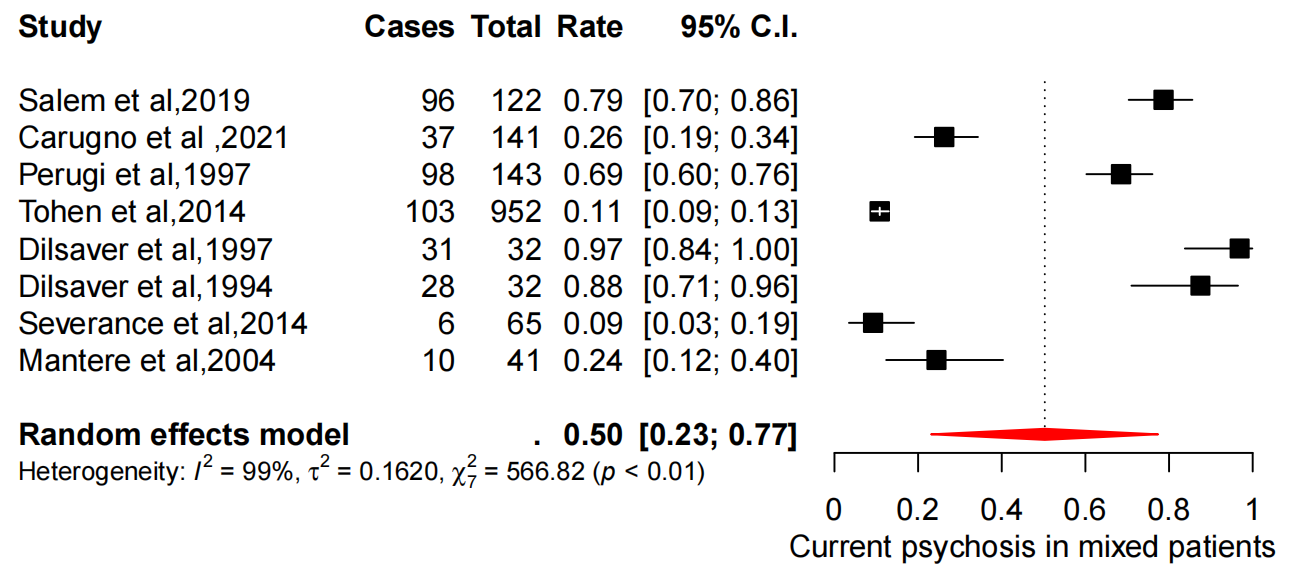


**Figure S25**

***Pooled rate of current psychosis in bipolar patients with mixed episode***


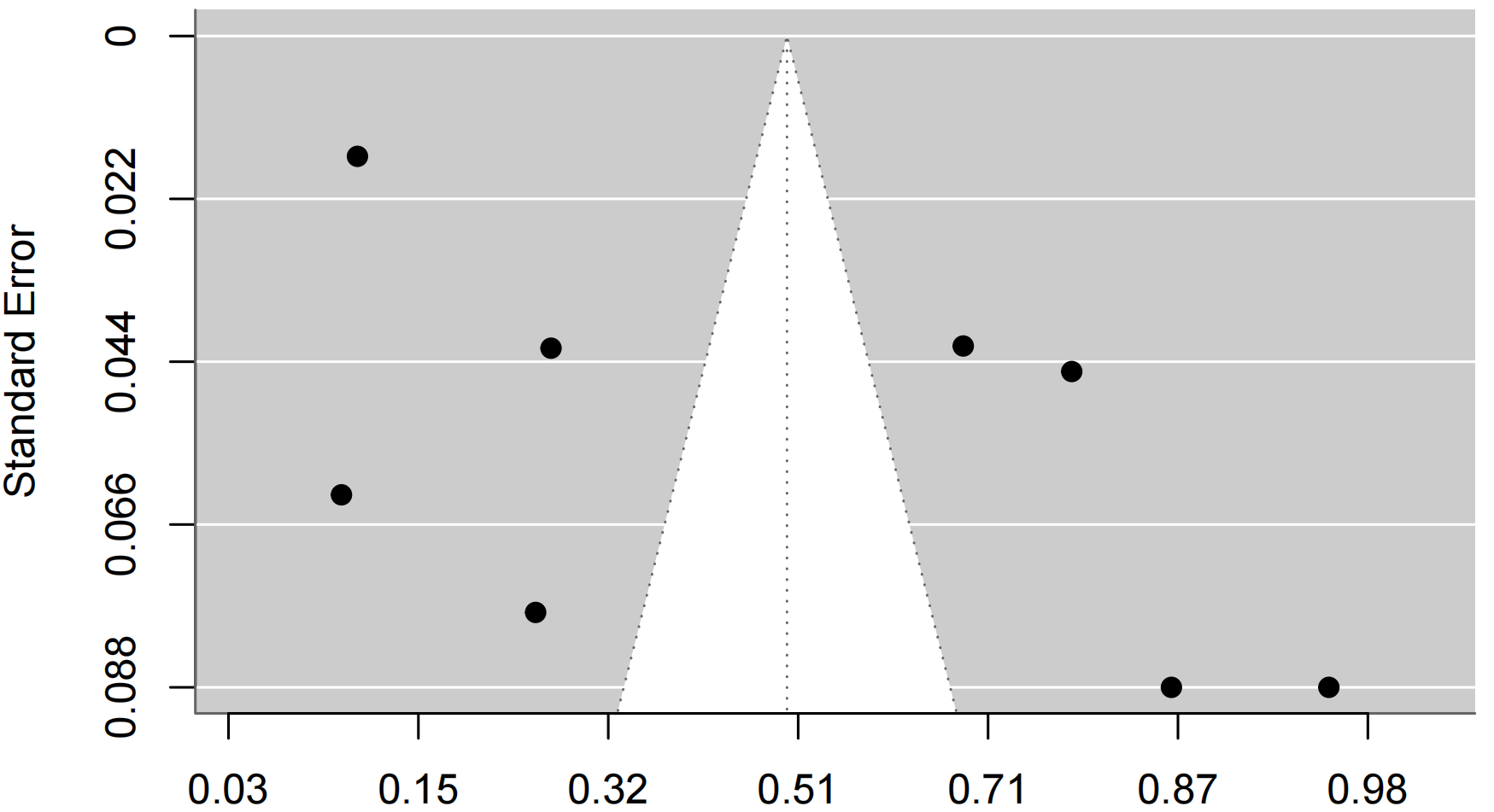

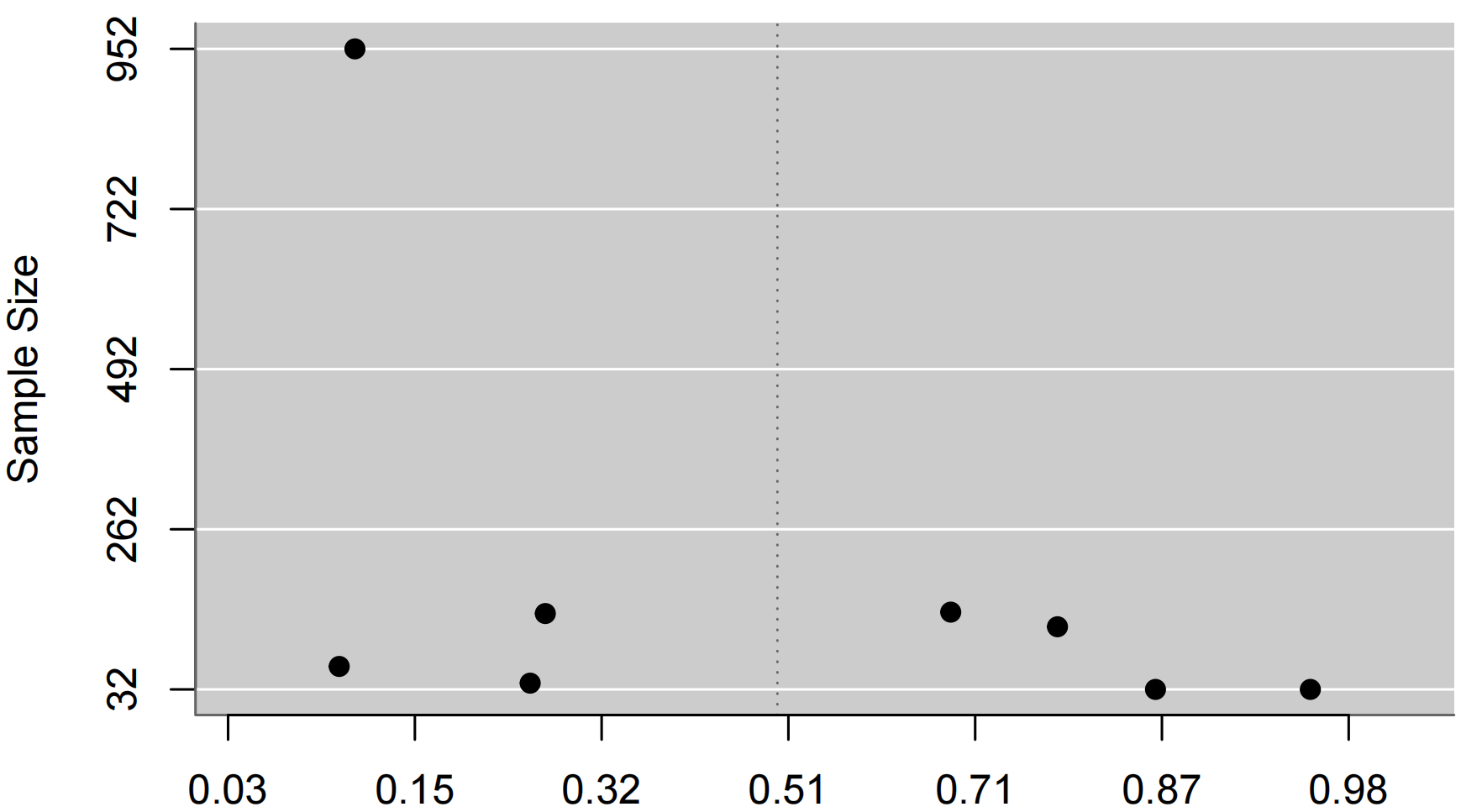


(a) original funnel (b) plot by sample size

**Figure S26**

***Funnel plots for current psychosis in bipolar patients with mixed episode***


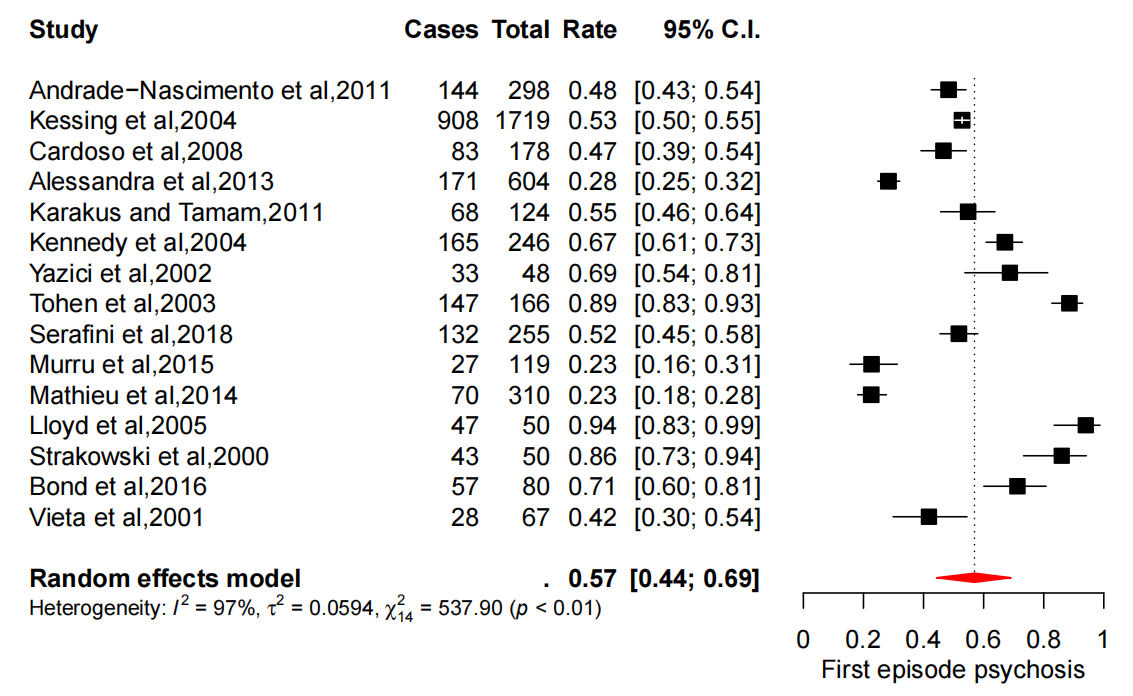


**Figure S27**

***Pooled rate of first episode psychosis in bipolar patients***


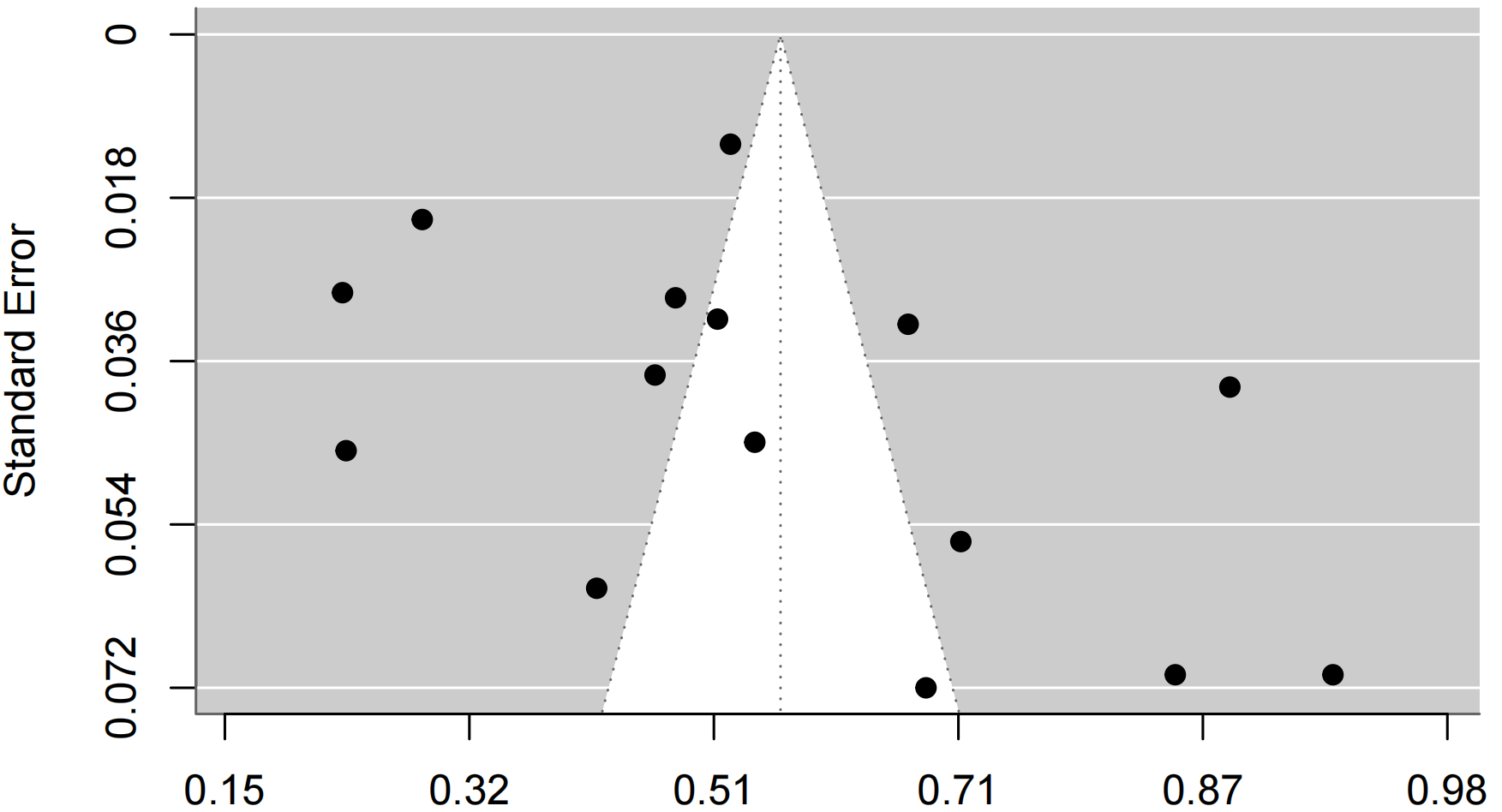

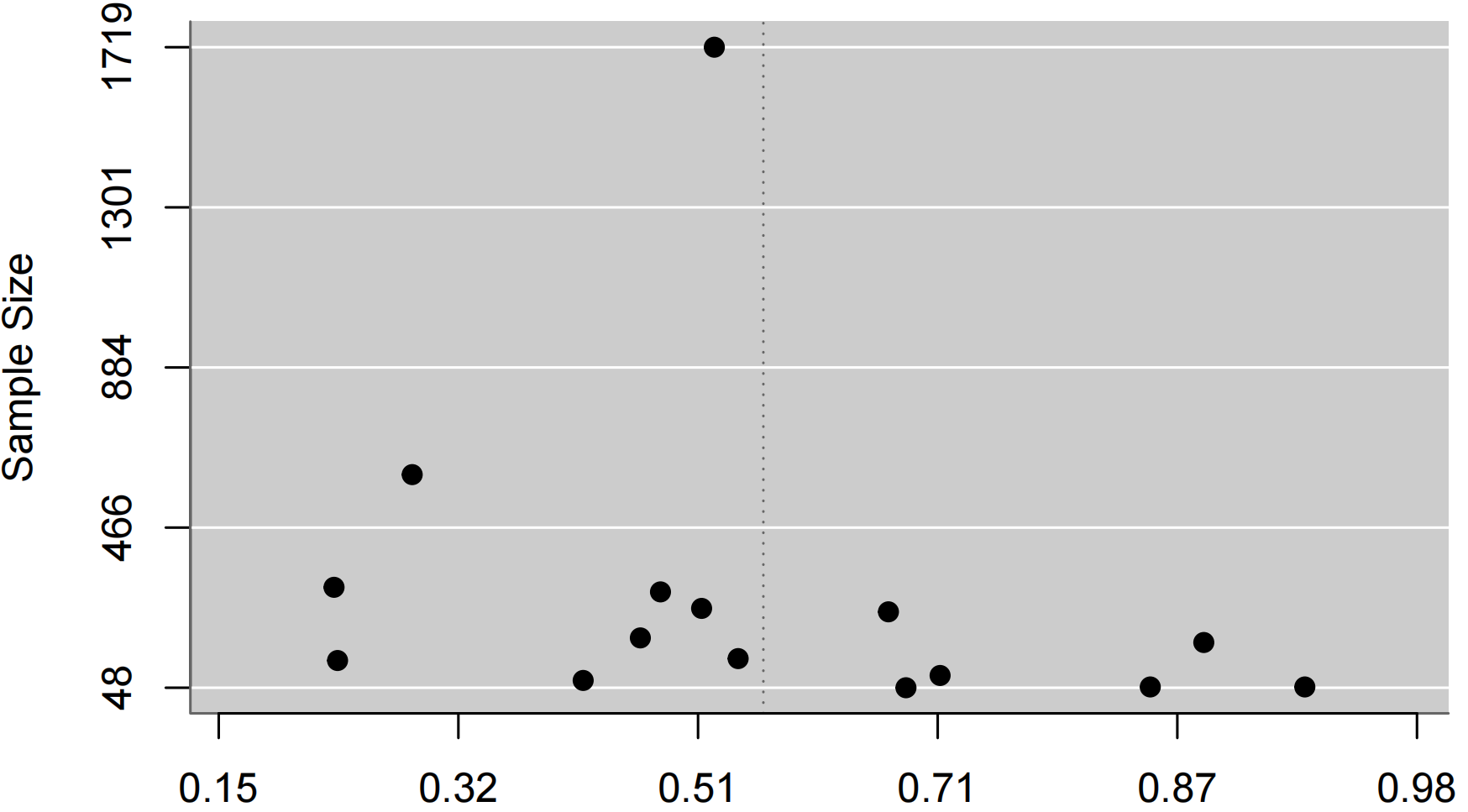


(a) original funnel (b) plot by sample size

**Figure S28**

***Funnel plots for first episode psychosis in bipolar patients***


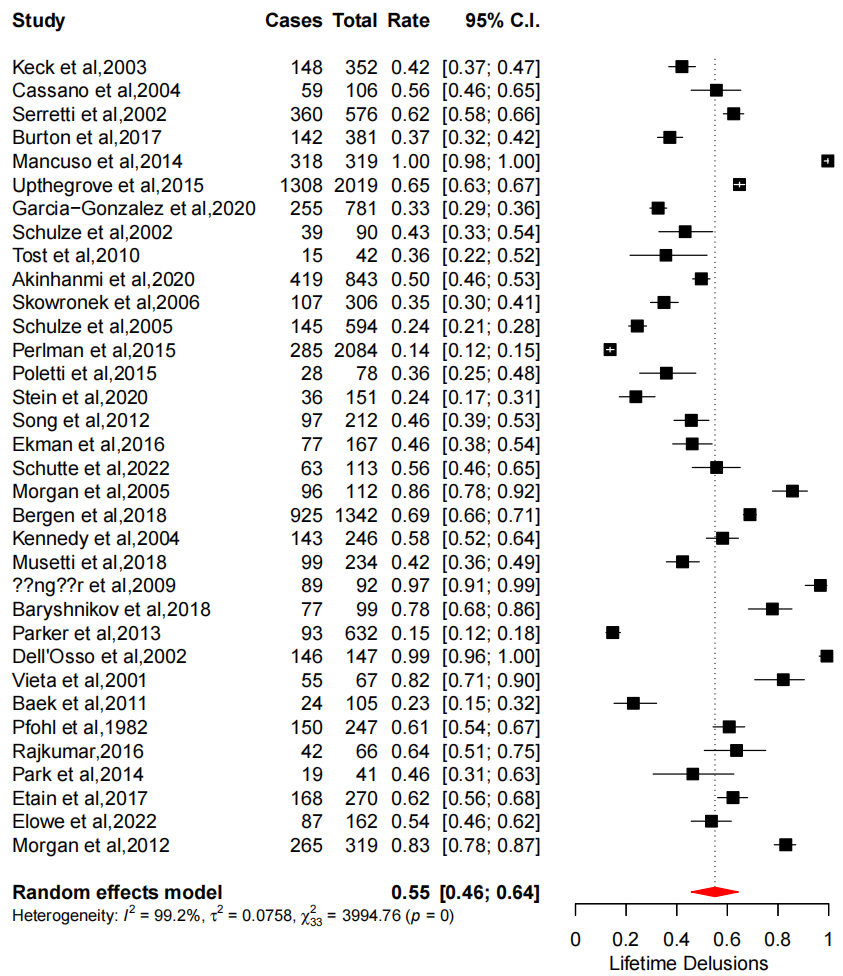


**Figure S29**

***Pooled rate of lifetime delusions in bipolar patients***


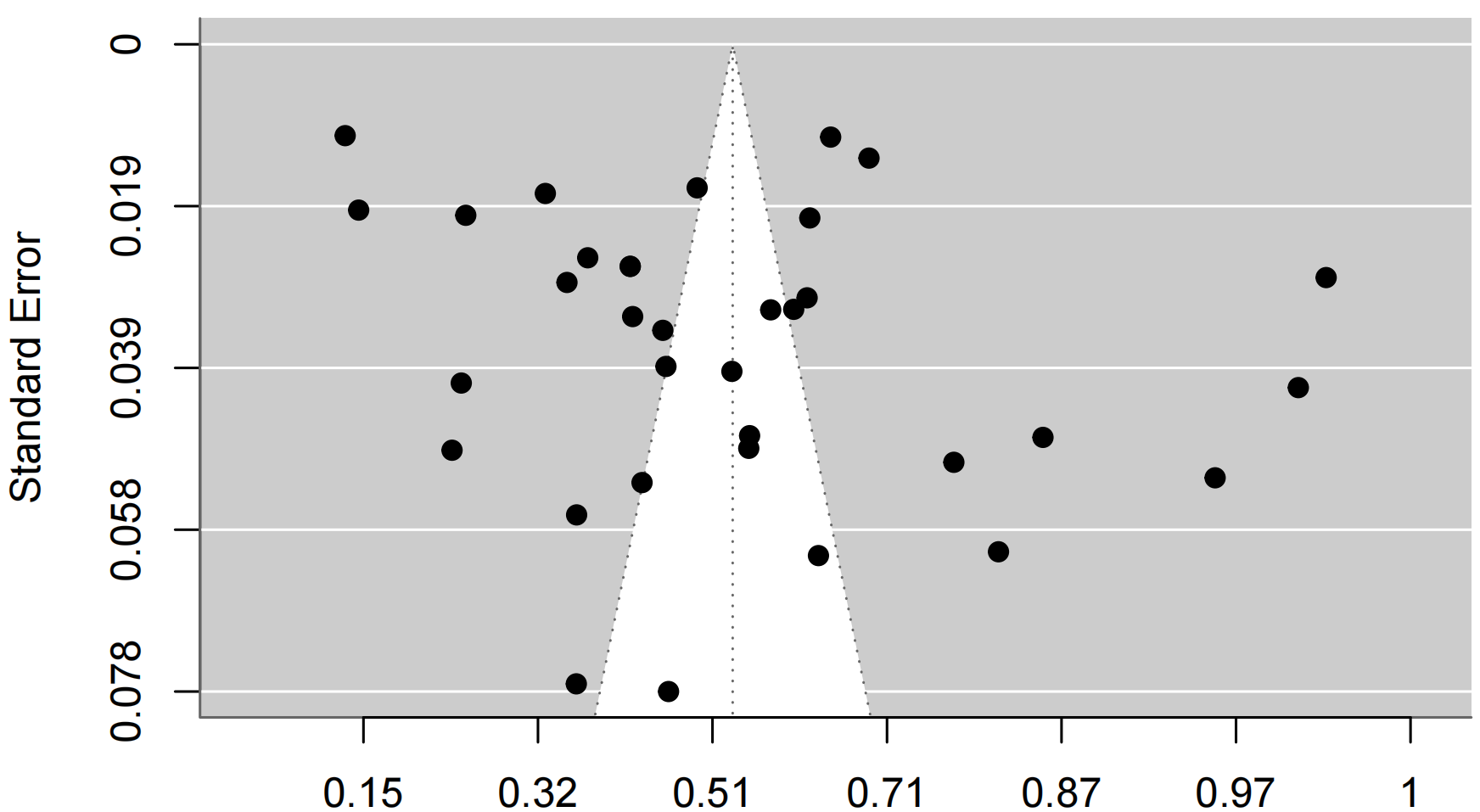

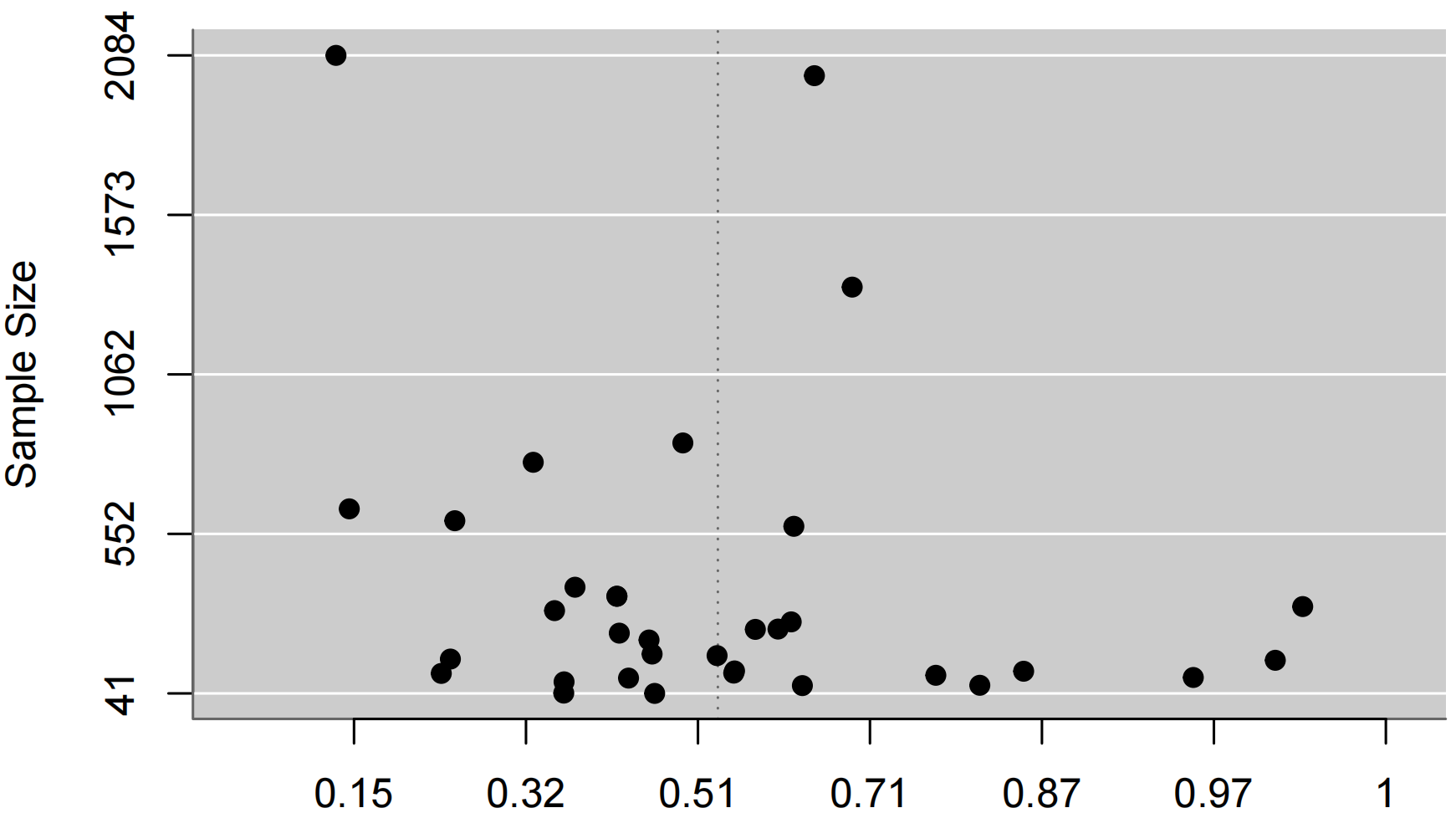


(a) original funnel (b) plot by sample size

**Figure S30**

***Funnel plots for lifetime delusions in bipolar patients***


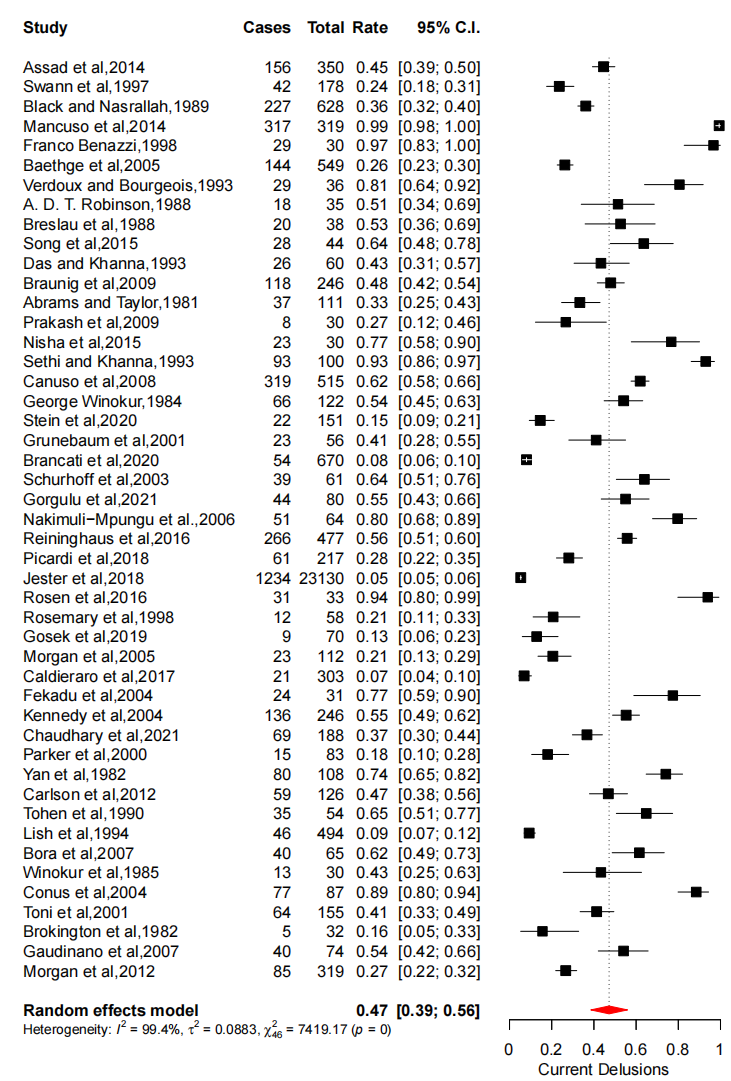


**Figure S31**

***Pooled rate of current delusions in bipolar patients***


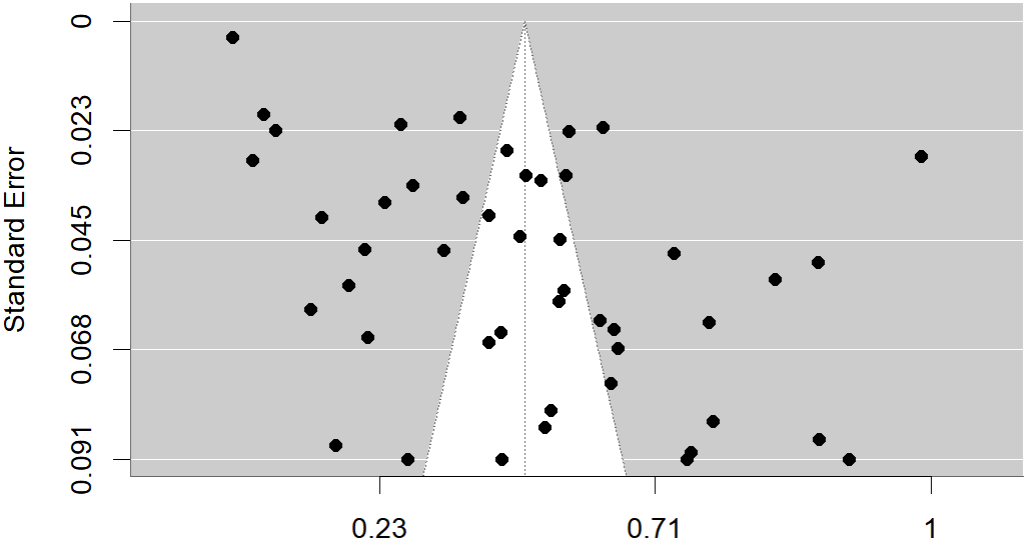

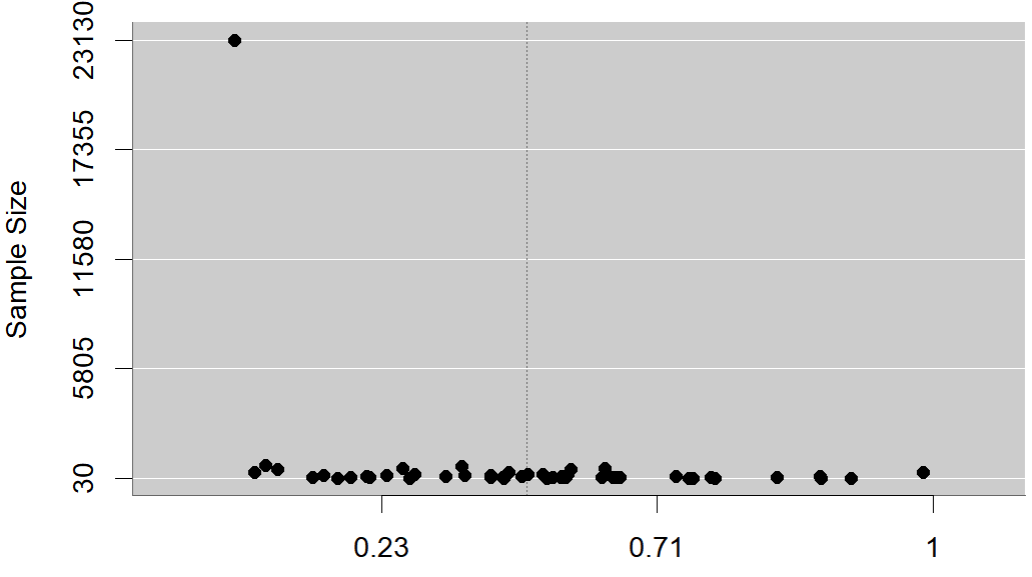


(a) original funnel (b) plot by sample size

**Figure S32**

***Funnel plots for current delusions in bipolar patients***


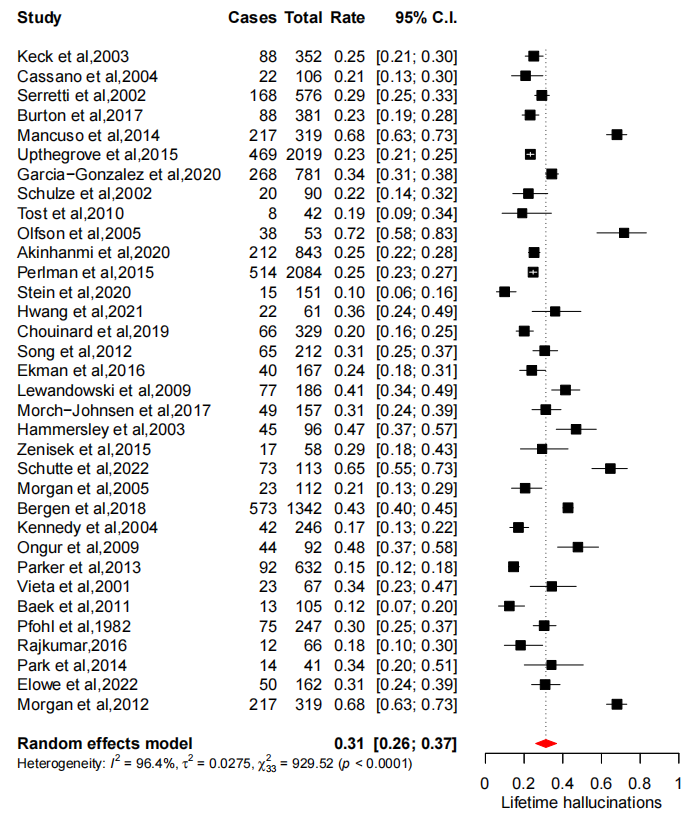


**Figure S33**

***Pooled rate of lifetime hallucinations in bipolar patients***


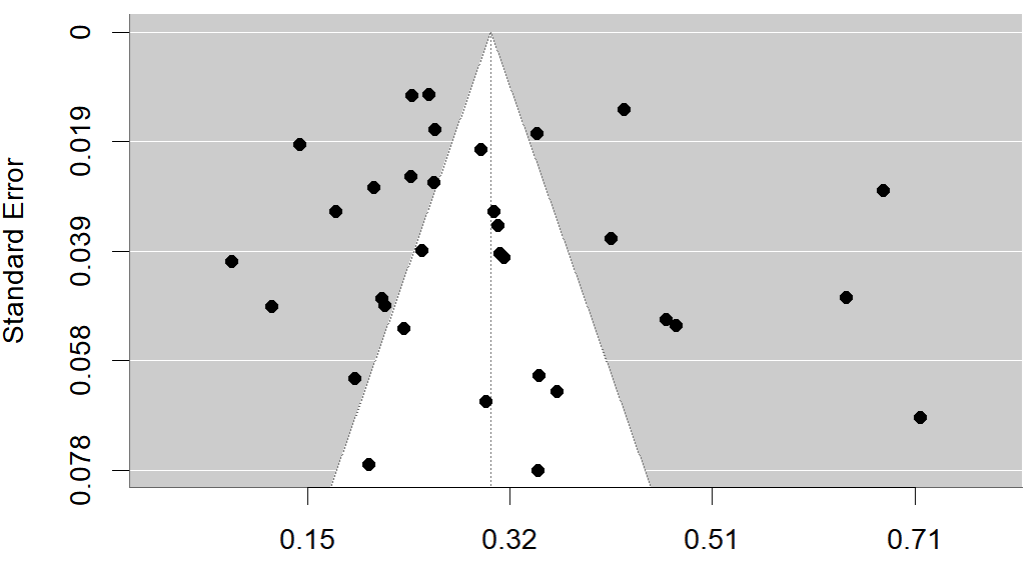

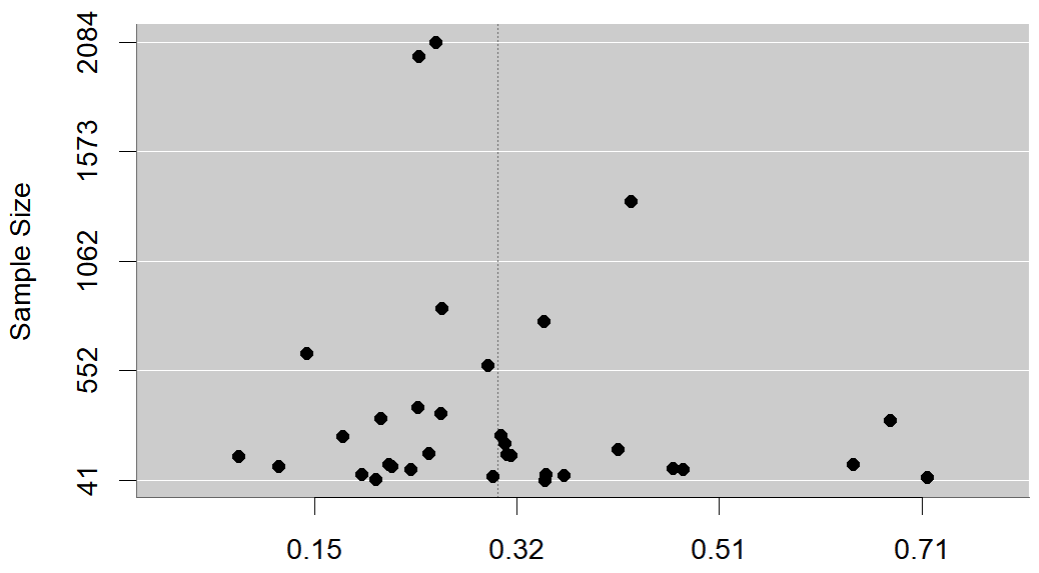


(a) original funnel (b) plot by sample size

**Figure S34**

***Funnel plots for lifetime hallucinations in bipolar patients***


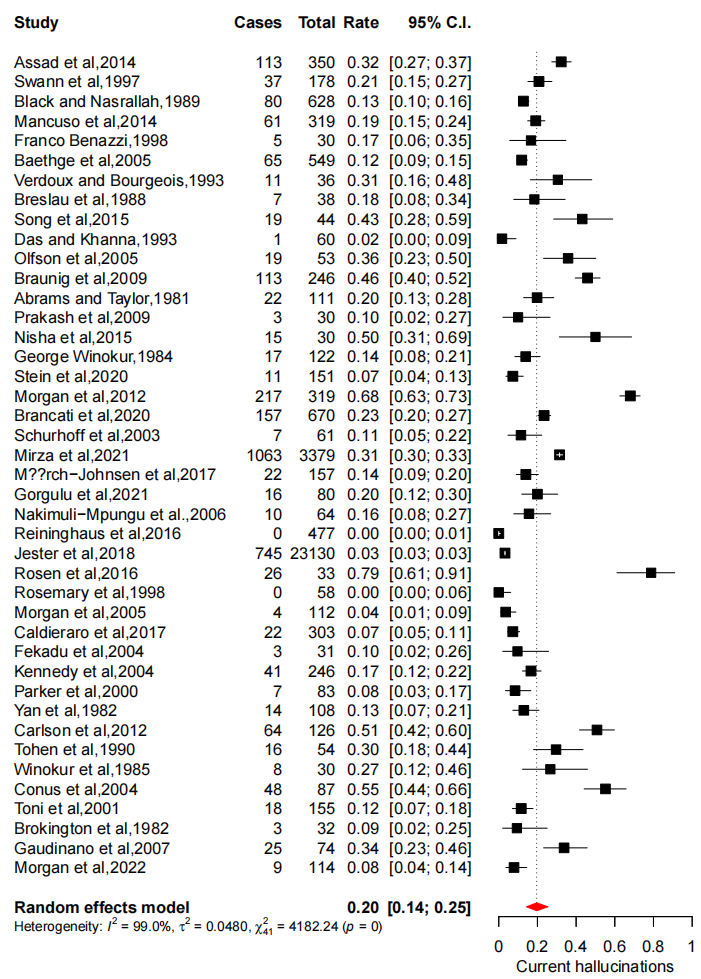


**Figure S35**

***Pooled rate of current hallucinations in bipolar patients***


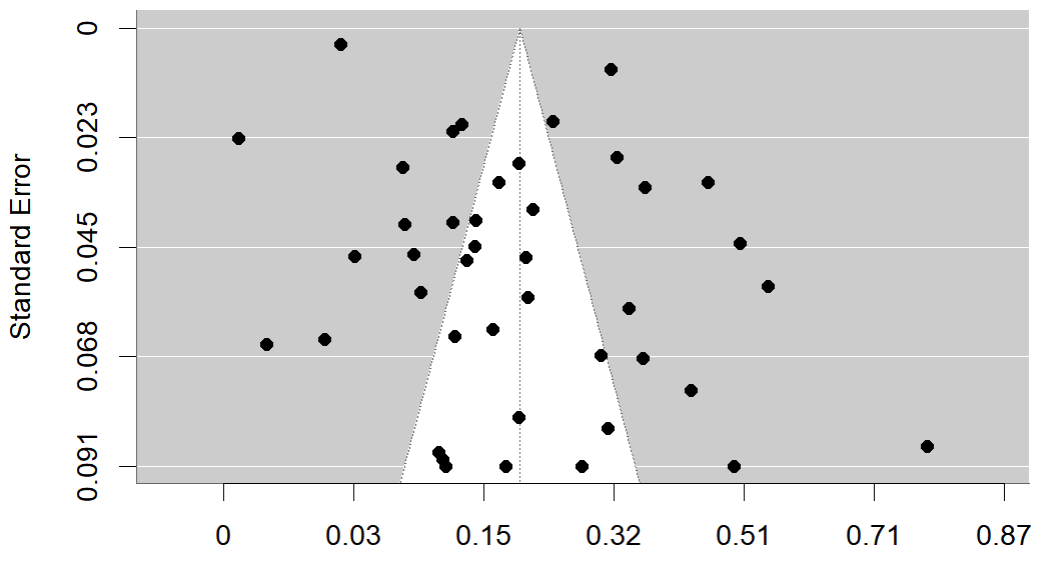

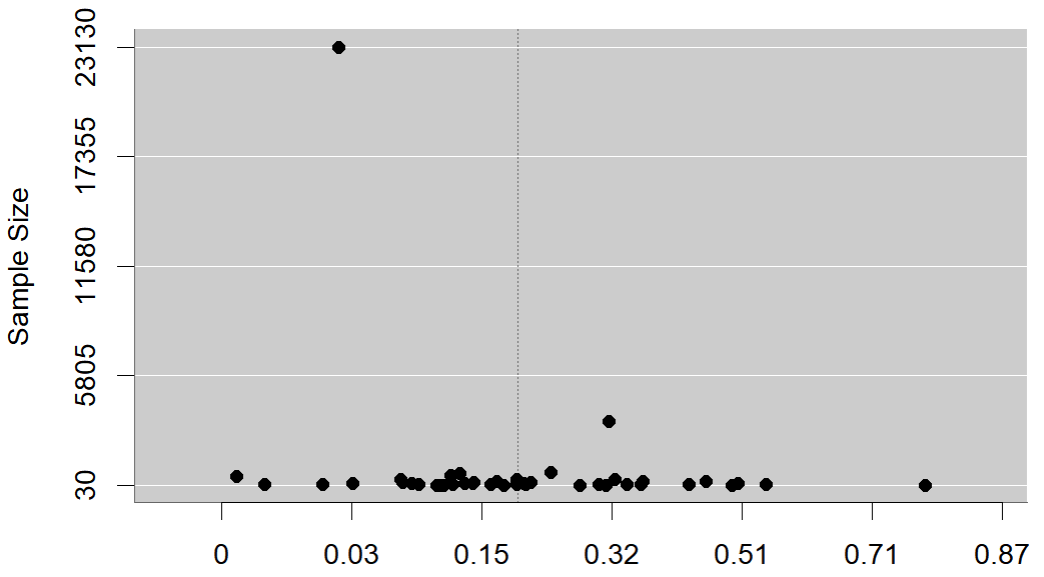


(a) original funnel (b) plot by sample size

**Figure S36**

***Funnel plots for current hallucinations in bipolar patients***


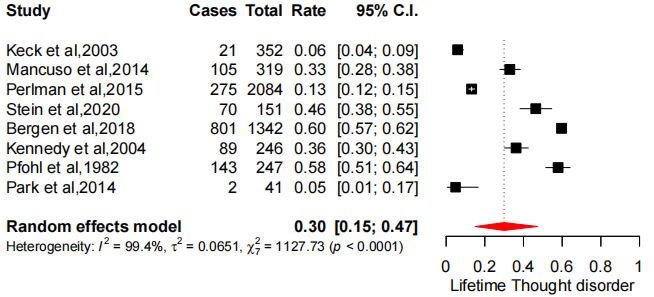


**Figure S37**

***Pooled rate of lifetime thought disorders in bipolar patients***


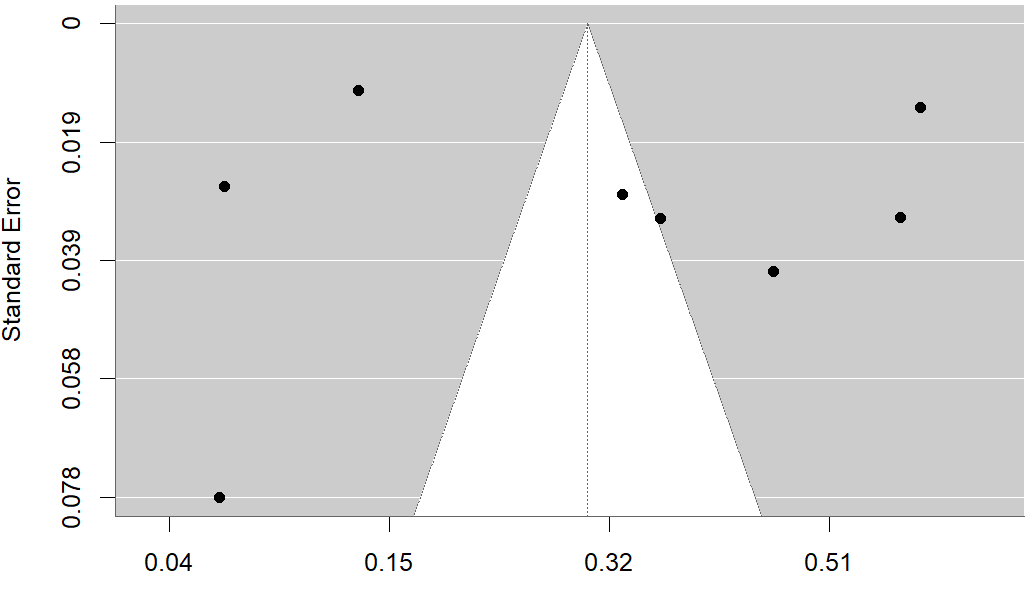

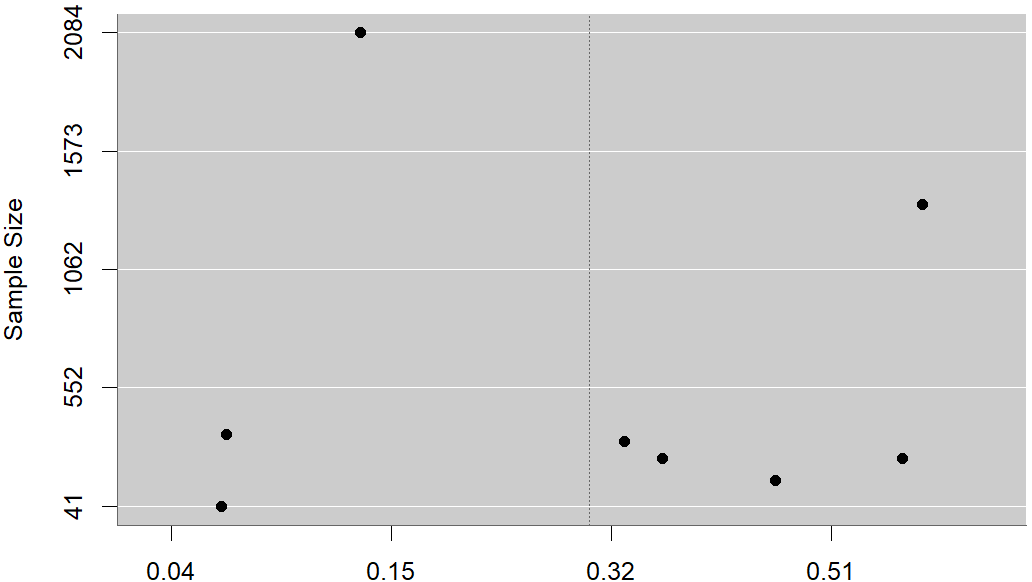


(a) original funnel (b) plot by sample size

**Figure S38**

***Funnel plots for lifetime thought disorders in bipolar patients***

***
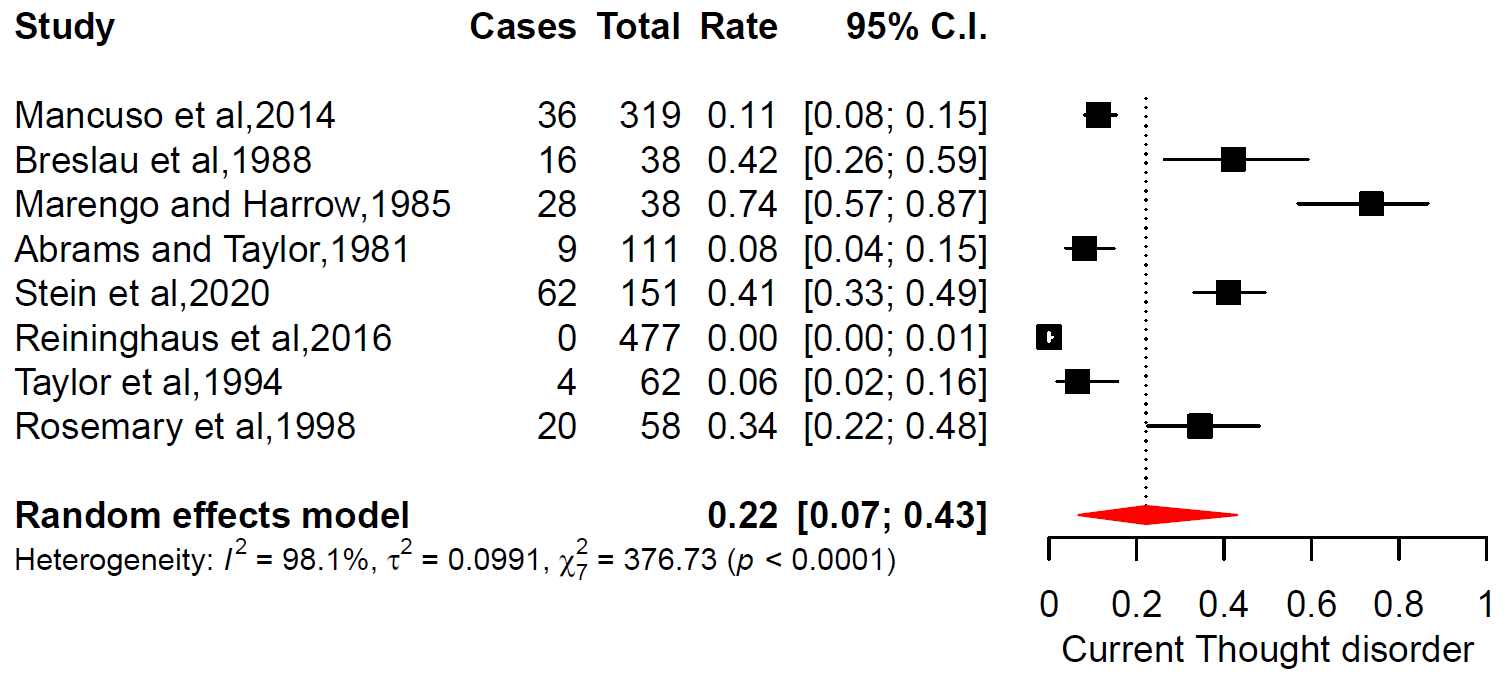
***

**Figure S39**

***Pooled rate of current thought disorders in bipolar patients***


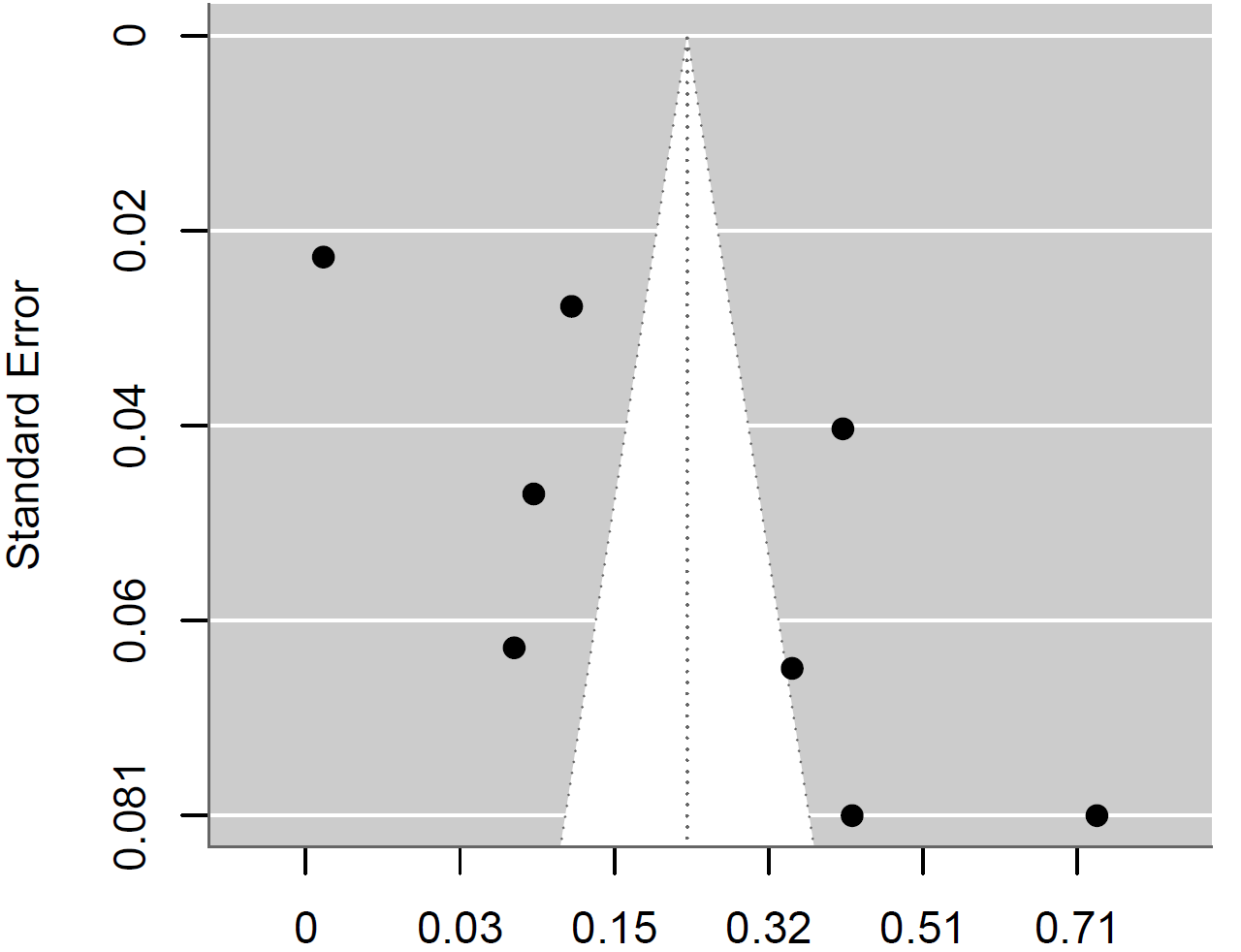

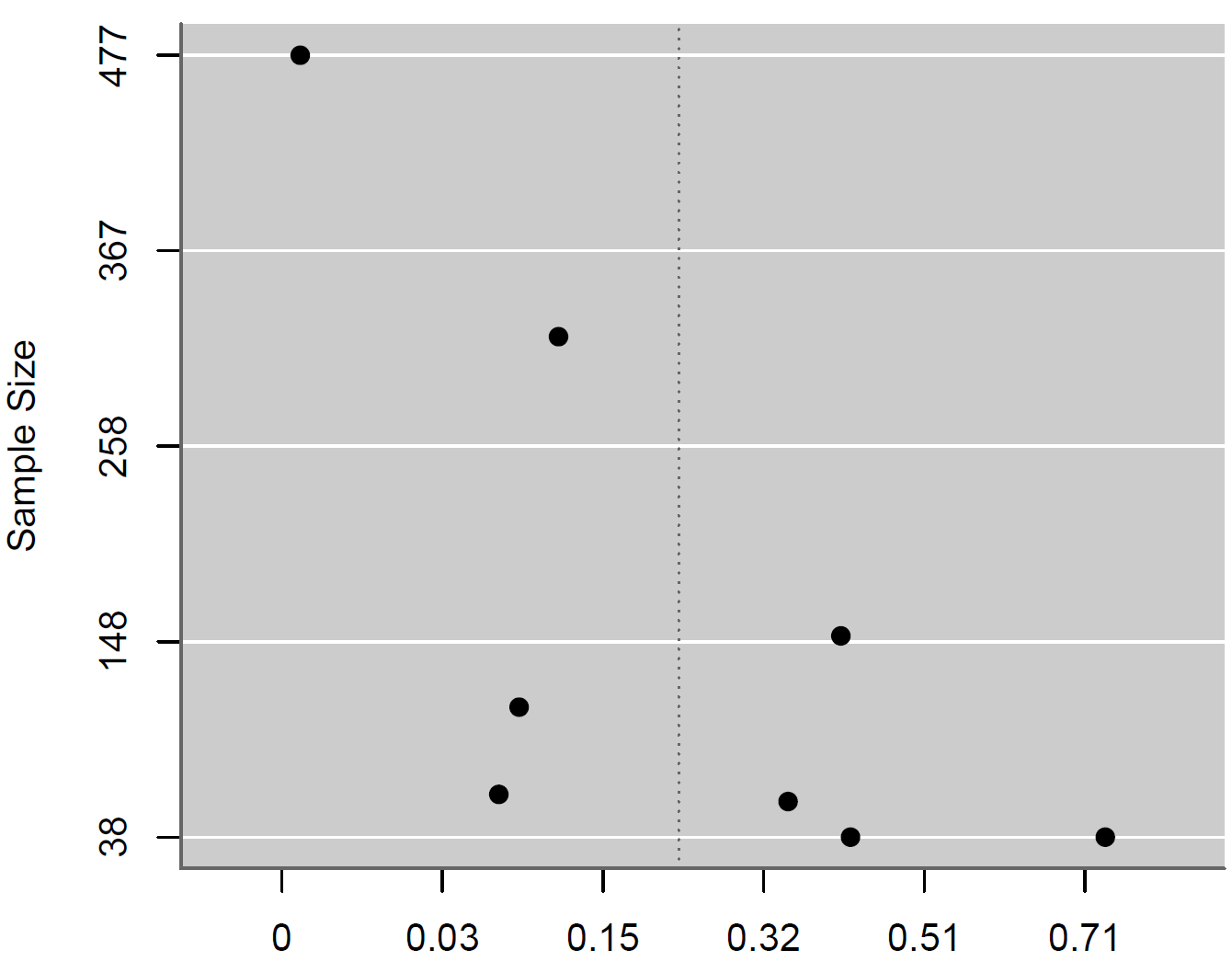


(a) original funnel (b) funnel plot by sample size

**Figure S40**

***Funnel plots for current thought disorders in bipolar patients***
