# Supplementary material for: Comorbid Schizophrenia and Psychotic Symptoms in Patients With Bipolar Disorder: A Meta‐Analysis of the Global Literature
Source: Bipolar Disord. 2026 Mar 18;28(3):e70093. doi: 10.1111/bdi.70093 (PMC12997143; doi:10.1111/bdi.70093)
Supplement: Supplementary file 2 — Table S1–S7 : Search strategies, coding methods, abbreviations, and lifetime psychosis in bipolar patients (mixed episode). [file BDI-28-0-s003.docx]

**Table S1**

***Search strategies for synthesis of comorbid schizophrenia and psychosis***

| Database | Boolean formula |
| --- | --- |
| Psych INFO | ((schizophrenia or psychosis or psychotic) and (bipolar or manic-depressive or mania) and (prevalence or proportion or rate or comorbid*)).ab. or ((schizophrenia or psychosis or psychotic) and (bipolar or manic-depressive or mania) and (prevalence or proportion or rate or comorbid*)).ti. |
| Web of Science | (AB=(schizophrenia) OR AB=(psychosis) OR AB=(psychotic)) AND (AB=(bipolar) OR AB=(mania) OR AB=(manic-depressive)) AND (AB=(prevalence) OR AB=(proportion) OR AB=(rate) OR AB=(comorbid*)) |
| MEDLINE | ((schizophrenia [Title/Abstract]) OR (psychosis [Title/Abstract]) OR (psychotic [Title/Abstract])) AND ((bipolar [Title/Abstract]) OR (manic- depressive [Title/Abstract]) OR (mania [Title/Abstract])) AND ((prevalence [Title/Abstract]) OR (proportion [Title/Abstract]) OR (rate [Title/Abstract])) |

**Table S2**

***Search strategies for synthesis of comorbid delusions***

| Database | Boolean formula |
| --- | --- |
| Psych INFO | ((schizophrenia or psychosis or psychotic) and (bipolar or manic-depressive or mania) and delu*).ab. or ((schizophrenia or psychosis or psychotic) and (bipolar or manic-depressive or mania) and delu*).ti. |
| Web of Science | (AB=(schizophrenia) OR AB=(psychosis) OR AB=(psychotic)) AND (AB=(bipolar) OR AB=(mania) OR AB=(manic-depressive)) AND AB=(delu*) |
| MEDLINE | ((schizophrenia [Title/Abstract]) OR (psychosis [Title/Abstract]) OR (psychotic [Title/Abstract])) AND ((bipolar [Title/Abstract]) OR (manic- depressive [Title/Abstract]) OR (mania [Title/Abstract])) AND (delu* [Title/Abstract]) |

**Table S3**

***Search strategies for synthesis of comorbid hallucinations***

| Database | Boolean formula |
| --- | --- |
| Psych INFO | ((schizophrenia or psychosis or psychotic) and (bipolar or manic-depressive or mania) and hallucinat*).ab. or ((schizophrenia or psychosis or psychotic) and (bipolar or manic-depressive or mania) and hallucinat*).ti. |
| Web of Science | (AB=(schizophrenia) OR AB=(psychosis) OR AB=(psychotic)) AND (AB=(bipolar) OR AB=(mania) OR AB=(manic-depressive)) AND AB=(hallucinat*) |
| MEDLINE | ((schizophrenia [Title/Abstract]) OR (psychosis [Title/Abstract]) OR (psychotic [Title/Abstract])) AND ((bipolar [Title/Abstract]) OR (manic- depressive [Title/Abstract]) OR (mania [Title/Abstract])) AND (hallucinat* [Title/Abstract]) |

**Table S4**

***Search strategies for synthesis of comorbid thought disorders***

| Database | Boolean formula |
| --- | --- |
| Psych INFO | ((schizophrenia or psychosis or psychotic) and (bipolar or manic-depressive or mania) and (thought disorder or disorganized)).ab. or ((schizophrenia or psychosis or psychotic) and (bipolar or manic-depressive or mania) and (thought disorder or disorganized)).ti. |
| Web of Science | (AB=(schizophrenia) OR AB=(psychosis) OR AB=(psychotic)) AND (AB=(bipolar) OR AB=(mania) OR AB=(manic-depressive)) AND(AB=(thought disorder) OR AB=(disorganized)) |
| MEDLINE | ((schizophrenia [Title/Abstract]) OR (psychosis [Title/Abstract]) OR (psychotic [Title/Abstract])) AND ((bipolar [Title/Abstract]) OR (manic- depressive [Title/Abstract]) OR (mania [Title/Abstract])) AND ((thought disorder [Title/Abstract]) OR (disorganized [Title/Abstract])) |

**Table S5**

***The coding method for included variables***

| **Variable name** | **Description** | **Type** | **Coding** |
| --- | --- | --- | --- |
| Gender | Sex identity of the participants | Categorical | 0=male,  1=female. |
| Age | Age at assessment | Numeric (mean or median value) | Default |
| Study IDs | In order to manage the dataset numbers were assigned to each study sequentially, from 1 to n. | Numeric | Default |
| Publication year | The year that the article has been published | Numeric | Default |
| Sampling method | The way participants have been sampled | Nominal | 0=convenient,  1=probabilistic. |
| Multicentre study | Whether the participants have been recruited via multi centre | Nominal | 0=single,  1=multicentre. |
| Study quality | Overall quality of the study, rated via the JBI checklist | Numeric | Default |
| First level evidence | Whether the patient was consecutively recruited | Nominal | 0=non-consecutive,  1=consecutive. |
| Patient type | Sampling source | Nominal | 1=outpatient, 2=mixed,  3=inpatient. |
| Clinical status | Whether patient was in an active state of the disorder | Nominal | 0= euthymic,  1= mixed,  2= acute/active. |
| Age of onset | the number of years after the first diagnosis or first episode onset of the disorder | Numeric (mean or median value) | Default |
| Bipolar Type | the type of the bipolar diagnosis | Nominal | Bipolar II Disorder=0,  Bipolar Spectrum Disorder=1.  Bipolar I Disorder=2, Psychotic Bipolar Disorder=3. |
| Manic | The rate of patient with current/ predominant manic episode | Numeric | Default |
| Depression | The rate of patient with current/ predominant depressive episode | Numeric | Default |
| Episode type | Whether the polarity is predominant? (for sub analysis of psychosis in different mood episode) | Binary | 0=recent  1=predominant |
| Mixed | The rate of patient with current/ predominant mixed episode | Numeric | Default |
| Type of delusions | whether the delusion was general or specific | Nominal | 0=specific,  1=general. |
| Type of hallucinations | whether the hallucination was general or specific | Nominal | 0=specific,  1=general. |
| Type of thought disorder | whether the thought disorder was general or specific | Nominal | 0=specific,  1=general. |
| **Outcome variables** | | | |
| Rate of comorbid schizophrenia | Whether bipolar patients had/have schizophrenia diagnosis (current/ lifetime) | Numeric | Default |
| Rate of comorbid psychosis | Whether bipolar patients had/have comorbid psychosis (current/ lifetime) | Numeric | Default |
| Rate of comorbid delusions | Whether bipolar patients had/have concurrent delusions (lifetime/ current) | Numeric | Default |
| Rate of comorbid hallucinations | Whether bipolar patients had/have concurrent hallucinations (lifetime/ current) | Numeric | Default |
| Rate of comorbid thought disorder | Whether bipolar patients had/have concurrent thought disorder (lifetime/ current) | Numeric | Default |
| **Variables not involved in the analysis** | | | |
| Diagnostic standard | The criteria used for diagnosis | Nominal | DSM, ICD, RDC etc. |
| Assessment tools | The tools used for assessment, specifically the psychiatric symptoms | Nominal | PANSS, YMRS etc. |
| Location | The place where the study was conducted | Nominal | AL ,CN, DZ etc. |

***Note*.** the lifetime or current psychosis was mainly decided by semantic descriptions**.** For instance, patient diagnosed by structured clinical interview for DSM IV as having psychotic features are considered to be suffering from a current or recent condition unless otherwise specified**;** the highest rate of a specific type of the psychotic condition was recorded in case the prevalence of general hallucinations, delusions, and thought disorder was not reported,

**Table S6**

***The Abbreviated Terms and Full Names***

| Short name | Full name |
| --- | --- |
| AMDP | Association for Methodology and Documentation in Psychiatry |
| BISS | Bipolar Inventory of Symptoms Scale |
| BPRS | Brief Psychiatric Rating Scale |
| CAPE | Community Assessment of Psychic Experiences |
| CGI | Clinical Global Impression |
| CIDI | Composite International Diagnostic Interview |
| CMBT | Comprehensive measure of bizarre-idiosyncratic thinking |
| CORE | Core system (see Parker et al., 2000) |
| DCR10 | Diagnostic criteria for research-10 (DCR10) criteria |
| DCUPR | Diagnostic Criteria for Use in Psychiatric Research |
| DI-PAD | Diagnostic Interview for Psychosis and Affective Disorders |
| DIGS | Diagnostic Interview for Genetic Studies |
| DIP | Diagnostic Interview for Psychosis |
| DIS | National Institute of Mental Health Diagnostic Interview Schedule |
| DSM | Diagnostic and Statistical Manual of Mental Disorders |
| EMA | Ecological Momentary Assessments |
| HoNOS | Health of the Nations Outcome Scale |
| ICD | International Classification of Diseases |
| MDQ | Mood Disorder Questionnaire |
| MDS | Minimum Data Set (MDS) 2.0 admission assessment |
| MINI | Mini-International Neuropsychiatric Interview |
| OPCRIT | Operational Criteria Checklist for Psychotic Illness |
| ORSM | Observer-Rated Scale for Mania |
| PANSS | Positive and Negative Syndrome Scale |
| PDI | Peters et al Delusions Inventory |
| PSE | Present State Examination |
| RDC | Research Diagnostic Criteria |
| RPMIP | Royal Park Multidiagnostic Instrument for Psychosis |
| SADS | Schedule for Affective Disorder and Schizophrenia |
| SAPS | Scale for the Assessment of Positive Symptoms |
| SID | Semi structured Interview for Depression |
| SCID | Structured Clinical Interview for DSM |
| SCAN | Schedule for Clinical Assessment in Neuropsychiatry |
| WGSQ | Wirthlin group survey questionnaire (claimed to be comparable to SCID) |
| YMRS | Young Mania Rating Scale |

**Table S7**

***Lifetime psychosis in bipolar patients with mixed episode***

| Study | Study location | Patient type | Patient number | Diagnosis | Lifetime psychosis |
| --- | --- | --- | --- | --- | --- |
| Keck et al. (2003) | USA | Outpatient | 223 Bipolar I patients | DSM4 | 67.71% (n=151) |
| Andrade-Nascimento et al. (2011) | Brazil | Outpatient | 282 Bipolar I patients | DSM4 | 47.52% (n=134) |
| Ferentinos et al. (2017) | Greece | Outpatient | 50 BSD patients | DSM | 22% (n=11) |
| Pacchiarotti et al. (2011) | Italy | Inpatient | 88 Bipolar I patients | DSM4TR | 57.95% (n=51) |

Andrade-Nascimento, M., Miranda-Scippa, Â., Nery-Fernandes, F., Kapczinski, F., & Quarantini, L. C. (2011). The identification of unipolar mania subtype based on anxiety comorbidity. *J Affect Disord*, *132*(3), 356-359. <https://doi.org/10.1016/j.jad.2011.03.005>

Ferentinos, P., Fountoulakis, K. N., Lewis, C. M., Porichi, E., Dikeos, D., Papageorgiou, C., & Douzenis, A. (2017). Validating a two-dimensional bipolar spectrum model integrating DSM-5's mixed features specifier for Major Depressive Disorder. *Comprehensive Psychiatry*, *77*, 89-99. <https://doi.org/https://doi.org/10.1016/j.comppsych.2017.06.007>

Keck, P. E., Jr., McElroy, S. L., Havens, J. R., Altshuler, L. L., Nolen, W. A., Frye, M. A., Suppes, T., Denicoff, K. D., Kupka, R., Leverich, G. S., Rush, A. J., & Post, R. M. (2003). Psychosis in bipolar disorder: phenomenology and impact on morbidity and course of illness. *Compr Psychiatry*, *44*(4), 263-269. <https://doi.org/10.1016/s0010-440x(03)00089-0>

Pacchiarotti, I., Mazzarini, L., Kotzalidis, G. D., Valentí, M., Nivoli, A. M., Sani, G., Torrent, C., Murru, A., Sanchez-Moreno, J., Patrizi, B., Girardi, P., Vieta, E., & Colom, F. (2011). Mania and depression. Mixed, not stirred. *J Affect Disord*, *133*(1-2), 105-113. <https://doi.org/10.1016/j.jad.2011.03.037>
