# Supplementary material for: Comorbid Schizophrenia and Psychotic Symptoms in Patients With Bipolar Disorder: A Meta‐Analysis of the Global Literature
Source: Bipolar Disord. 2026 Mar 18;28(3):e70093. doi: 10.1111/bdi.70093 (PMC12997143; doi:10.1111/bdi.70093)
Supplement: Supplementary file 3 — Table S8–S12: Summary tables of included studies. [file BDI-28-0-s001.doc]

Table S8

Included studies in the synthesis of comorbid schizophrenia

| Study | JBI score | Age | Criteria | Assessment | N | Pop | BD | TF | Cotype | Co (n) |
| --- | --- | --- | --- | --- | --- | --- | --- | --- | --- | --- |
| Zolezzi et al. (2017) | 4 | 41.2 | DSM | unclear | 63 | 1 | 1 | 0 | psychotic | 2 |
| Hsieh et al. (2016) | 5 | 25.1 | DSM4 | SCID | 35 | 2 | 1 | 0 | scz | 0 |
| Hsu et al. (2015) | 7 | 40.2 | ICD9 | unclear | 21060 | 2 | 1 | 0 | scz | 1116 |
| Jorgensen et al. (2018) | 7 | NA | ICD | unclear | 10560 | 1 | 1 | 1 | scz | 681 |
| Patterson-Lomba et al. (2019) | 6 | 39.6 | ICD | unclear | 69736 | 2 | 1 | 0 | psychotic | 3013 |
| Almeida and Fenner (2002) | 6 | 34.4 | ICD9 | unclear | 6182 | 2 | 1 | 0 | psychotic | 91 |
| Palzes et al. (2020) | 5 | 46 | ICD | unclear | 21150 | 2 | 1 | 0 | scz | 371 |
| Abu Nazel et al. (2017) | 5 | 37.9 | DSM4 | unclear | 49 | 1 | 1 | 0 | ssd | 11 |
| Kamat et al. (2008) | 5 | NA | MDQ | MDQ | 94 | 2 | 1 | 1 | psychotic | 9 |
| (Goodwin et al., 2002) | 5 | NA | DSM3 | SCID | 33 | 1 | 1 | 0 | scz | 6 |
| Morgan et al. (2012) | 5 | NA | DSM4 | SCID-P | 231 | 2 | 3 | 0 | sca | 8 |
| Lev-Ran et al. (2013) | 7 | NA | DSM4 | unclear | 1905 | 1 | 1 | 0 | psychotic | 63 |
| Allardyce et al. (2018); | 6 | 46 | RDC | SCAN | 4339 | 2 | 1 | 0 | sca | 356 |
| Kupfer et al. (2005); Niitsu et al. (2015) | 6 | 40.78 | DSM4 | SCID | 2718 | 2 | 1 | 0 | sca | 248 |
| Okasha et al. (2023) | 8 | 34.4 | DSM4 | SCID | 300 | 2 | 2 | 1 | scz | 22 |
| Ng et al. (2021) | 5 | NA | ICD9 | unclear | 53434 | 2 | 1 | 0 | scz | 7844 |
| Almeida et al. (2019); | 5 | 72.5 | ICD | unclear | 302 | 2 | 1 | 1 | psychotic | 51 |
| Kendler et al. (2023) | 7 | 36.4 | ICD10 | unclear | 13530 | 3 | 2 | 0 | scz | 325 |
| Bora et al. (2007) | 5 | 37.97 | DSM4 | SCID | 65 | 2 | 2 | 1 | frs | 2 |
| Tohen et al. (1992) | 6 | 30 | DSM3 | DIS | 54 | 3 | 3 | 0 | frs | 11 |
| Das and Khanna (1993) | 5 | 31.93 | DSM3R | unclear | 60 | 3 | 2 | 0 | frs | 2 |
| Sethi and Khanna (1993) | 6 | 27.7 | DSM3R | PSE | 100 | 2 | 2 | 0 | frs | 15 |
| Gorgulu et al. (2021) | 5 | 41.94 | DSM5 | unclear | 80 | 2 | 2 | 0 | frs | 7 |
| Gonzalez-Pinto et al. (2003) | 5 | 36.9 | DSM4 | SAPS | 103 | 3 | 2 | 0 | frs | 23 |
| Yan et al. (1982) | 6 | 31.51 | mixed | unclear | 108 | 3 | 2 | 0 | frs | 4 |
| Carlson et al. (2012) | 6 | 29.3 | DSM3R | SCID | 126 | 3 | 3 | 0 | frs | 35 |
| Kennedy et al. (2004) | 4 | NA | DSM4 | OPCRIT | 246 | 2 | 2 | 0 | frs | 30 |

*Note*. The JBI score refers to the study quality score rated by the JBI checklist (range = 0-9). Pop denotes the patient population (1 = outpatient, 2 = mixed, 3 = inpatient). Criteria specify the diagnostic criteria used, while Assessment refers to the interview tools employed (See Table S6 for details). BD represents the bipolar diagnosis subtype (0 = BD II, 1 = BSD, 2 = BD I, 3 = Psychotic BD). TF indicates the timeframe (0 = current, 1 = lifetime). Cotype refers to the comorbidity type (psychotic = psychotic disorders, scz = schizophrenia, sca = schizoaffective disorders, frs = first rank symptoms). Co (n): number of patients with this condition.

Reference:

**Table S9**

Included studies in the synthesis of comorbid psychosis

| Study | JBI score | Age | Criteria | Assessment | N | Pop | BD | TF | Co (n) | Co MC (n) | Co MIC (n) | |
| --- | --- | --- | --- | --- | --- | --- | --- | --- | --- | --- | --- | --- |
| Asaad et al. (2014) | 7 | 34.65 | DSM4 | SCID | 350 | unclear | 1 | 0 | 192 | 149 | 43 | |
| Keck et al. (2003) | 7 | 41.32 | DSM4 | SCID-P | 352 | 1 | 2 | 2 | 238 | 136 | 102 | |
| Chan et al. (2019) | 6 | 46.95 | DSM5 | SCID | 104 | 1 | 1 | 2 | 56 | NA | NA | |
| Ospina et al. (2016) | 5 | 47.22 | DSM4 | SCID | 105 | unclear | 1 | 2 | 43 | NA | NA | |
| Braga et al. (2012) | 7 | 36.7 | DSM4 | SCID | 200 | unclear | 2 | 2 | 143 | NA | NA | |
| Andrade-Nascimento et al. (2011) | 7 | 41.6 | DSM4 | SCID | 298 | 1 | 2 | 2 | 144 | NA | NA | |
| Kauer-Sant'Anna et al. (2009) | 6 | 22.19 | DSM4 | MINI | 53 | 1 | 2 | 0 | 8 | NA | NA | |
| van Os et al. (2007) | 7 | 44.6 | mixed | CGI | 3459 | 2 | 2 | 0 | 1849 | NA | NA | |
| Kilbourne et al. (2005) | 5 | 46.6 | DSM4 | SCID | 330 | 3 | 1 | 0 | 112 | NA | NA | |
| Kessing (2004) | 6 | NA | ICD10 | unclear | 1719 | 2 | 1 | 0 | 908 | NA | NA | |
| (Frangou, 2002) | 5 | 42 | DSM4 | SCID | 63 | unclear | 2 | 2 | 39 | NA | NA |  |
| Ballester et al. (2014) | 5 | 38.99 | DSM4 | SCID | 227 | 2 | 1 | 0 | 24 | NA | NA | |
| Cardoso et al. (2008) | 6 | 42.8 | DSM4 | SCID | 178 | 1 | 1 | 2 | 83 | NA | NA | |
| Dalkner et al. (2021) | 6 | 42.41 | DSM4 | SCID | 148 | 1 | 1 | 2 | 85 | NA | NA | |
| Janicak et al. (1998) | 5 | 36.2 | DSM3R | unclear | 32 | 3 | 2 | 0 | 23 | NA | NA | |
| Medici et al. (2020) | 4 | 41.24 | ICD10 | unclear | 46 | 3 | 2 | 0 | 21 | NA | NA | |
| Frye et al. (2015) | 6 | 42.6 | DSM4 | SCID | 1363 | 2 | 1 | 2 | 563 | NA | NA | |
| Levy et al. (2011) | 6 | 38.8 | DSM4 | SCID | 82 | 3 | 2 | 0 | 39 | NA | NA | |
| Yildiz and Sachs (2003) | 5 | NA | DSM4 | SCID | 328 | 1 | 1 | 2 | 138 | NA | NA | |
| Finseth et al. (2012) | 7 | 42.2 | DSM4 | SCID | 206 | 3 | 1 | 2 | 96 | NA | NA | |
| Ernst and Goldberg (2004) | 5 | 40.8 | DSM4 | SCID | 43 | unclear | 1 | 2 | 38 | NA | NA | |
| Nery et al. (2014) | 8 | 40.6 | DSM4 | SCID | 483 | 1 | 1 | 2 | 352 | NA | NA | |
| Costa et al. (2020) | 6 | NA | DSM4 | SCID | 4032 | 1 | 1 | 2 | 1536 | NA | NA | |
| Treuer et al. (2007) | 5 | 34.45 | DSM4TR | unclear | 894 | 2 | 1 | 0 | 552 | NA | NA | |
| Sachs et al. (2002) | 4 | NA | DSM4 | CGI | 156 | 3 | 2 | 0 | 61 | NA | NA | |
| Fridberg et al. (2009) | 5 | 42.77 | DSM4 | SCID | 68 | 2 | 2 | 2 | 26 | NA | NA | |
| Nehme et al. (2018) | 5 | 38.98 | DSM5 | SCID | 40 | 2 | 2 | 0 | 18 | NA | NA | |
| Serretti et al. (2002) | 7 | 44.36 | DSM | OPCRIT | 576 | 2 | 1 | 2 | 362 | NA | NA | |
| Swann et al. (1997) | 4 | 39 | DSM3 | SADS | 178 | 3 | 2 | 0 | 30 | NA | NA | |
| Gao et al. (2008) | 6 | 36.09 | DSM4 | SCID-P | 144 | 2 | 1 | 2 | 59 | NA | NA | |
| Nivoli et al. (2013) | 6 | 43.58 | DSM4TR | SCID | 604 | 1 | 1 | 2 | 314 | NA | NA | |
| Heron et al. (2003) | 6 | 45 | DSM4 | SCAN | 92 | unclear | 1 | 2 | 60 | NA | NA | |
| Chen et al. (2021) | 5 | 38.32 | DSM4 | SCID | 94 | 2 | 1 | 2 | 54 | NA | NA | |
| Salem et al. (2019) | 6 | 33.04 | DSM4TR | SCID | 507 | 3 | 1 | 0 | 396 | NA | NA | |
| Burton et al. (2018) | 6 | 40.9 | DSM4 | DIGS | 381 | unclear | 1 | 2 | 213 | NA | NA | |
| Cremaschi et al. (2017) | 6 | 48.6 | DSM4TR | SCID | 362 | 2 | 1 | 2 | 208 | NA | NA | |
| Raymont et al. (2003) | 5 | 42 | DSM4 | SCID | 63 | unclear | 2 | 2 | 39 | NA | NA | |
| Black and Nasrallah (1989) | 5 | NA | DSM3 | unclear | 628 | 3 | 1 | 0 | 249 | NA | NA | |
| Upthegrove et al. (2015) | 6 | 47 | DSM4 | SCAN | 2019 | unclear | 2 | 2 | 1408 | NA | NA | |
| Garcia-Gonzalez et al. (2020) | 5 | NA | ICD10 | CIDI | 781 | unclear | 1 | 2 | 358 | NA | NA | |
| Tost et al. (2010) | 6 | 42.4 | DSM4 | SCID | 42 | 3 | 2 | 2 | 30 | NA | NA | |
| Braunig et al. (2009) | 6 | 41.27 | DSM4 | AMDP | 246 | unclear | 2 | 0 | 155 | NA | NA | |
| Abrams and Taylor (1981) | 6 | 40.32 | feighner | unclear | 111 | 3 | 2 | 0 | 69 | NA | NA | |
| Schulze et al. (2005) | 7 | 44.58 | DSM4 | mixed | 540 | unclear | 1 | 2 | 294 | NA | NA | |
| Perlman et al. (2016) | 5 | 44.35 | DSM4 | DI-PAD | 2084 | unclear | 1 | 2 | 1414 | NA | NA | |
| Nisha et al. (2015) | 6 | 39.1 | DCR10 | unclear | 30 | 3 | 1 | 0 | 24 | NA | NA | |
| Schurhoff et al. (2003) | 6 | 41.14 | DSM4 | PDI | 61 | 3 | 1 | 0 | 31 | NA | NA | |
| Song et al. (2012) | 6 | 32.2 | DSM4 | DIGS | 212 | unclear | 1 | 0 | 27 | NA | NA | |
| Ekman et al. (2017) | 7 | 38.47 | DSM4TR | mixed | 167 | 1 | 1 | 2 | 85 | NA | NA | |
| Zenisek et al. (2015) | 4 | 35.89 | DSM4 | SCID | 58 | 1 | 1 | 2 | 30 | NA | NA | |
| De Pradier et al. (2010) | 4 | 49.65 | DSM4 | DIGS | 137 | 1 | 1 | 2 | 74 | NA | NA | |
| Ferentinos et al. (2017) | 5 | 48.17 | DSM | MINI | 123 | 1 | 1 | 2 | 62 | NA | NA | |
| Mallet et al. (2022) | 7 | 40.39 | DSM4TR | SCID | 2174 | 1 | 1 | 2 | 723 | NA | NA | |
| Church et al. (2017) | 6 | 30.4 | DSM4 | SCID | 159 | 2 | 2 | 2 | 110 | NA | NA | |
| Ortiz et al. (2021) | 5 | 44.7 | DSM5 | SADS | 53 | 1 | 1 | 2 | 17 | NA | NA | |
| Azorin et al. (2013) | 6 | 43 | DSM4 | SCID | 1090 | 3 | 2 | 0 | 521 | 364 | 180 | |
| Dilsaver et al. (1997) | 6 | 33.33 | DSM | SADS | 129 | 3 | 1 | 0 | 97 | NA | NA | |
| Cakir et al. (2016) | 5 | 40.6 | DSM4 | SCID | 135 | 1 | 2 | 0 | 105 | NA | NA | |
| Azorin et al. (2015) | 6 | 45.54 | DSM4 | SCID-P | 228 | unclear | 1 | 0 | 62 | NA | NA | |
| Caldieraro et al. (2017) | 5 | 39.45 | DSM4TR | BISS | 303 | 1 | 1 | 0 | 32 | NA | NA | |
| Karakus and Tamam (2011) | 7 | 33.87 | DSM4 | SCID | 124 | 1 | 2 | 0 | 82 | NA | NA | |
| Severance et al. (2014) | 5 | 36.21 | DSM4 | SCID | 264 | 2 | 1 | 1 | 38; 208 | NA | NA | |
| Weinstock et al. (2016) | 5 | 42.2 | DSM4TR | SCID | 230 | 2 | 2 | 0 | 105 | NA | NA | |
| Shashidhara et al. (2015) | 8 | 31.63 | DSM4TR | SCID | 396 | 3 | 2 | 1 | 205; 235 | NA | NA | |
| Belete et al. (2016) | 7 | 32 | DSM4 | unclear | 411 | 1 | 1 | 0 | 188 | NA | NA | |
| Belteczki et al. (2018) | 5 | NA | ICD10 | unclear | 365 | 3 | 1 | 1 | 89; 285 | NA | NA | |
| van Bergen et al. (2019) | 7 | 49.5 | DSM4 | SCID | 1342 | 2 | 2 | 2 | 990 | NA | 404 | |
| Fekadu et al. (2004) | 6 | 27 | ICD10 | SCAN | 31 | 1 | 1 | 0 | 7 | NA | NA | |
| Kennedy et al. (2004) | 4 | NA | DSM4 | OPCRIT | 246 | 2 | 2 | 0 | 165 | NA | 73 | |
| Benazzi (2003) | 7 | 41.7 | DSM4 | SCID-C | 260 | 1 | 0 | 0 | 22 | NA | NA | |
| Neves et al. (2009) | 7 | 42.69 | DSM4 | MINI | 168 | 2 | 1 | 2 | 32 | NA | NA | |
| Moon et al. (2012) | 5 | 39.4 | DSM4 | unclear | 275 | 1 | 1 | 2 | 73 | NA | NA | |
| Keramatian et al. (2022) | 5 | 42 | DSM4 | MINI | 319 | 2 | 1 | 2 | 143 | NA | NA | |
| Ozyildirim et al. (2010) | 5 | NA | DSM4 | unclear | 281 | 2 | 2 | 1 | 43; 215 | NA | NA | |
| Baek et al. (2011) | 5 | 37.59 | DSM4TR | mixed | 307 | unclear | 1 | 2 | 149 | NA | NA | |
| Dilsaver et al. (2008) | 6 | 34.9 | DSM4 | SCID-C | 69 | 1 | 1 | 2 | 47 | NA | NA | |
| Pacchiarotti et al. (2011) | 7 | 38.77 | DSM4TR | SCID | 134 | 3 | 2 | 2 | 80 | NA | NA | |
| Musetti et al. (2018) | 7 | 38.6 | DSM4 | unclear | 234 | 1 | 1 | 2 | 99 | NA | NA | |
| Mantere et al. (2004) | 7 | 37.7 | DSM4 | SCID | 199 | 2 | 1 | 1 | 31, 95 | NA | NA | |
| McGrady et al. (2017) | 6 | 42.8 | DSM4TR | unclear | 121 | 1 | 1 | 2 | 59 | NA | NA | |
| Samellas et al. (2004) | 6 | 40 | DSM4 | unclear | 74 | 3 | 2 | 0 | 47 | NA | NA | |
| Dilsaver et al. (1994) | 5 | 34.12 | RDC | SADS | 75 | 3 | 2 | 0 | 65 | NA | NA | |
| Rybakowski et al. (2007) | 7 | NA | DSM4 | unclear | 539 | 1 | 1 | 0 | 119 | NA | NA | |
| Kaymaz et al. (2006) | 6 | 41.2 | DSM3R | CIDI | 132 | unclear | 1 | 2 | 15 | NA | NA | |
| Akiskal and Benazzi (2005) | 7 | 41.3 | DSM4 | SCID-C | 374 | 1 | 0 | 0 | 29 | NA | NA | |
| Pavlova et al. (2018) | 6 | 41.79 | DSM4TR | MINI | 174 | unclear | 1 | 0 | 37 | NA | NA | |
| Dell'Osso et al. (2015) | 6 | 35.6 | DSM4 | SCID | 503 | 1 | 1 | 2 | 193 | NA | NA | |
| Sato et al. (2005) | 6 | 48.64 | ICD10 | AMDP | 95 | 3 | 1 | 0 | 17 | NA | NA | |
| Perugi et al. (2000) | 7 | 38.4 | DSM3R | SID | 320 | 3 | 2 | 0 | 195 | 118 | 77 | |
| Cho et al. (2020) | 6 | 36.7 | DSM4 | SCID-C | 71 | 3 | 1 | 0 | 35 | NA | NA | |
| Miller et al. (2014) | 6 | 50.3 | ICD9 | unclear | 170173 | 2 | 1 | 0 | 22119 | NA | NA | |
| Altindag et al. (2006) | 5 | 43.54 | DSM4 | SCID | 70 | 1 | 2 | 0 | 44 | NA | NA | |
| Barata et al. (2020) | 4 | 43 | ICD10 | unclear | 218 | 3 | 1 | 0 | 95 | NA | NA | |
| Corvin et al. (2001) | 5 | 44 | ICD10 | SCAN | 92 | unclear | 1 | 2 | 64 | NA | NA | |
| Aedo et al. (2018) | 6 | 37.6 | DSM4TR | SCID | 235 | 1 | 1 | 0 | 90 | NA | NA | |
| Buoli et al. (2017) | 5 | 51.65 | DSM5 | unclear | 238 | unclear | 1 | 2 | 171 | NA | NA | |
| Ragazan et al. (2019) | 7 | 44.2 | ICD10 | unclear | 16271 | 3 | 1 | 0 | 1692 | NA | NA | |
| Huang et al. (2015) | 8 | 31 | ICD10 | MINI | 2613 | 2 | 1 | 0 | 628 | NA | NA | |
| Tohen, Goldberg, et al. (2003) | 6 | 40 | DSM4 | SCID-P | 453 | 2 | 2 | 0 | 260 | NA | NA | |
| Lee et al. (2015) | 6 | 22.8 | DSM4TR | SCID | 61 | unclear | 1 | 0 | 16 | NA | NA | |
| Keck et al. (2009) | 5 | 39.67 | DSM4TR | MINI | 480 | 3 | 2 | 0 | 111 | NA | NA | |
| Zhang et al. (2013) | 6 | 36 | ICD10 | unclear | 175 | 2 | 1 | 0 | 73 | NA | NA | |
| Choi et al. (2010) | 6 | 33.7 | DSM4 | SCID | 103 | 3 | 2 | 0 | 78 | NA | NA | |
| Shan et al. (2016) | 5 | 41.98 | unclear | unclear | 47 | unclear | 1 | 0 | 19 | NA | NA | |
| Basterreche et al. (2008) | 5 | 44 | DSM4 | SCID | 160 | 3 | 2 | 0 | 134 | 23 | 111 | |
| Xiang et al. (2013) | 6 | 35.5 | DSM4 | MINI | 309 | 2 | 1 | 0 | 100 | NA | NA | |
| Bulbul et al. (2020) | 5 | 38 | DSM5 | unclear | 101 | 1 | 1 | 0 | 54 | NA | NA | |
| Mazaheri-Tehrani et al. (2014) | 4 | 36.2 | DSM4 | unclear | 64 | unclear | 1 | 0 | 31 | NA | NA | |
| Yildiz et al. (2008) | 5 | NA | DSM4 | SCID | 66 | 3 | 2 | 0 | 44 | NA | NA | |
| Mrad et al. (2016) | 6 | 35.2 | DSM4TR | unclear | 92 | 1 | 2 | 0 | 42 | NA | NA | |
| Scott et al. (2017) | 6 | 46.7 | DSM4 | DIGS | 298 | unclear | 1 | 2 | 154 | NA | NA | |
| Menculini et al. (2022) | 7 | 45.39 | DSM5 | SCID-C | 161 | 2 | 1 | 2 | 71 | NA | NA | |
| Hirschfeld et al. (2004) | 4 | 38.78 | DSM4 | SCID | 259 | 3 | 2 | 0 | 110 | NA | NA | |
| Khanna et al. (2005) | 4 | 35.1 | DSM4 | unclear | 290 | 3 | 2 | 0 | 118 | NA | NA | |
| van der Werf-Eldering et al. (2011) | 5 | 45.5 | DSM4 | PANSS | 85 | unclear | 1 | 2 | 36 | NA | NA | |
| Berkol et al. (2016) | 5 | 40.8 | DSM4 | SCID | 200 | 1 | 1 | 0 | 154 | NA | NA | |
| Yazici et al. (2002) | 5 | 39.29 | DSM4 | unclear | 272 | unclear | 2 | 0 | 200 | 105 | 95 | |
| Wang et al. (2021) | 8 | 37.33 | ICD10 | MINI | 742 | 3 | 1 | 0 | 312 | NA | NA | |
| Yen et al. (2008) | 7 | 40.23 | DSM4 | SCID | 59 | 1 | 1 | 0 | 17 | NA | NA | |
| Kruger et al. (2010) | 4 | 37.4 | DSM4 | ORSM | 31 | 3 | 2 | 0 | 12 | NA | NA | |
| Serretti et al. (2011) | 5 | 36.35 | DSN4 | MINI | 132 | 3 | 1 | 0 | 57 | NA | NA | |
| Lim et al. (2001) | 5 | 57 | ICD9 | unclear | 1471 | 3 | 2 | 0 | 227 | NA | NA | |
| Lipkovich et al. (2008) | 5 | 40.55 | DSM4 | SCID-P | 251 | 3 | 2 | 0 | 114 | NA | NA | |
| Parker et al. (2013) | 6 | 35.73 | DSM4 | mixed | 632 | unclear | 1 | 2 | 136 | NA | NA | |
| Chaudhary et al. (2021) | 4 | NA | DSM4TR | SCID | 188 | 3 | 1 | 0 | 92 | 86 | 6 | |
| Kato et al. (2020) | 7 | 50.53 | ICD10 | unclear | 2609 | 1 | 1 | 0 | 147 | NA | NA | |
| Maj et al. (1998) | 4 | 41.12 | RDC | SADS | 247 | unclear | 2 | 0 | 59 | NA | NA | |
| Zutshi et al. (2006) | 6 | 30.06 | DSM4 | SCID-C | 80 | unclear | 1 | 2 | 66 | NA | NA | |
| Samalin et al. (2014) | 6 | 47.8 | DSM4 | unclear | 525 | 1 | 1 | 0 | 141 | NA | NA | |
| Schurhoff et al. (2000) | 3 | 44.29 | mixed | DIGS | 97 | unclear | 1 | 0 | 37 | NA | NA | |
| Perugi et al. (1997) | 7 | 38.2 | DSM3R | SID | 261 | 3 | 1 | 0 | 180 | 114 | 137 | |
| Chrobak et al. (2021) | 5 | 42 | DSM5 | unclear | 33 | unclear | 1 | 2 | 10 | NA | NA | |
| Larsen et al. (2019) | 4 | 38.3 | DSM5 | SCID | 62 | unclear | 1 | 2 | 31 | NA | NA | |
| Carugno et al. (2021) | 5 | 45.5 | DSM | SCID | 414 | 3 | 2 | 0 | 226 | NA | NA | |
| Tohen, Zarate, et al. (2003) | 7 | 32.5 | DSM3R | SCID-P | 166 | 3 | 2 | 0 | 147 | 72 | 74 | |
| Parker et al. (2000) | 4 | 46.3 | DSM | CORE | 83 | 2 | 1 | 0 | 16 | NA | NA | |
| Soeiro-de-Souza et al. (2013) | 4 | 26.8 | DSM4TR | SCID | 50 | 2 | 2 | 2 | 25 | NA | NA | |
| Garcia-Jimenez et al. (2020) | 6 | 48.17 | DSM4 | SCID | 108 | 1 | 1 | 2 | 40 | NA | NA | |
| Simonsen et al. (2010) | 7 | 36.51 | DSM4 | SCID | 120 | 2 | 1 | 2 | 64 | NA | NA | |
| Serafini et al. (2018) | 6 | 52.1 | DSM | MINI | 255 | 1 | 1 | 2 | 133 | NA | NA | |
| Suppes et al. (2008) | 6 | 39.59 | DSM3R | PANSS | 516 | 3 | 2 | 0 | 99 | NA | NA | |
| Peh and Tay (2008) | 5 | NA | DSM4TR | unclear | 121 | 1 | 1 | 0 | 30 | NA | NA | |
| Tohen et al. (2014) | 6 | 39.32 | DSM | unclear | 1214 | 3 | 2 | 0 | 117 | NA | NA | |
| Kesebir et al. (2012) | 6 | 39.2 | DSM3R | SCID | 100 | 1 | 1 | 0 | 31 | NA | NA | |
| Murru et al. (2015) | 5 | 52.73 | DSM4TR | unclear | 119 | 1 | 1 | 2 | 27 | NA | NA | |
| Musetti et al. (2018) | 7 | 39.3 | DSM4 | SCID-P | 407 | 1 | 1 | 0 | 104 | NA | NA | |
| Wylie et al. (1999) | 6 | 71.7 | DSM3R | unclear | 62 | 3 | 1 | 0 | 36 | NA | NA | |
| McGrath et al. (2016) | 7 | NA | DSM4 | CIDI | 1212 | unclear | 1 | 2 | 196 | NA | NA | |
| McElroy et al. (1997) | 7 | 28 | DSM3R | SCID-P | 88 | 3 | 1 | 0 | 80 | NA | NA | |
| Mathieu et al. (2014) | 5 | 42.37 | DSM4 | DIGS | 310 | 1 | 1 | 2 | 71 | NA | NA | |
| Dautzenberg et al. (2016) | 5 | 68.5 | DSM4TR | MINI | 78 | unclear | 1 | 0 | 9 | NA | NA | |
| Newport et al. (2012) | 6 | 32 | DSM4 | SCID | 141 | unclear | 1 | 2 | 60 | NA | NA | |
| Navarro et al. (2016) | 7 | 49.2 | ICD10 | unclear | 76 | 1 | 1 | 2 | 52 | NA | NA | |
| Gopal et al. (2007) | 5 | 35.22 | DSM4 | unclear | 291 | 3 | 2 | 0 | 170 | NA | NA | |
| Kim et al. (2020) | 5 | NA | ICD10 | unclear | 12376 | unclear | 1 | 0 | 1255 | NA | NA | |
| Benard et al. (2020) | 5 | NA | ICD10 | MINI | 148 | unclear | 2 | 2 | 101 | NA | NA | |
| Grover et al. (2021) | 6 | 45.66 | DSM4 | MINI | 773 | 1 | 1 | 2 | 326 | NA | NA | |
| Lavagnino et al. (2015) | 5 | 39 | DSM4TR | SCID | 41 | 1 | 2 | 2 | 16 | NA | NA | |
| Wang et al. (2022) | 7 | 39.64 | ICD10 | unclear | 1393 | unclear | 1 | 0 | 621 | NA | NA | |
| Szmulewicz et al. (2020) | 5 | 44.1 | DSM4 | SCID | 212 | unclear | 1 | 2 | 87 | NA | NA | |
| Lloyd et al. (2005) | 4 | NA | ICD10 | SCAN | 75 | 2 | 1 | 2 | 47 | NA | NA | |
| Anyayo et al. (2021) | 5 | 37.2 | unclear | unclear | 169 | 1 | 1 | 2 | 89 | NA | NA | |
| Strakowski et al. (2000) | 7 | 27 | DSM4 | SCID-P | 50 | 3 | 2 | 2 | 43 | 18 | 25 | |
| Bauer et al. (1997) | 5 | 50 | DSM | unclear | 103 | 2 | 1 | 2 | 66 | NA | NA | |
| Calkin et al. (2009) | 5 | 44 | DSM4 | unclear | 276 | unclear | 1 | 2 | 146 | NA | NA | |
| Bora et al. (2016) | 6 | 36.2 | DSM4 | SCID | 556 | 1 | 1 | 2 | 444 | NA | NA | |
| Bond et al. (2017) | 5 | 22.8 | unclear | MINI | 80 | 2 | 1 | 2 | 57 | NA | NA | |
| Schoeyen et al. (2011) | 7 | 42 | DSM4 | SCID | 257 | 2 | 1 | 2 | 151 | NA | NA | |
| Bauer et al. (1994) | 5 | 38.55 | mixed | unclear | 239 | 2 | 1 | 0 | 105 | NA | NA | |
| De Dios et al. (2012) | 7 | 48.6 | DSM4TR | MINI | 225 | 1 | 1 | 2 | 127 | NA | NA | |
| Olincy and Martin (2005) | 4 | 45.12 | DSM4 | SCID | 42 | 1 | 2 | 2 | 29 | NA | NA | |
| Ancin et al. (2013) | 4 | 42.9 | DSM4 | SCID-P | 148 | unclear | 1 | 2 | 93 | NA | NA | |
| Angst et al. (2004) | 5 | 65.27 | mixed | unclear | 220 | 2 | 1 | 2 | 152 | 23 | 129 | |
| Anticevic et al. (2013) | 6 | 31.93 | DSM4 | SCID | 68 | 1 | 2 | 2 | 34 | NA | NA | |
| Allardyce et al. (2018) | 6 | 46 | RDC | SCAN | 4023 | unclear | 1 | 2 | 1945 | 1315 | 981 | |
| Millischer et al. (2020) | 6 | 46.6 | DSM4 | SCAN | 753 | 2 | 1 | 2 | 509 | 526 | 210 | |
| de Sousa et al. (2012) | 6 | 28.53 | DSM4TR | SCID | 141 | 3 | 2 | 0 | 64 | NA | NA | |
| Icick et al. (2022) | 7 | 36 | DSM4TR | mixed | 670 | unclear | 1 | 2 | 394 | NA | NA | |
| Chang et al. (2023) | 6 | 38.23 | DSM4TR | mixed | 9358 | 2 | 1 | 0 | 4218 | NA | NA | |
| Wingo et al. (2010) | 6 | 40.1 | DSM4 | SCID | 65 | 1 | 1 | 2 | 29 | NA | NA | |
| Sarrazin et al. (2018) | 6 | 40.46 | mixed | mixed | 211 | unclear | 2 | 2 | 129 | NA | NA | |
| Drachman et al. (2022) | 4 | 35.7 | DSM4 | SCID | 38 | unclear | 1 | 2 | 15 | NA | NA | |
| Thomas et al. (2008) | 5 | 43.54 | DSM4TR | YMRS | 120 | 3 | 2 | 0 | 87 | NA | NA | |
| Suppes et al. (2013) | 5 | 38.3 | DSM4TR | SCID | 55 | 3 | 0 | 2 | 4 | NA | NA | |
| Ng et al. (2021) | 5 | NA | ICD9 | unclear | 53434 | unclear | 1 | 0 | 9367 | NA | NA | |
| Gotra et al. (2020) | 4 | 37.69 | DSM4 | SCID | 259 | unclear | 1 | 2 | 192 | NA | NA | |
| Wagner-Skacel et al. (2020) | 5 | 47.4 | DSM4 | SCID | 46 | 1 | 1 | 2 | 6 | NA | NA | |
| Lee et al. (2017) | 6 | 43.94 | DSM4 | SCID | 68 | unclear | 1 | 2 | 22 | NA | NA | |
| Tohen et al. (1992) | 6 | 30 | DSM3 | DIS | 54 | 3 | 3 | 0 | 54 | 24 | 30 | |
| Bora et al. (2007) | 5 | 37.97 | DSM4 | SCID | 65 | unclear | 2 | 2 | 40 | NA | 18 | |
| Winokur et al. (1985) | 5 | NA | ICD8 | mixed | 30 | unclear | 1 | 0 | 13 | 12 | 3 | |
| Toni et al. (2001) | 7 | 37.9 | DSM3R | SAPS | 155 | 3 | 2 | 0 | 107 | 38 | 69 | |
| Benabarre et al. (2001) | 4 | NA | RDC | SADS | 67 | 1 | 2 | 1 | 28; 58 | NA | NA | |
| Allen et al. (2010) | 5 | 34.27 | DSM4 | SCID | 46 | unclear | 1 | 2 | 24 | NA | NA | |
| Harrow et al. (1982) | 3 | NA | RDC | mixed | 34 | 3 | 2 | 0 | 21 | NA | NA | |
| Gaudiano et al. (2007) | 6 | 39 | DSM | SCID | 74 | 3 | 2 | 1 | 59; 64 | 40 | 24 | |
| Parker et al. (2013) | 6 | 41.49 | DSM4 | DIGS | 41 | 2 | 2 | 2 | 25 | NA | NA | |
| Simonetti et al. (2023) | 7 | 46.23 | DSM5 | SCID | 360 | 1 | 1 | 2 | 164 | NA | NA | |
| Hett et al. (2023) | 8 | 54.26 | ICD10 | unclear | 2649 | 2 | 1 | 0 | 252 | NA | NA | |
| Menculini et al. (2023) | 7 | 44.76 | DSM5 | SCID | 178 | 2 | 1 | 2 | 82 | NA | NA | |
| Elowe et al. (2022) | 8 | 41.39 | DSM4 | DIGS | 162 | 2 | 2 | 2 | 96 | NA | 43 | |
| Bora et al. (2023) | 5 | NA | DSM4 | SCID | 90 | 1 | 1 | 2 | 56 | NA | NA | |
| Hassan et al. (2023) | 8 | 35.84 | DSM5 | SCID | 83 | 2 | 2 | 0 | 25 | NA | NA | |
| Chrobak et al. (2023) | 7 | 39.31 | mixed | unclear | 116 | 2 | 1 | 2 | 31 | NA | NA | |
| Dhiman et al. (2022) | 7 | 42.31 | DSM4 | MINIPLUS | 200 | 2 | 2 | 2 | 97 | NA | NA | |
| Fredriksen et al. (2022) | 8 | 43.7 | ICD10 | unclear | 226 | 3 | 1 | 0 | 45 | NA | NA | |
| Buoli et al. (2022) | 9 | 48.62 | DSM4TR | MINI | 1673 | 2 | 1 | 1 | 407; 780 | NA | NA | |
| Cote-Allard et al. (2022) | 7 | 43.48 | ICD10 | unclear | 58 | 2 | 1 | 0 | 17 | NA | NA | |

Note: The JBI score denotes study quality based on the JBI checklist (range = 0–9). Pop indicates patient population (1 = outpatient, 2 = mixed, 3 = inpatient). Criteria specify the diagnostic criteria used; Assessment refers to the interview tools employed (see Table S6). BD represents bipolar disorder subtype (0 = BD II, 1 = BSD, 2 = BD I, 3 = psychotic BD). TF indicates timeframe (0 = current, 1 = both, 2 = lifetime). Co (n) denotes the number of patients with the specified condition. Co MC (n) and Co MIC (n) denote the numbers of patients with mood-congruent and mood-incongruent psychosis, respectively. NA or “Unclear” within cells indicates that the value was not reported.

**Reference**

**Table S10**

*Included* studies in the synthesis of cooccurring delusions

| Study | JBI score | Age | Criteria | Assessment | N | Pop | BD | TF | CoTyp | Co (n) |
| --- | --- | --- | --- | --- | --- | --- | --- | --- | --- | --- |
| Asaad et al. (2014) | 7 | 34.65 | DSM4 | SCID | 350 | unclear | 1 | 0 | grandiosity | 156 |
| Swann et al. (1997) | 4 | 39 | DSM3 | SADS | 178 | 3 | 2 | 0 | any | 42 |
| Black and Nasrallah (1989) | 5 | NA | DSM3 | unclear | 628 | 3 | 1 | 0 | any | 227 |
| Mancuso et al. (2015) | 7 | 40.75 | ICD10 | DIP | 319 | unclear | 3 | 0 | any | 317 |
| Benazzi (1999) | 7 | 48 | DSM4 | SCID-C | 30 | 1 | 3 | 0 | any | 29 |
| Baethge et al. (2005) | 7 | NA | ICD | AMDP | 549 | 3 | 1 | 0 | any | 144 |
| Verdoux and Bourgeois (1993) | 5 | 35.17 | DSM3R | unclear | 36 | 3 | 3 | 0 | grandiosity | 29 |
| Robinson (1988) | 4 | NA | ICD9 | unclear | 35 | 3 | 2 | 0 | any | 18 |
| Breslau and Meltzer (1988) | 5 | 32.9 | RDC | SADS-C | 38 | 3 | 1 | 0 | persecutory | 20 |
| Song et al. (2015) | 3 | 34.8 | DSM4 | SCID | 44 | 2 | 3 | 0 | any | 28 |
| Das and Khanna (1993) | 5 | 31.93 | DSM3R | unclear | 60 | 3 | 2 | 0 | grandiosity | 26 |
| Braunig et al. (2009) | 6 | 41.27 | DSM4 | AMDP | 246 | unclear | 2 | 0 | any | 118 |
| Abrams and Taylor (1981) | 6 | 40.32 | feighner | unclear | 111 | 3 | 2 | 0 | persecutory | 37 |
| Prakash et al. (2009) | 5 | 68.5 | ICD10 | unclear | 30 | 1 | 2 | 0 | any | 8 |
| Nisha et al. (2015) | 6 | 39.1 | DCR10 | unclear | 30 | 3 | 1 | 0 | any | 23 |
| Sethi and Khanna (1993) | 6 | 27.7 | DSM3R | PSE | 100 | unclear | 2 | 0 | grandiosity | 93 |
| Canuso et al. (2008) | 4 | 36.84 | DSM4 | unclear | 515 | unclear | 2 | 0 | grandiosity | 319 |
| Winokur (1984) | 4 | NA | unclear | unclear | 122 | 3 | 1 | 0 | any | 66 |
| Stein et al. (2020) | 6 | 41.72 | DSM4 | SAPS | 151 | 2 | 1 | 0 | any | 22 |
| Grunebaum et al. (2001) | 5 | 35.8 | DSM4 | SAPS | 56 | unclear | 1 | 0 | any | 23 |
| Brancati et al. (2021) | 5 | 48.4 | DSM5 | CGI | 670 | 3 | 1 | 0 | suspiciousness | 54 |
| Schurhoff et al. (2003) | 6 | 41.14 | DSM4 | PDI | 61 | 3 | 1 | 0 | suspiciousness | 39 |
| Gorgulu et al. (2021) | 5 | 41.94 | DSM5 | unclear | 80 | 2 | 2 | 0 | any | 44 |
| Nakimuli-Mpungu et al. (2006) | 6 | 25.2 | DSM4 | unclear | 64 | 3 | 2 | 0 | paranoia | 51 |
| Reininghaus et al. (2016) | 7 | 48 | RDC | OPCRIT | 477 | 2 | 1 | 0 | grandiosity | 266 |
| Picardi et al. (2018) | 5 | 39.7 | mixed | BPRS | 217 | 3 | 3 | 0 | any | 61 |
| Jester et al. (2018) | 4 | NA | unclear | MDS | 23130 | unclear | 1 | 0 | any | 1234 |
| Rosen et al. (2016) | 4 | NA | DSM4TR | SCID | 33 | unclear | 3 | 0 | any | 31 |
| Toomey et al. (1998) | 6 | 47.7 | unclear | SADS | 58 | 2 | 3 | 0 | any | 12 |
| Gosek et al. (2019) | 4 | 48.91 | ICD10 | goesk et al | 70 | 3 | 1 | 0 | any | 9 |
| Morgan et al. (2005) | 7 | 42.47 | ICD10 | DIP | 112 | 2 | 1 | 0 | any | 23 |
| Caldieraro et al. (2017) | 5 | 39.45 | DSM4TR | BISS | 303 | 1 | 1 | 0 | any | 21 |
| Fekadu et al. (2004) | 6 | 27 | ICD10 | SCAN | 31 | 1 | 1 | 0 | grandiosity | 24 |
| Kennedy et al. (2004) | 4 | NA | DSM4 | OPCRIT | 246 | 2 | 2 | 0 | grandiosity | 136 |
| Chaudhary et al. (2021) | 4 | NA | DSM4TR | SCID | 188 | 3 | 1 | 0 | any | 69 |
| Parker et al. (2000) | 4 | 46.3 | DSM | CORE | 83 | 2 | 1 | 0 | any | 15 |
| Yan et al. (1982) | 6 | 31.51 | mixed | unclear | 108 | 3 | 2 | 0 | grandiosity | 80 |
| Carlson et al. (2012) | 6 | 29.3 | DSM3R | SCID | 126 | 3 | 3 | 0 | paranoia | 59 |
| Tohen et al. (1992) | 6 | 30 | DSM3 | DIS | 54 | 3 | 3 | 0 | paranoia | 35 |
| Lish et al. (1994) | 5 | NA | unclear | DMDA | 494 | unclear | 1 | 0 | paranoia | 46 |
| Bora et al. (2007) | 5 | 37.97 | DSM4 | SCID | 65 | unclear | 2 | 0 | any | 40 |
| Winokur et al. (1985) | 5 | NA | ICD8 | mixed | 30 | unclear | 1 | 0 | any | 13 |
| Conus et al. (2004) | 4 | 22.2 | DSM3R | BPRS | 87 | unclear | 3 | 0 | grandiosity | 77 |
| Toni et al. (2001) | 7 | 37.9 | DSM3R | SAPS | 155 | 3 | 2 | 0 | reference | 64 |
| Brockington et al. (1982) | 4 | NA | RDC | PSE | 32 | unclear | 1 | 0 | reference | 5 |
| Gaudiano et al. (2007) | 6 | 39 | DSM | SCID | 74 | 3 | 2 | 0 | grandiosity | 40 |
| Morgan et al. (2012) | 5 | NA | ICD10 | DIP | 319 | NA | 3 | 0 | any | 85 |
| Keck et al. (2003) | 7 | 41.32 | DSM4 | SCID-P | 352 | 1 | 2 | 1 | reference | 148 |
| Cassano et al. (2004) | 4 | 43.7 | mixed | MINI | 106 | 2 | 2 | 1 | persecutory | 59 |
| Serretti et al. (2002) | 7 | 44.36 | DSM | OPCRIT | 576 | 2 | 1 | 1 | any | 360 |
| Burton et al. (2018) | 6 | 40.9 | DSM4 | DIGS | 381 | unclear | 1 | 1 | any | 142 |
| Mancuso et al. (2015) | 7 | 40.75 | ICD10 | DIP | 319 | unclear | 3 | 1 | any | 318 |
| Upthegrove et al. (2015) | 6 | 47 | DSM4 | SCAN | 2019 | unclear | 2 | 1 | any | 1308 |
| Garcia-Gonzalez et al. (2020) | 5 | 55.93 | unclear | CIDI | 781 | unclear | 1 | 1 | any | 255 |
| Schulze et al. (2002) | 5 | 43.02 | DSM4 | mixed | 90 | 3 | 1 | 1 | any | 39 |
| Tost et al. (2010) | 6 | 42.4 | DSM4 | SCID | 42 | 3 | 2 | 1 | persecutory | 15 |
| Akinhanmi et al. (2020) | 5 | 41.81 | unclear | DIGS | 843 | unclear | 2 | 1 | any | 419 |
| Skowronek et al. (2006) | 4 | 42.4 | DSM4 | unclear | 306 | unclear | 1 | 1 | persecutory | 107 |
| Schulze et al. (2002) | 7 | 44.58 | DSM4 | mixed | 594 | unclear | 1 | 1 | persecutory | 145 |
| Perlman et al. (2016) | 5 | 44.35 | DSM4 | DI-PAD | 2084 | unclear | 1 | 1 | persecutory and jealous | 285 |
| Poletti et al. (2015) | 5 | 47.62 | DSM4 | SCID | 78 | unclear | 2 | 1 | any | 28 |
| Stein et al. (2020) | 6 | 41.72 | DSM4 | SAPS | 151 | 2 | 1 | 1 | any | 36 |
| Song et al. (2012) | 6 | 32.2 | DSM4 | DIGS | 212 | unclear | 1 | 1 | any | 97 |
| Ekman et al. (2017) | 7 | 38.47 | DSM4TR | mixed | 167 | 1 | 1 | 1 | any | 77 |
| Schutte et al. (2022) | 5 | 48.31 | DSM4 | CASH | 113 | unclear | 1 | 1 | any | 63 |
| Morgan et al. (2005) | 7 | 42.47 | ICD10 | DIP | 112 | 2 | 1 | 1 | any | 96 |
| van Bergen et al. (2019) | 7 | 49.5 | DSM4 | SCID | 1342 | 2 | 2 | 1 | any | 925 |
| Kennedy et al. (2004) | 4 | NA | DSM4 | OPCRIT | 246 | 2 | 2 | 1 | any | 143 |
| Musetti et al. (2018) | 7 | 38.6 | DSM4 | unclear | 234 | 1 | 1 | 1 | any | 99 |
| Ongur et al. (2009) | 7 | 36.4 | DSM4TR | SCID | 92 | 2 | 3 | 1 | any | 89 |
| Baryshnikov et al. (2018) | 4 | 43.7 | mixed | CAPE-42 | 99 | 2 | 1 | 1 | grandiosity | 77 |
| Parker et al. (2013) | 6 | 35.73 | DSM4 | mixed | 632 | unclear | 1 | 1 | any | 93 |
| Dell'Osso et al. (2002) | 7 | 36.48 | DSM3R | SCID-P | 147 | 3 | 3 | 1 | any | 146 |
| Benabarre et al. (2001) | 4 | NA | RDC | SADS | 67 | 1 | 2 | 1 | any | 55 |
| Baek et al. (2011) | 6 | 34.14 | DSM4 | DIGS | 105 | 2 | 1 | 1 | any | 24 |
| Pfohl et al. (1982) | 5 | 35.25 | DSM3 | unclear | 247 | 3 | 2 | 1 | any | 150 |
| Rajkumar (2016) | 6 | 31.64 | DSM4 | MINI | 66 | unclear | 2 | 1 | any | 42 |
| Park et al. (2014) | 6 | 41.49 | DSM4 | DIGS | 41 | 2 | 2 | 1 | any | 19 |
| Etain et al. (2017) | 5 | 43 | DSM4 | PDI | 270 | unclear | 1 | 1 | suspiciousness | 168 |
| Elowe et al. (2022) | 8 | 41.39 | DSM4 | DIGS | 162 | 2 | 2 | 1 | any | 87 |
| Morgan et al. (2012) | 5 | NA | ICD10 | DIP | 319 | NA | 3 | 1 | any | 265 |

*Note*. The JBI score refers to the study quality score rated by the JBI checklist (range = 0-9). Pop denotes the patient population (1 = outpatient, 2 = mixed, 3 = inpatient). Criteria specify the diagnostic criteria used, while Assessment refers to the interview tools employed (See Table S6 for details). BD represents the bipolar diagnosis subtype (0 = BD II, 1 = BSD, 2 = BD I, 3 = Psychotic BD). TF indicates the timeframe (0 = current, 1 = lifetime). Cotyp refers to the type of cooccurring delusions (e.g., any delusions, or specific delusion such as grandiosity and idea of reference etc.). Co (n): number of patients with this condition.

**Table 11**

*Included studies in the synthesis of* cooccurring hallucinations

| Study | JBI score | Age | Criteria | Assessment | N | Pop | BD | TF | CoTyp | Co (n) |
| --- | --- | --- | --- | --- | --- | --- | --- | --- | --- | --- |
| *Asaad et al. (2014)* | 7 | 34.65 | DSM4 | SCID | 350 | unclear | 1 | *0* | auditory | 113 |
| *Swann et al. (1997)* | 4 | 39 | DSM3 | SADS | 178 | 3 | 2 | *0* | any | 37 |
| *Black and Nasrallah (1989)* | 5 | NA | DSM3 | unclear | 628 | 3 | 1 | *0* | any | 80 |
| *Mancuso et al. (2015)* | 7 | 40.75 | ICD10 | DIP | 319 | unclear | 3 | *0* | any | 61 |
| *Benazzi (1999)* | 7 | 48 | DSM4 | SCID-C | 30 | 1 | 3 | *0* | any | 5 |
| *Baethge et al. (2005)* | 7 | NA | ICD | AMDP | 549 | 3 | 1 | *0* | any | 65 |
| *Verdoux and Bourgeois (1993)* | 5 | 35.17 | DSM3R | unclear | 36 | 3 | 3 | *0* | any | 11 |
| *Breslau and Meltzer (1988)* | 5 | 32.9 | RDC | SADS-C | 38 | 3 | 1 | *0* | auditory | 7 |
| *Song et al. (2015)* | 3 | 34.8 | DSM4 | SCID | 44 | 2 | 3 | *0* | any | 19 |
| *Das and Khanna (1993)* | 5 | 31.93 | DSM3R | unclear | 60 | 3 | 2 | *0* | mc | 1 |
| *Olfson et al. (2005)* | 6 | NA | DSM4 | MINI | 53 | 1 | 1 | *0* | any | 19 |
| *Braunig et al. (2009)* | 6 | 41.27 | DSM4 | AMDP | 246 | unclear | 2 | *0* | any | 113 |
| *Abrams and Taylor (1981)* | 6 | 40.32 | feighner | unclear | 111 | 3 | 2 | *0* | auditory | 22 |
| *Prakash et al. (2009)* | 5 | 68.5 | ICD10 | unclear | 30 | 1 | 2 | *0* | any | 3 |
| *Nisha et al. (2015)* | 6 | 39.1 | DCR10 | unclear | 30 | 3 | 1 | *0* | any | 15 |
| *Winokur et al. (1985)* | 4 | NA | unclear | unclear | 122 | 3 | 1 | *0* | auditory | 17 |
| *Stein et al. (2020)* | 6 | 41.72 | DSM4 | SAPS | 151 | 2 | 1 | *0* | any | 11 |
| *Morgan et al. (2012)* | 5 | NA | ICD10 | DIP | 319 | NA | 3 | *0* | any | 217 |
| *Brancati et al. (2021)* | 5 | 48.4 | DSM5 | CGI | 670 | 3 | 1 | *0* | any | 157 |
| *Schurhoff et al. (2003)* | 6 | 41.14 | DSM4 | PDI | 61 | 3 | 1 | *0* | auditory | 7 |
| *Mirza et al. (2021)* | 5 | NA | ICD10 | HoNOS | 3379 | 2 | 1 | *0* | any | 1063 |
| *Morch-Johnsen et al. (2018)* | 6 | 34.5 | DSM4 | SCID | 157 | 1 | 1 | *0* | auditory | 22 |
| *Gorgulu et al. (2021)* | 5 | 41.94 | DSM5 | unclear | 80 | 2 | 2 | *0* | any | 16 |
| *Nakimuli-Mpungu et al. (2006)* | 6 | 25.2 | DSM4 | unclear | 64 | 3 | 2 | *0* | auditory | 10 |
| *Reininghaus et al. (2016)* | 7 | 48 | RDC | OPCRIT | 477 | 2 | 1 | *0* | any | 0 |
| *Jester et al. (2018)* | 4 | NA | unclear | MDS | 23130 | unclear | 1 | *0* | any | 745 |
| *Rosen et al. (2016)* | 4 | NA | DSM4TR | SCID | 33 | unclear | 3 | *0* | auditory | 26 |
| *Toomey et al. (1998)* | 6 | 47.7 | unclear | SADS | 58 | 2 | 3 | *0* | any | 0 |
| *Morgan et al. (2005)* | 7 | 42.47 | ICD10 | DIP | 112 | 2 | 3 | *0* | any | 4 |
| *Caldieraro et al. (2017)* | 5 | 39.45 | DSM4TR | BISS | 303 | 1 | 1 | *0* | any | 22 |
| *Fekadu et al. (2004)* | 6 | 27 | ICD10 | SCAN | 31 | 1 | 1 | *0* | any | 3 |
| *Kennedy et al. (2004)* | 4 | NA | DSM4 | OPCRIT | 246 | 2 | 2 | *0* | auditory | 41 |
| *Parker et al. (2000)* | 4 | 46.3 | DSM | CORE | 83 | 2 | 1 | *0* | any | 7 |
| *Yan et al. (1982)* | 6 | 31.51 | mixed | unclear | 108 | 3 | 2 | *0* | auditory | 14 |
| *Carlson et al. (2012)* | 6 | 29.3 | DSM3R | SCID | 126 | 3 | 3 | *0* | any | 64 |
| *Tohen et al. (1992)* | 6 | 30 | DSM3 | DIS | 54 | 3 | 3 | *0* | auditory | 16 |
| *Winokur et al. (1985)* | 5 | NA | ICD8 | mixed | 30 | unclear | 1 | *0* | any | 8 |
| *Conus et al. (2004)* | 4 | 22.2 | DSM3R | BPRS | 87 | unclear | 3 | *0* | any | 48 |
| *Toni et al. (2001)* | 7 | 37.9 | DSM3R | SAPS | 155 | 3 | 2 | *0* | auditory | 18 |
| *Brockington et al. (1982)* | 4 | NA | RDC | PSE | 32 | unclear | 1 | *0* | auditory | 3 |
| *Gaudiano et al. (2007)* | 6 | 39 | DSM | SCID | 74 | 3 | 2 | *0* | auditory | 25 |
| *Morgan et al. (2022)* | 6 | 38.45 | DSM5 | EMA rating | 114 | 2 | 1 | *0* | auditory | 9 |
| *Keck et al. (2003)* | 7 | 41.32 | DSM4 | SCID-P | 2 | 1 | 2 | *1* | auditory | 88 |
| *Cassano et al. (2004)* | 4 | 43.7 | mixed | MINI | 2 | 2 | 2 | *1* | auditory | 22 |
| *Serretti et al. (2002)* | 7 | 44.36 | DSM | OPCRIT | 1 | 2 | 1 | *1* | any | 168 |
| *Burton et al. (2018)* | 6 | 40.9 | DSM4 | DIGS | 1 | unclear | 1 | *1* | any | 88 |
| *Mancuso et al. (2015)* | 7 | 40.75 | ICD10 | DIP | 3 | unclear | 3 | *1* | any | 217 |
| *Upthegrove et al. (2015)* | 6 | 47 | DSM4 | SCAN | 2 | unclear | 2 | *1* | auditory | 469 |
| *Garcia-Gonzalez et al. (2020)* | 5 | 55.93 | unclear | CIDI | 1 | unclear | 1 | *1* | any | 268 |
| *Schulze et al. (2002)* | 5 | 43.02 | DSM4 | mixed | 1 | 3 | 1 | *1* | any | 20 |
| *Tost et al. (2010)* | 6 | 42.4 | DSM4 | SCID | 2 | 3 | 2 | *1* | any | 8 |
| *Olfson et al. (2005)* | 6 | NA | DSM4 | MINI | 1 | 1 | 1 | *1* | any | 38 |
| *Akinhanmi et al. (2020)* | 5 | 41.81 | unclear | DIGS | 2 | unclear | 2 | *1* | any | 212 |
| *Perlman et al. (2016)* | 5 | 44.35 | DSM4 | DI-PAD | 1 | unclear | 1 | *1* | any | 514 |
| *Stein et al. (2020)* | 6 | 41.72 | DSM4 | SAPS | 1 | 2 | 1 | *1* | any | 15 |
| *Hwang et al. (2021)* | 4 | NA | DSM4TR | SCID | 1 | 2 | 1 | *1* | auditory | 22 |
| *Chouinard et al. (2019)* | 4 | 36.5 | DSM4TR | SCID | 3 | 2 | 3 | *1* | visual | 66 |
| *Song et al. (2012)* | 6 | 32.2 | DSM4 | DIGS | 1 | unclear | 1 | *1* | auditory | 65 |
| *Ekman et al. (2017)* | 7 | 38.47 | DSM4TR | mixed | 1 | 1 | 1 | *1* | any | 40 |
| *Lewandowski et al. (2009)* | 6 | NA | DSM4TR | SCID | 2 | 2 | 2 | *1* | any | 77 |
| *Morch-Johnsen et al. (2018)* | 6 | 34.5 | DSM4 | SCID | 1 | 1 | 1 | *1* | auditory | 49 |
| *Hammersley et al. (2003)* | 5 | 40.5 | DSM4 | SCID | 1 | unclear | 1 | *1* | any | 45 |
| *Zenisek et al. (2015)* | 4 | 35.89 | DSM4 | SCID | 1 | 1 | 1 | *1* | auditory | 17 |
| *Schutte et al. (2022)* | 5 | 48.31 | DSM4 | CASH | 1 | unclear | 1 | *1* | any | 73 |
| *Morgan et al. (2005)* | 7 | 42.47 | ICD10 | DIP | 3 | 2 | 3 | *1* | any | 23 |
| *van Bergen et al. (2019)* | 7 | 49.5 | DSM4 | SCID | 2 | 2 | 2 | *1* | any | 573 |
| *Kennedy et al. (2004)* | 4 | NA | DSM4 | OPCRIT | 2 | 2 | 2 | *1* | auditory | 42 |
| *Ongur et al. (2009)* | 7 | 36.4 | DSM4TR | SCID | 3 | 2 | 3 | *1* | any | 44 |
| *Parker et al. (2013)* | 6 | 35.73 | DSM4 | mixed | 1 | unclear | 1 | *1* | any | 92 |
| *Benabarre et al. (2001)* | 4 | NA | RDC | SADS | 2 | 1 | 2 | *1* | any | 23 |
| *Baek et al. (2011)* | 6 | 34.14 | DSM4 | DIGS | 1 | 2 | 1 | *1* | any | 13 |
| *Pfohl et al. (1982)* | 5 | 35.25 | DSM3 | unclear | 2 | 3 | 2 | *1* | any | 75 |
| *Rajkumar (2016)* | 6 | 31.64 | DSM4 | MINI | 2 | unclear | 2 | *1* | any | 12 |
| *Park et al. (2014)* | 6 | 41.49 | DSM4 | DIGS | 2 | 2 | 2 | *1* | any | 14 |
| *Elowe et al. (2022)* | 8 | 41.39 | DSM4 | DIGS | 2 | 2 | 2 | *1* | any | 50 |
| *Morgan et al. (2012)* | 5 | NA | ICD10 | DIP | 3 | NA | 3 | *1* | any | 217 |

*Note*. The JBI score refers to the study quality score rated by the JBI checklist (range = 0-9). Pop denotes the patient population (1 = outpatient, 2 = mixed, 3 = inpatient). Criteria specify the diagnostic criteria used, while Assessment refers to the interview tools employed (See Table S6 for details). BD represents the bipolar diagnosis subtype (0 = BD II, 1 = BSD, 2 = BD I, 3 = Psychotic BD). TF indicates the timeframe (0 = current, 1 = lifetime). Cotyp refers to the type of cooccurring hallucinations (e.g., any hallucinations, mc: mood congruent hallucinations, or auditory hallucinations). Co (n): number of patients with this condition.

Reference:

**Table S12**

*Included studies in the synthesis of cooccurring* thought disorders

| Study | JBI score | Age | Criteria | Assessment | N | Pop | BD | TF | CoTyp | Co (n) |
| --- | --- | --- | --- | --- | --- | --- | --- | --- | --- | --- |
| *Mancuso et al. (2015)* | 7 | 40.75 | ICD10 | DIP | 319 | unclear | 3 | *0* | any | 36 |
| *Breslau and Meltzer (1988)* | 5 | 32.9 | RDC | SADS-C | 38 | 3 | 1 | *0* | any | 16 |
| *Marengo and Harrow (1985)* | 4 | 23 | mixed | CMBT | 38 | 3 | 2 | *0* | any | 28 |
| *Abrams and Taylor (1981)* | 6 | 40.32 | feighner | unclear | 111 | 3 | 2 | *0* | ftd | 9 |
| *Stein et al. (2020)* | 6 | 41.72 | DSM4 | SAPS | 151 | 2 | 1 | *0* | pftd | 62 |
| *Reininghaus et al. (2016)* | 7 | 48 | RDC | OPCRIT | 477 | 2 | 1 | *0* | pftd | 0 |
| *Taylor et al. (1994)* | 5 | 55.27 | DSM3 | mixed | 62 | unclear | 2 | *0* | ftd | 4 |
| *Toomey et al. (1998)* | 6 | 47.7 | unclear | SADS | 58 | 2 | 3 | *0* | any | 20 |
| *Keck et al. (2003)* | 7 | 41.32 | DSM4 | SCID-P | 352 | 1 | 2 | *1* | ds | 21 |
| *Mancuso et al. (2015)* | 7 | 40.75 | ICD10 | DIP | 319 | unclear | 3 | *1* | any | 105 |
| *Perlman et al. (2016)* | 5 | 44.35 | DSM4 | DI-PAD | 2084 | unclear | 1 | *1* | pftd | 275 |
| *Stein et al. (2020)* | 6 | 41.72 | DSM4 | SAPS | 151 | 2 | 1 | *1* | pftd | 70 |
| *van Bergen et al. (2019)* | 7 | 49.5 | DSM4 | SCID | 1342 | 2 | 2 | *1* | any | 801 |
| *Kennedy et al. (2004)* | 4 | NA | DSM4 | OPCRIT | 246 | 2 | 2 | *1* | any | 89 |
| *Pfohl et al. (1982)* | 5 | 35.25 | DSM3 | unclear | 247 | 3 | 2 | *1* | ftd | 143 |
| *Park et al. (2014)* | 6 | 41.49 | DSM4 | DIGS | 41 | 2 | 2 | *1* | dtb | 2 |

*Note.* The JBI score refers to the study quality score rated by the JBI checklist (range = 0-9). Pop denotes the patient population (1 = outpatient, 2 = mixed, 3 = inpatient). Criteria specify the diagnostic criteria used, while Assessment refers to the interview tools employed (See Table S6 for details). BD represents the bipolar diagnosis subtype (0 = BD II, 1 = BSD, 2 = BD I, 3 = Psychotic BD). TF indicates the timeframe (0 = current, 1 = lifetime). Cotyp refers to the type of cooccurring thought disorders (e.g., ftd= formal thought disorder, ds = disturbed speech, dtb = disorganised thoughts and behaviours, pftd = positive formal thought disorder). Co (n): number of patients with this condition.

Reference:
